# Kinglet in the Poultry Court of Russia: Whole-Genome Insights into Ancestry, Genetic Variability, Selection Footprints and Candidate Genes in a Unique Local Chicken Breed Relative to Other Bantam/Dwarf Breeds

**DOI:** 10.3390/ani16040642

**Published:** 2026-02-17

**Authors:** Natalia V. Dementieva, Yuri S. Shcherbakov, Anatoli B. Vakhrameev, Michael N. Romanov

**Affiliations:** 1 Russian Research Institute of Farm Animal Genetics and Breeding—Branch of the L. K. Ernst Federal Science Center for Animal Husbandry, Pushkin, Saint Petersburg 196601, Russia; dementevan@mail.ru (N.V.D.); yura.10.08.94.94@mail.ru (Y.S.S.); ab_poultry@mail.ru (A.B.V.); 2 L. K. Ernst Federal Research Center for Animal Husbandry, Dubrovitsy, Podolsk 142132, Moscow Oblast, Russia

**Keywords:** chicken (*Gallus gallus*), native breeds, Russian Korolyok, bantam/dwarf breeds, whole-genome genotyping, single-nucleotide polymorphisms (SNPs), genetic diversity, runs of homozygosity (ROHs), candidate genes

## Abstract

Assessing genetic diversity in various native poultry breeds, including bantam/dwarf ones, is instrumental for their conservation as genetic resources, identifying their specific genetic features, and exploring the history of their genetic divergence. Rare chicken breeds are usually carriers of peculiar phenotypic traits, including adaptations to local conditions, disease resistance, and unique performance features. Here, we report for the first time SNP-based genetic characterization of the Russian Korolyok, translated as “kinglet,” relative to five other dwarf/small breeds: Cochin Bantam, Hamburg Bantam Silver Spangled, Polish White-crested Black, Red White-tailed Dwarf and Silkie White. We estimated phenotypes, heterozygosity, inbreeding, effective population size, and runs of homozygosity (ROHs). Some breeds had higher genetic diversity and others showed elevated inbreeding rates in their genomes. With lower effective population sizes (both presently and in the past), rare breeds came from a limited number of ancestors or were under strong selection pressure over many generations. Within 22 ROHs, we identified 26 prioritized candidate genes (*GRB10*, *RPRD1A*, *APOOL*, *EAF2*, *SEMA5*, *HACD2*, *GALANT1*, *DACH2*, *CHM*, *POF1B*, *HDX*, *SLC15A2*, *PDIA5*, *SEC22*, *NR2F2*, *ARRDC4*, *IGF1R*, *SYNM*, *TMEM263*, etc.). Our data offer whole-genome insights into genetic variability, history, phylogeny, selective sweeps, and candidate genes of a distinct indigenous Russian chicken breed and other bantam/dwarf breeds.

## Simple Summary

Rare local chicken breeds, including bantam/dwarf ones, were, at a certain breeding stage, subject to strong selection for a specific appearance for many generations. This led to adaptation to local conditions, disease resistance and preservation of unique breed characteristics, creating genetic divergence between breeds. Assessing the genetic diversity of various native poultry breeds is crucial for their conservation as genetic resources. One of the rare old Russian chicken breeds is the Korolyok, meaning “kinglet” in Russian. Local dwarf chickens were mentioned in the first Russian book on poultry farming published in 1774 and seen by Pallas in Russia at about the same time. Here, we present for the first time the genetic characteristics of this unique local breed compared to five other dwarf/small chicken breeds. Using whole-genome genotyping, we determined descriptive characteristics of genetic diversity and comprehensively assessed the significance of genetic similarities/differences within and between these breeds. A number of intra-breed population structure patterns and selective signatures were found that were associated with the history of the breed’s formation and subsequent breeding, while harboring relevant candidate genes. The low population size of these breeds, as well as their declining reproductive capacity, may indicate the risk of their extinction.

## 1. Introduction

Chicken (*Gallus gallus*; GGA) breeds exhibit significant diversity, while the complex history of domestication and breed formation complicates the study of genetic variation in relation to their phenotypes. Body size is an economically important trait for both meat and egg-laying poultry. Small-sized layers significantly reduce feed consumption compared to standard layers [1]. Due to this advantageous phenotypic trait, examples of the use of chickens carrying GGAZ-linked or autosomal dwarfism genes (*DW* or *ADW*, respectively; [2,3,4]) in commercial and experimental crosses and lines are known. Such examples include, among others, Dwarf Brown- and Dwarf Layer White-shell strains obtained by crossing a Chinese local breed and a synthetic line from a French Star Broiler Company [5,6], dwarf recessive white-feathered broiler chickens from a cross between a Chinese breed and a recessive white-feathered Star C line from the same French Star Broiler Company [7], a Chinese white tail dwarf layer breed used for crossing with Rhode Island Reds [8], a Bangladeshi local dwarf breed crossed with Rhode Island Reds, White Leghorns and Fayoumi [9], etc.

Historically, small chickens arose as ornamental breeds. These primarily include the so-called true bantams [10,11,12], whose characteristic features include long drooping wings, a short back, and a tail often raised vertically. Bantams originate from Indonesia and received their name from the port city of Banten (Bantam) in the western part of Java, from where Dutch sailors brought them to Europe in the 17th century [10,13,14,15,16,17]. In the 18th century, these small chickens were already well known in Russia. In particular, they were first mentioned by the Russian statesman and encyclopedist Nikolai G. Teplov (1711–1779) in the book *The Poultry Yard* published in 1774 [18]: “*A breed of very small chickens, which in our country are called Dwarfs, is imported from Holland. They are said to be of African origin [an unverified claim of that time], yet I have observed them to be more prolific than any other kind. The cocks are proud and spirited, jealous and possessive of their hens, and they often attack larger roosters, whom they defeat through their lightness and agility. The hens lay more eggs than those of any other breed and sit firmly on their nests. Their flesh is tender and of excellent taste, while their varied plumage adds great ornament to the poultry yard. A great number of these birds are bred in France, especially in the province of Brittany. Two varieties of them are known to me and kept in my own poultry yard: one consists of birds that are very slender, swift, and nimble* [this matches the description of true bantams]; *the other of short-legged, feather-footed fowl that move by creeping or hopping rather than by walking [perhaps similar to the Dutch Booted Bantam, or Sabelpoot]*”. Some later authors (e.g., [19,20]) erroneously attributed the above description of the first Teplov’s dwarf variety to the Korolyok breed. During the same historical period, Russian zoography [21] was thoroughly studied by the German zoologist and Russian academician Peter Simon Pallas (1741–1811) in his extensive expeditions (1768–1774, 1793–1794) at the invitation of Empress Catherine II [13,22,23,24,25]. Among the various varieties of Russian chickens referenced in his works, he described “*dwarf English chickens, with feathered legs; hens are usually white, roosters are ginger-mottled*”. Furthermore, the mention of bantams elsewhere in the same work certainly shows Pallas’s familiarity with Indonesian ornamental forms imported into Europe. Thus, Russian bantams should be considered among the oldest aboriginal breeds known for a long time in Russia, along with the Orloff [26,27,28,29,30], Pavlov [24,25,31], Yurlov [32,33,34], Ushanka [35] and Poltava breeds [36,37,38,39,40,41].

Interestingly, Charles Darwin (1809–1882) compiled detailed information in 1868 [42] on the appearance, productivity, osteological characteristics, and breeds of bantams. Citing Crawfurd [43] (p. 113), he reported that bantams originated in Japan, “*are mentioned in an ancient native Japanese Encyclopaedia*” as “*a dwarf fowl, probably the true Bantam*,” and “*were imported from Japan into Bantam in Java*”.

Subsequently, the smallest chickens in Russia were called “korolki” (this can be translated as kinglets), since they could be compared with the smallest birds in Russian forests—kinglets (*Regulus* spp., Passeriformes; even described in Russia by Pallas [21] under their Russian name *korolok*)—as follows: “*how small, well, like our kinglets*” [20]. Later, this chicken breed began to be written about as “Russian Korolyok” (RK), or “Kursk Pestrushka” [44,45]. According to the Russian poultry geneticist Irina G. Moiseyeva (1932–2015) [44,45], who studied the origin and gene pool of domestic chicken breeds, RK appeared as a result of crossing bantams (of unknown breeds) and local chicken varieties. One of the founders of industrial poultry farming in Russia, Ivan I. Abozin (1846–1908) [19], also claimed the same origin for RK, considering English birds as possible foreign bantams imported and then mixed with Russian common chickens. At the same time, the German ornithologist August C.E. Baldamus (1812–1893) [11] believed that it would be more correct to understand English bantams as old European dwarfs in general.

Russian dwarf (or RK) chickens from the farm of the state councilor Alexander N. Golyashkin (1824–1880) were first presented at an acclimatization exhibition in Moscow in 1878 [20]. At the 1885 exhibition in Kharkov, RK chickens were already represented by two varieties (white and speckled gold) from the collection of another founder of Russian poultry farming, Alexander S. Batashev (1848–1912) [20].

**Figure 1:**
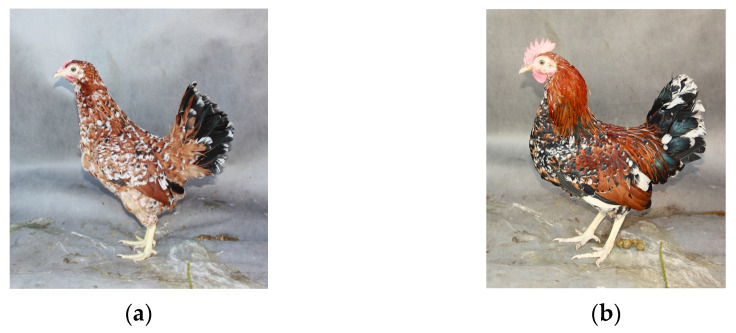
Appearance of the Russian Korolyok breed (of the mille fleur variety) at 210 days old: (**a**) a hen, and (**b**) a rooster.

Phenotypically, RK cockerels have a proud posture, a convex chest, and a vertically set, bushy tail with long main feathers (sickles). The comb is single, the earlobe is red, the shank is yellow, the wings are tightly pressed, and the tips are lowered and partially cover the tibia [46] (see Supplementary Information Box S1 for a detailed description; [47]). The plumage coloration of the RK population in the Bioresource Collection of Rare and Endangered Chicken Breeds (BCRECB) at the Russian Research Institute of Farm Animal Genetics and Breeding (RRIFAGB) is mille fleur (Figure 1). However, some variations in the color patterns of this breed are also possible (Supplementary Figure S1).

RK is characterized by specific production traits. In particular, the body weight (BW) of roosters is 0.8–0.9 kg and that of hens is 0.7–0.8 kg. Egg number (EN) is 80–90 eggs per year, with eggs weighing 30–35 g and the eggshell being white [47]. A study of morphotypological characteristics showed that RK bantams are closely related to the Pavlov breed and three varieties of the Shabo (Japanese Bantam) breed, although the Pavlov breed was quite distant from them [45].

Moiseyeva et al. [48,49] were the first to explore DNA polymorphism and electrophoretic analysis of proteins in RK. This study of RK was conducted along with examinations of two other old Russian breeds (Pavlov and Russian Black Bearded) and was based on blood and egg white samples. According to earlier studies by different authors, the RK population turned out to be a highly consolidated group for biochemical marker loci. However, the heterozygosity for these loci in RK was below the average level among all 52 breeds studied [44]. The listed historical facts and initial scientific information about these chickens, at different times known under different names, i.e., Russian Dwarf, Korolyok, and Kursk Pestrushka [44,45], as well as the Bantam Mille Fleur [50,51,52,53,54], explain the continued interest of many researchers and poultry fanciers in this autochthonous breed. In our preliminary genomic studies of BCRECB/RRIFAGB chickens using the Illumina Chicken 60K SNP iSelect BeadChip, we also noted consolidation and lower genetic diversity in the RK population, as well as divergence relative to other breeds [51].

A deeper understanding of the genetic basis of dwarf breeds, assessed at the whole-genome level, will facilitate the development and progress of small-scale breeding work in poultry farming. Interest in this issue has recently increased with the use of state-of-the-art genomic technologies, such as genome-wide association studies (GWASs) and RNA-seq data (e.g., [4,55]). Therefore, the aim of our study was a comparative examination of phenotypic and genetic variability, phylogeny, admixture, and the search for traces of selection and candidate genes in the genomes of RK and other similar dwarf breeds from the BCRECB/RRIFAGB collection of rare and endangered chicken breeds: Cochin Bantam (CB), Hamburg (or Hamburgh) Bantam Silver Spangled (HBSS), Polish White-crested Black (PWB), Red White-tailed Dwarf (RWD), and Silkie White (SW). Our findings demonstrate a certain phenotypic and genetic uniqueness of this Russian dwarf (bantam) breed, emphasizing the need for its further conservation.

## 2. Materials and Methods

### 2.1. Birds and Phenotypic Measurements

In this study, we included six dwarf breeds maintained in the BCRECB/RRIFAGB collection. Phenotypic parameters were collected from RK chickens (*n* = 40) and other similar dwarf breeds (Table 1): CB (or Pekin Bantam) Mottled (*n* = 54), HBSS (*n* = 25), PWB (known in Russia and some other countries as Holland/Dutch White-crested; *n* = 36), RWD (*n* = 19), and SW (or Silkie Bantam White; *n* = 44). The English names and descriptions of the breeds and varieties (except RK and RWD) were verified against Somes’ *International Registry of Poultry Genetic Stocks* [2] as listed there under the following numbers: 667, Cochin Bantam, Mottled; 762, Hamburg Bantam, Silver Spangled; 1006, Polish, White-crested Black; and 1058, Silkie Bantam, White. A total of 218 individuals were phenotyped.

The following phenotypic (including exterior) traits were measured (Table 1): BW, body length (BL), shank length (SL), shank diameter (SD), chest depth (CD), pectoral angle (PA), specific PA index (PA/BW), EN, egg weight (EW), and Narushin’s Integral Performance Index (IPI), as described elsewhere [52,53,54,56,57,58,59,60]. Using all combined quantitative phenotypic characteristics and the Phantasus web application (version 1.31.1; [61,62]), principal component analysis (PCA) and hierarchical clustering (HC) were employed to elucidate the differences between the studied chicken breeds. Normalization of the phenotypic index values was performed using log_2_ adjustment implemented in the Phantasus web tool. HC dendrograms were based on the Euclidean distance metric and on the matrix values for a precomputed distance matrix (with the *average* option selected for the clustering method).

### 2.2. Samples and DNA Isolation

This genome-wide study involved 116 blood samples collected from approximately 20 adult chickens per breed aged 270 days. Samples were taken from the inner wing vein in 0.5 mL microtubes containing 0.5 M ethylenediaminetetraacetate as an anticoagulant. DNA was isolated using phenol–chloroform extraction. A NanoDrop^TM^ 2000c spectrophotometer (Thermo Fisher Scientific, Waltham, MA, USA) was used to analyze the DNA concentration and purity of the collected samples. Samples with an A260/280 ratio between 1.7 and 2.0 were considered suitable for whole-genome genotyping.

### 2.3. SNP Genotyping

Whole-genome genotyping was performed using the Illumina Chicken 60K SNP iSelect BeadChip (Illumina, San Diego, CA, USA) with a coverage of 57,636 SNPs. The resultant whole-genome data were used to assess genetic diversity and other parameters based on DNA sequence polymorphism analysis. Quality control of genotyped SNP loci and a search for homozygous regions on individual chromosomes were performed using the PLINK software suite (version 1.9; [63,64]). The following SNP filtering parameters were applied to quality-control the genomic data: --maf, 0.05; --geno, 0.02; and --hwa, 0.0001. After filtering, 43,841 SNPs located on autosomes GGA1 through GGA28 were used for further analysis. SNP markers located on sex chromosomes were removed to eliminate the influence of sex on the assessment.

### 2.4. Genetic/Genomic Parameter Analyses

The calculation of genetic diversity indices (as described methodologically elsewhere, e.g., [65]) was performed in R (version 3.6.2; [66]) using the *diveRsity* package (version 1.9.90; [67]). The fixation indices (*F*_ST_) were calculated using the StaMPP library (version 1.6.3; [68]) in R based on the obtained SNP profiles of the studied chickens. Computation for the PCA analysis was performed in PLINK. The results were visualized in R using the plotly library (version 4.11.0; [69,70]).

Genetic distances were determined and phylogenetic dendrogram construction was performed using *F*_ST_ values and the Neighbor-Joining method in the SplitsTree program (version 6.3.40; [71]). The results were visualized using the ITOL online service (version 4; [72]). The population admixture analysis was performed using the admixture program (version 1.3; [73]), and the data for calculating the cross-validation (CV) error were also generated in it. The effective population size (*N*_e_) analysis was performed using the SNeP program (version 1.1; [74]).

To determine runs of homozygosity (ROHs), we employed the sequential SNP detection method implemented in RStudio (version 1.1.453; [75]) using the detectRUNS package (version 0.9.6; [76]). ROH islands were defined as homozygous regions overlapping in at least 75% of animals. To exclude common ROHs, a minimum length threshold of 500 Kb was set for a single ROH. To minimize false positive results, we calculated the minimum number of SNPs, which was 15. To avoid underestimation of the number of ROHs, the presence of one SNP with a missing genotype and no more than one possible heterozygous genotype was allowed for ROHs longer than 8 Mb [77]. For the genomic regions established in the ROH analyses, genes that were fully or partially localized within the detected genomic regions were selected. Gene identification was performed using the chicken genome assembly GRCg6a (GCA_000002315.5; [78]) in the Ensembl genome database [79]. The genes identified in this way were defined as candidate genes. The linkage disequilibrium (LD) decay was evaluated using the PLINK program, and the resultant data were visualized using the ggplot2 package (version 4.0.1) in R [80,81].

## 3. Results

### 3.1. Phenotypic Characteristics of Dwarf Breeds

**Figure 2:**
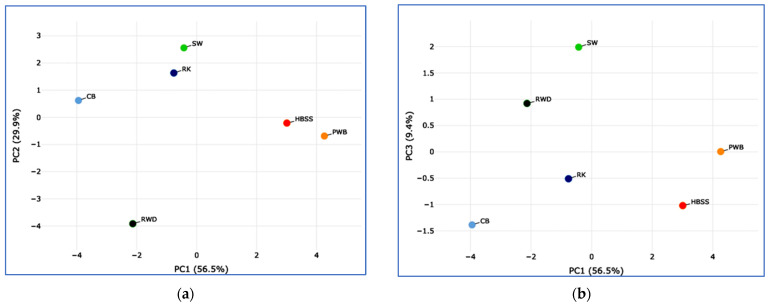

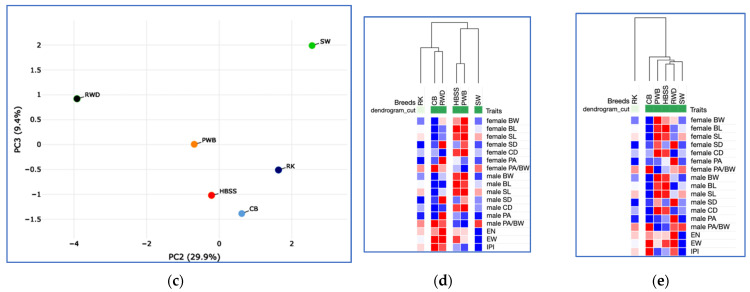
Principal component analysis (PCA) plots and hierarchical clustering (HC) dendrograms constructed using all combined quantitative phenotypic characteristics in the chicken breeds studied and illustrating the relationships among them. PCA plots showing (**a**) principal component 1 (PC1) and principal component 2 (PC2), explaining 56.5% and 29.9% of the total variance, respectively; (**b**) PC1 and principal component 3 (PC3), explaining 56.5% and 9.4% of the total variance, respectively; and (**c**) PC2 and PC3, explaining 29.9% and 9.4% of the total variance, respectively. HC dendrograms based on (**d**) the Euclidean distance metric and (**e**) the matrix values for a precomputed distance matrix. Breeds: RK, Russian Korolyok; CB, Cochin Bantam; HBSS, Hamburg Bantam Silver Spangled; PWB, Polish White-crested Black; RWD, Red White-tailed Dwarf; SW, Silkie White.

The phenotypic parameters of the examined bantam/dwarf chicken breeds are presented in Table 1. CB had the lowest BW of females and males (828 and 1163 g, respectively; *p* < 0.01), while PWB had the highest BW (1334 and 1836 g, respectively; *p* < 0.01). CB was significantly inferior to most other breeds in such body measurements as BL and SL (*p* < 0.01), while PWB was superior in SL and CD (*p* < 0.05). PWB and RWD had the highest SD values (*p* < 0.05). RWD demonstrated the highest PA values, while RK had the lowest (*p* < 0.05). The PA/BW index ranged from 50.90 in PWB to 81.52 in CB. The EN index varied between 81.5 in SW and 162.5 in RWD, EW between 39.0 g in SW and 57.5 g in CB and RWD, and the IPI coefficient between 3.70 in CB and 10.06 in SW. The phenotypic (exterior and performance) traits in RK generally conformed to the median values or were close to the minimum values among all the examined breeds. To present the overall pattern of interbreed differences in phenotypic characteristics, we generated PCA plots and HC dendrograms taking into account all quantitative traits (Figure 2).

PCA plots (Figure 2a–c) show that, due to the high variability of the studied traits, the breeds exhibited a certain scatter in their relative positions. A certain similarity in phenotypic characteristics could be observed between the European PWB and HBSS breeds, which is also reflected in the HC dendrogram (Figure 2d). RK formed a single, isolated cluster for both HC modifications (Figure 2d,e).

### 3.2. Genetic Diversity of Dwarf Breeds

Genetic diversity analysis of the studied chicken breeds based on their whole-genome SNP genotypes revealed that RWD (*p* < 0.001) and, to a lesser extent, PWB were characterized by higher *H_O_* and *H_E_* values, as well as *A_R_* values. HBSS (*p* < 0.001) and RK, although to a lesser extent, had the lowest heterozygosity and diversity indices (Table 2). *F*_IS_ values were highest in CB (0.116; *p* < 0.001), indicating the presence of probable inbreeding in this breed, which was not the case for other breeds.

### 3.3. Genetic Divergence and Phylogeny of Dwarf Breeds

**Figure 3:**
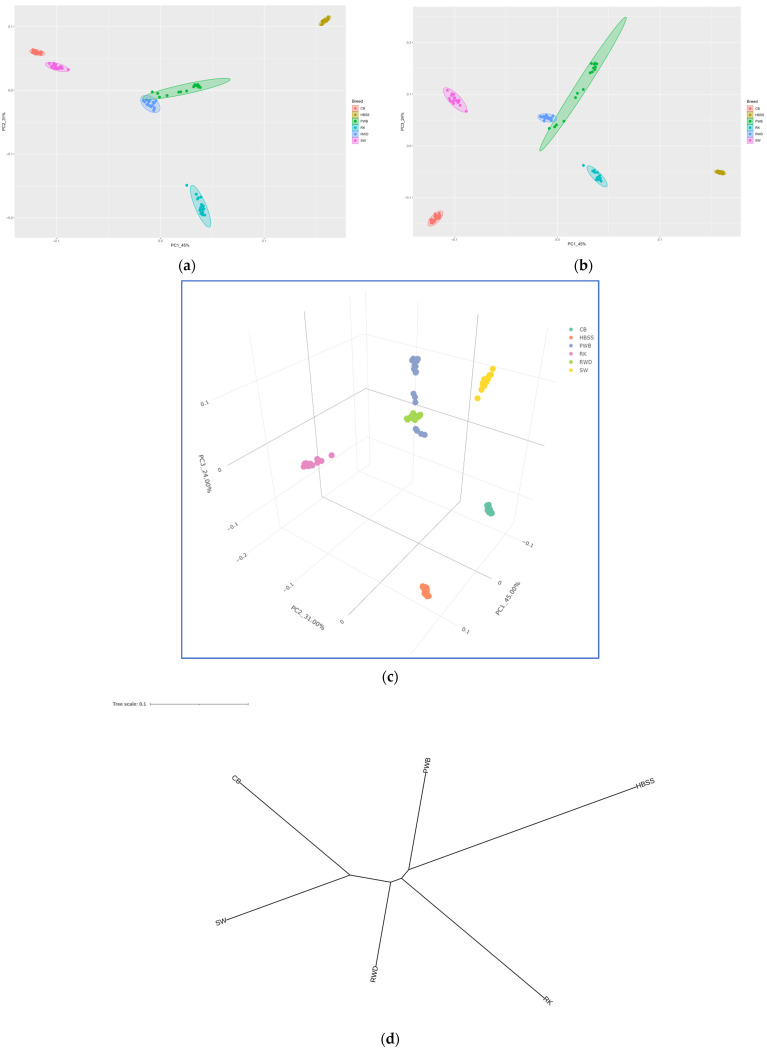
Principal component analysis (PCA)-based genome-wide assessment of genetic divergence of the Russian Korolyok from other dwarf breeds. PCA plots showing (**a**) principal component 1 (PC1) and principal component 2 (PC2); (**b**) PC1 and principal component 3 (PC3); and (**c**) PC1, PC2 and PC3 on a 3D plot, explaining 45%, 31% and 24% of the total variance, respectively (in all three plots). (**d**) Neighbor-Joining (NJ) dendrogram of the studied breeds constructed on the basis of genetic distances. NJ dendrogram formula (in Newick tree format): ((((CB:0.14660464,SW:0.13439895):0.04209001,RWD:0.08692061):0.0117496,RK:0.19002771):0.01143783,HBSS:0.24736202,PWB:0.09995273). Breeds: RK, Russian Korolyok; CB, Cochin Bantam; HBSS, Hamburg Bantam Silver Spangled; PWB, Polish White-crested Black; RWD, Red White-tailed Dwarf; SW, Silkie White.

PCA analysis of the SNP genotype distribution revealed that the RK population was significantly distant from other dwarf chicken breeds (Figure 3). Notably, RK was somewhat closer to European dwarf breeds, such as RWD and PWB, that were significantly related to each other. The other three breeds, i.e., CB, HBSS and SW, diverged significantly from each other and from RK, PWB and RWD. Overall, individuals within the breeds (except WD) showed high genetic affinity, suggesting an essential consolidation of the studied populations.

Analysis of the phylogenetic dendrogram (Figure 3d) revealed that a trifurcation point in the center of the tree identified three branches: Asian (CB and SW), European (which includes RK, although this is located basally with respect to the two true European breeds), and commercial synthetic (with one RWD breed). Thus, the studied breeds can be divided into the following main phylogenetic groups: Asian and European, as well as commercial synthetic, which occupied an intermediate position, although it was located closer to the European dwarf breeds. The European branch, in turn, had a separate offshoot, which included HBSS and PWB.

**Figure 4:**
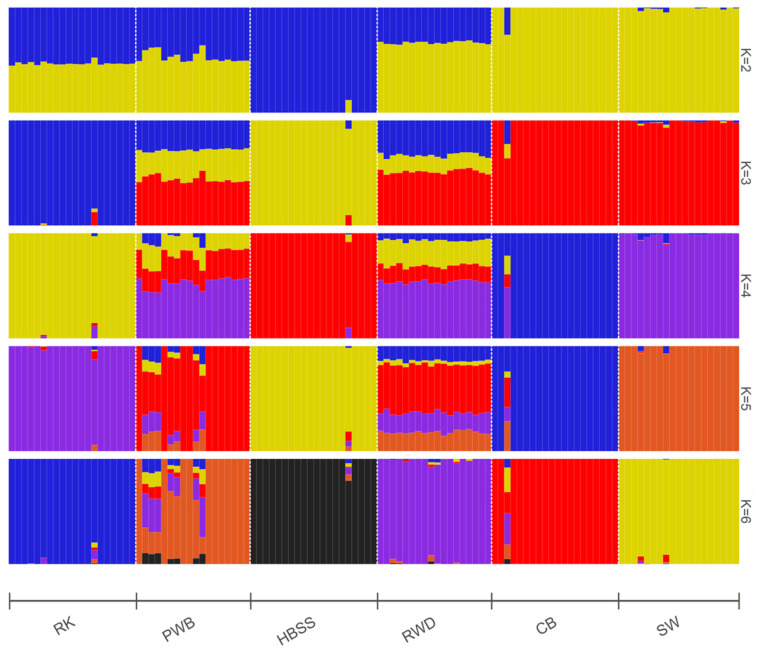
Bar plots of the genetic structure of the studied chicken populations based on the admixture analysis with an increase in the number of ancestral populations (K) from 2 to 6. Individual ancestry proportions inferred with admixture are shown in different colors. Breeds: RK, Russian Korolyok; CB, Cochin Bantam; HBSS, Hamburg Bantam Silver Spangled; PWB, Polish White-crested Black; RWD, Red White-tailed Dwarf; SW, Silkie White.

Analysis of admixture patterns on bar plots in Figure 4 revealed that, at K = 2, HBSS represented a distinct cluster. At K = 3, RK also formed a single cluster. The last to separate, at K = 4, were the SW and CB breeds. Complete separation of all six breeds occurred at K = 6, which corresponded to the optimal number of ancestral populations (according to the CV error plot, Supplementary Figure S2). The synthetic nature of RWD was clearly visible in the resultant admixture plot at K = 2 to 5, and PWB exhibited it at K = 2 to 6. At K = 6, the studied populations (except PWB) demonstrated an essential degree of consolidation.

### 3.4. Demographic History of the Studied Dwarf Breeds

**Figure 5:**
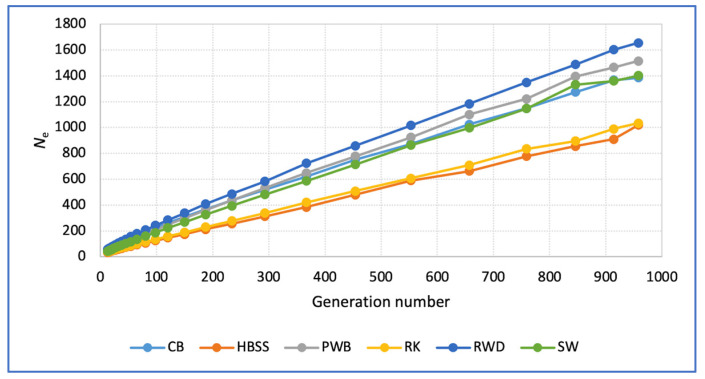
Graph of the effective population size (*N*_e_) of the studied chicken breeds. Breeds: RK, Russian Korolyok; CB, Cochin Bantam; HBSS, Hamburg Bantam Silver Spangled; PWB, Polish White-crested Black; RWD, Red White-tailed Dwarf; SW, Silkie White.

When examining the demographics of the surveyed populations in a historical context, which was inferred from their SNP genotypes, we can see (Figure 5) that at the level of 914–958 generations ago, or 420–440 years ago, the minimum *N*_e_ value of the ancestral populations was found in HBSS (909–1019) and RK (989–1034). The maximum values were found in the ancestral RWD population (*N*_e_ = 1602–1654). Subsequently, a general trend of decreasing *N*_e_ was observed in all populations, and they converged in this indicator. More modern populations (13 generations ago, or about 10 years ago) have low *N*_e_ levels: HBSS, 34; RK, 38; PWB, 42; SW, 42; CB, 49; and RWD, 57.

A more in-depth analysis of the demographic history and *N*_e_ changes was performed by examining LD decay on chromosomes GGA1–GGA28 (Supplementary Figure S3). To enhance the LD decay analysis accuracy, GGA16 and GGA25 were excluded from further consideration. Breeds with greater *N*_e_ values, such as PWB, RWD, CB and SW, had smaller distances between SNPs with high LD values. HBSS was distinguished by higher LD values and distances between SNPs (over 800 Kb) exhibiting LD. Based on the LD decay analysis plots and breed-specific patterns, we identified four groups of breeds as follows.

The first group only involved one breed, HBSS, which had the slowest LD decay. This breed exhibited a very high and flat LD curve on several chromosomes, with an extremely slow decline and an initial *r*^2^ value of >0.8. In some cases, *r*^2^ remained within 0.5–0.7 even at a distance of 1000 Kb between SNPs. Based on a visual assessment of the plots, it can be concluded that this breed has historically experienced a genetic bottleneck or has been subject to more intense inbreeding and/or selection.

In the second group, a single breed, RK, with a slower LD decay, was identified. The breed’s plot curves were similar across chromosomes and closely resembled the HBSS distribution. With an initial value of ~0.7–0.8, *r*^2^ subsequently declined over a 1000 Kb SNP distance to ~0.3–0.5. RK can be considered an inbred breed with a lower *N*_e_ value. However, it should be noted that the inbreeding level in this breed has recently decreased, confirming the effectiveness of ongoing breeding efforts.

The third group included three breeds, i.e., PWB, SW and CD, that had medium *N*_e_ values and showed a moderate LD decay. Their plot curves began at *r*^2^ of ~0.6–0.7. Then, a smooth decline to *r*^2^ = 0.2 at a distance of approximately 250–400 Kb between SNPs was observed, with the plots being rather consistent across all chromosomes. These breeds seem to be considered established and consolidated, with a selection history typical of domestic animals.

Finally, the fourth group also included one RWD breed that exhibited relatively fast LD decay. The curves began significantly lower (with an initial *r*^2^ of ~0.4–0.6) and declined more rapidly. An *r*^2^ level of 0.2 was reached at SNP distances of ~100–200 Kb. This breed had the highest *N*_e_ and genetic diversity values among all six breeds studied.

### 3.5. Homozygous Regions and Candidate Genes Under Selection Pressure

In this study, we also obtained data on the frequency of homozygous regions in the RK genome and other compared breeds. The primary objective was to examine the impact of selective breeding for specific phenotypic traits on the development of the dwarf breeds’ genome. A genome-wide search identified 22 homozygous regions with a frequency of over 75%, including three with a frequency of over 90% of individuals on chromosomes GGA2, GGA4 and GGA7 (Table 3). In our study, we focused specifically on these three regions and considered the genes located within them as prioritized candidate genes (PCGs), of which we identified 20. For example, a homozygous region occurring in 100% of individuals was identified on GGA2 in the region 80,850,520–83,898,189, where five PCGs were annotated: *GRB10* (growth factor receptor bound protein 10)*, RPRD1A* (regulation of nuclear pre-mRNA domain containing 1A), *GALNT1* (polypeptide N-acetylgalactosaminyltransferase 1), *VSTM2A* (V-set and transmembrane domain containing 2A) and *FHOD3* (formin homology 2 domain containing 3).

Homozygous regions were additionally examined in all six breeds to find regions that were co-present and identify candidate genes located within the shared ROHs. These genes can also be considered candidates for the manifestation of the dwarf phenotype. One such shared homozygous region was found in RK, i.e., 16,202,403–17,300,743 on GGA10. It overlapped with a similar region in HBSS (16,202,403–18,012,334). Correspondingly, five more genes that can be considered PCGs were identified (Table 3): *NR2F2* (nuclear receptor subfamily 2 group F member 2), *ARRDC4* (arrestin domain containing 4), *FAM169B* (family with sequence similarity 169 member B), *IGF1R* (insulin like growth factor 1 receptor) and *SYNM* (synemin). There were no ROHs shared with the other breeds.

To verify the ROH analysis accuracy in all homozygous regions (Table 3), we also calculated the density of detected SNPs (Supplementary Table S1). This density appeared satisfactory across all homozygous loci, ranging from 21.49 to 73.79 SNPs per 1 Mb. Homozygous regions with a frequency greater than 90% were not short in terms of ROH length, exceeding 500 Kb.

## 4. Discussion

Genetic monitoring of various chicken breeds and populations makes it possible to track the dynamics of population gene pools, study the place of certain local breeds in the global gene pool, and take a deeper look at the problems of the origin of the domestic fowl and breed formation [44,82,83,84,85,86]. In Russia, RK are currently recognized as dwarf ornamental poultry, tracing their origins back to ancient times from local chickens and European fowls (of the Central European group, according to Ivanov [22,23]). In this work, we evaluated their phenotypes and genome-wide SNP-based genotypes in comparison with some other dwarf (bantam) breeds of European and Asian origin.

### 4.1. Phenotypic Comparison of Dwarf Breeds

Specific phenotypic peculiarities define a breed’s “portrait,” and the characterization of variable quantitative traits forms the basis for breed selection and improvement [87,88,89,90]. Association studies, including GWASs and selective sweep scans, are presently used to identify associated markers and candidate genes for important phenotypic traits [91,92,93,94]. One of the most significant metrics for measuring animal/bird health is BW [57,58]. It is a fundamental concept for such important body elements as the strength of the supporting apparatus (skeleton), the need for dynamic and static muscle tension for movement, and the ability to maintain balance, nutritional needs, etc. In our study, HBSS and RWD had the highest BW, while CB had the lowest, indicating the highest body and skeletal development in the former and the lowest in the latter among the dwarf populations examined.

Objectively, meat body shape can be determined using basic measurements, including BL, CD and PA [57]. BL characterizes a bird’s size and the development of its internal organs; PWB and HBSS were superior in this trait, whereas CB was inferior to all other breeds. CD also characterizes a bird’s size and the development of internal organs and muscles; insufficient CD may characterize a narrow-bodied type [57,58]. PWB and RWD were characterized by higher CD values, while true bantams (RK, CB and SW) had lower CD values. PA characterizes the level of development of the pectoral muscles and, therefore, can serve as an indicator of carcass quality [57]. Unsurprisingly, the RWD broiler line demonstrated superior performance in this trait, whereas true bantams (RK, CB and SW) performed inferiorly to other breeds. Skeletal development measurements (e.g., SL, SD, etc.) are useful additional indicators of roosters’ and hens’ size [57]. In particular, SL characterizes body conformation and largely determines a bird’s body height. PWB and HBSS exhibited the highest values for this trait, while CB had the lowest. SD can be a good indicator of overall skeletal development. PWB and RWD had higher SD values, whereas RK demonstrated lower values. Similar difference trends can be found for other phenotypic traits (PA/BW, EN, EW, and IPI). These major phenotypic differences between the six studied breeds were mainly supported by the inferred PCA plots and HC dendrograms (Figure 2).

### 4.2. Phylogeny and Genomic Diversity Insights

According to Moiseyeva [45], small bantam chickens, common throughout the world, are believed to have a direct origin from the main ancestor of the domestic fowl, i.e., the wild species *G. gallus*, or from egg-laying hens of Mediterranean origin. Crossbreeding bantams with local chickens in Russia produced RK [45]. This motivates an interest in further exploring the phylogenetic relationships of this breed, which we attempted to address in this study.

As shown by genome-wide PCA (Figure 3a–c), NJ-assisted phylogeny (Figure 3d), and admixture (Figure 4) analyses, RK occupies a basal position in its phylogenetic relationships relative to dwarf (small) breeds of European origin. It should be noted, however, that HBSS is significantly genetically distinct, being essentially a bantam variety of the very ancient European Hamburg breed. For example, Pallas [21,25] was very familiar with the original Hamburg breed and observed it in Russia as early as the second half of the 18th century. In this regard, he also referred to the description of this breed in 1738 by the English naturalist Eleazar Albin (fl. 1690–c. 1742) as “*G. hamburgensis*” (“Albin. av. III. Table 32”; [10]) and in the 1763 book of the German ornithologist Johann L. Frisch (1666–1743) (“Frisch. av. Table 127. 128”; [95]). Albin [10] reported that these chickens were brought to the kingdom by English merchants from the German city of Hamburg, which was reflected in the subsequent name of the breed during its further improvement in England [22,23]. Darwin [42] specified that the spangled Hamburgh sub-breed is of English origin, while the penciled Hamburgh is of Dutch origin, with both sub-breeds including gold and silver varieties.

The population of another European breed, PWB, in our study was lower in BW than standard-sized birds of this breed. Specifically, standard PWB roosters and hens weigh 2.75 and 2 kg, respectively, while in the RRIFAGB population, the BW of roosters and hens was 1.8 and 1.3 kg, respectively, virtually identical to that of HBSS (Table 1). This circumstance allowed us to include the local PWB population in this study, given its long-standing European origin. This breed has an ancient history and was depicted by the Dutch painter Jan Monckhorst (1636–1695) as early as 1657. It was well known to Darwin [42], who described it under the name *Crested* or *Polish*, also citing its earlier mentions by the French scholar Pierre Borel (c. 1620–1671) [96] and Albin [10]. Pallas [21] left an early description, apparently of this breed in Russia, as follows: “*A rare form, close to it* [i.e., to Russian chickens of the Pavlov type], *called by English ornithologists the Hamburg, is all black, with a large white feather crest, without a* “*beard*” *and with bare legs; in my opinion, this is the most beautiful of all artificially bred breeds.*” The designation of the breed as Hamburg could have been a slip of the tongue by Pallas or may actually have appeared in early English sources, implying the importation of Dutch crested chickens (i.e., PWB) to England via Hamburg, similar to the true Hamburg breed. At the same time, Ivanov [22,23] reported that a variety of Polish chickens, i.e., black with a white crest (without a beard), are called Dutch. The possible hybrid origin of PWB from chickens from the Netherlands and Poland [52] can be supported by the corresponding admixture pattern of this breed (Figure 4).

Two old breeds of Chinese roots, CB and SW, predictably showed a common Asian origin at the genomic level. Darwin [42] described both breeds, calling them the *CB sub-breed* and *Silk*, the latter being an ancient breed. Silkie-like fowls are mentioned in early Chinese sources: the *Book of Jin* (648), referring to the time of the Jin dynasty (266–420), a poem by Du Fu (712–770); *Taiping Yulan* (977–983); etc., as compiled by the Italian scholar Elio Corti (1942–2017) [45]. Claims by some authors and sources (e.g., [97]) that this breed was known to Aristotle in the 4th century BC appear to be erroneous [45]. The Italian explorer Marco Polo (1254–1324) observed Silkie-like chickens in China in 1275–1291 [45,98], through which they became known in Europe and were mentioned in later works, e.g., by the Swiss scientist Conrad Gessner (1516–1565) [99], the Italian naturalist Ulisse Aldrovandi (1522–1605) [100] and others (as reviewed by Corti [45]). Pallas [21] saw this breed in Russia and reported that it was imported into the province of Astrakhan from Persia, into Siberia from China, and into St. Petersburg from England. The commercial RWD strain represents parent stock birds from the sire line of the dam parental stock of a French broiler cross and was further developed as a B77 line at the All-Russian Poultry Research and Technological Institute in Sergiev Posad. RWD was originally produced, according to some sources, in England in the second half of the 20th century from crossing two American common breeds, the Plymouth Rock White, New Hampshire, one extinct English breed, the White Surrey, and, probably, birds carrying the dwarfism gene (alleles), each of which, in turn, is a cross between birds of European and Asian origin [51,52,101,102]. This determined the intermediate position of RWD relative to the two main evolutionary branches of chicken breeds, i.e., European and Asian, as well as its poorly consolidated population structure (Figure 3) and synthetic admixture pattern (Figure 4). In contrast, there were higher genetic affinity of individuals and more consolidated population structures within other breeds, including RK, which confirms the previous findings for this old Russian breed [44,51].

Thus, the obtained phylogenetic data for RK and five other dwarf (small) breeds are in line with the global chicken phylogeny presented in our previous large-scale genome-wide SNP-assisted study on the rugged (and ragged) landscape of ancestry and demographic history in numerous chicken breeds [51]. Also, our genome-wide SNP genotyping is fully consistent with historical data on the studied breeds, demonstrating their genetic distinctiveness, on the one hand, and common ancestry in accordance with the existing classification systems, on the other [12,22,23,48,49,51,52,103]. For example, Ivanov [22,23] classified the Hamburg and Polish breeds as egg-laying chickens, and within these, as part of a general Central European group. He also included common Russian chickens (Ushanka, Gilian, Orloff and Pavlov) in this group, and it is not surprising that, according to our findings, RK should also be included here. Breeds such as the Plymouth Rock were classified by Ivanov [22,23] as part of an American group of a different, dual-purpose type; these are the ancestors of the studied RWD, which occupies an intermediate position between European and Asian breeds. Our data and the topology of the phylogenetic tree we obtained fit into these early and recent classification systems and surveys of chicken breeds.

The synthetic nature of RWD was also reflected in its highest genetic diversity among the chicken breeds studied, based on their whole-genome SNP genotypes. HBSS and RK were characterized by minimal diversity. These data are also concordant with a previous SNP-based study by Dementieva et al. [51], who measured LD in a significant sample of the worldwide chicken breeds from Europe, Asia, North America and Oceania. LD values were higher in HBSS and RK. The presence of a large number of SNP markers manifesting LD can be explained by the limited size of the populations studied or a small number of efficiently mating sires within a population. At the same time, the examined breeds (except CB) did not exhibit any degree of genomic inbreeding.

In our study, using admixture analysis (Figure 5), RK demonstrated shared affinity with both European-derived (HBSS, PWB and RWD) and Asian-derived (CB and SW) breeds at K = 2. However, at K = 3 and K = 4, RK lost its similarity to HBSS, sharing some components with PWB and RWD. At K = 5, RK showed similarity only with RWD; the purple RWD segment shared with RK accounts for approximately 20% of the RWD genome. This may be due to the presence of a common ancestral genomic component in RK and RWD, perhaps due to the use of a common breed (or related breeds) in the past when creating RK and RWD.

### 4.3. Demographic History Insights

Modern dwarf chicken populations worldwide are often bred in small groups and isolated from the exchange of genetic material. This may lead to reduced variability that could serve as a source for identifying genomic features important for defining the demographic history of populations.

According to the demographic history analysis using the *N*_e_ index (Figure 5), the six dwarf breeds showed a general trend towards reducing *N*_e_ values, with HBSS and RK being inferior to the other four. The RK population, despite the observed *N*_e_ trends (Figure 5), is characterized by a higher *H_O_* relative to *H_E_*. This is achieved by obtaining a large number of young animals to select more viable individuals through selective breeding and zootechnical evaluation, which helps to reduce the impact of inbreeding depression. To increase genetic diversity, we deliberately alter the sex ratio in the current population, assigning no more than 4 hens to a single rooster, instead of the physiologically reasonable 8–10 hens. Roosters are matched to hens based on the origin of the individuals. Thus, the potentially undesirable biological and conservation consequences of the inferred *N*_e_ trend for the RK breed are mitigated by the modern breeding practices used, reducing the risks to the preservation of the breed.

An important aspect of elucidating the demographic history of populations is the analysis of LD decay, which occurs as a result of recombination and the weakening of genetic linkage between closely spaced SNPs over generations [104,105]. Reflecting population history, population size and selection pressure, it reveals how long stretches of ancestral genomes are fragmented into shorter haplotypes [106]. Investigating LD decay helps to understand genetic diversity and population evolution by identifying regions where linkage has been preserved for the longest time. The more generations that have passed since linkage arose, the more rapidly it decays. LD decay occurs more quickly in large populations than in small ones (where linkage can be preserved longer due to genetic drift). This allows one to reconstruct population expansion or contraction. Regions with preserved LD may contain, for example, important regulatory elements. By assessing genetic diversity, it is possible to determine how many “old” and “new” haplotypes are present in a population [107].

The results of the LD decay analysis obtained in the present study (Supplementary Figure S3) align with previous findings on LD changes in local populations depending on the distance between markers (e.g., [108,109]). The highest LD values were observed in the old and small HBSS population. In this population, we observed the slowest LD decay due to continuous inbreeding over many generations. The RK population is also inbred with low *N*_e_ and is characterized by high LD values and slow LD decay. However, it should be noted that recent inbreeding in RK has decreased, confirming the effectiveness of current breeding efforts. HBSS and RK sometimes demonstrated slightly more “uneven” LD curves, which may be due to differences in selection intensity on different chromosomes. All plots showed LD decay at inter-SNP distances of 1000 kb or less. LD decay is known to occur more slowly in young, isolated, or selectively managed populations [106]. Long LD (low-decay) regions indicate recent selection or past population sparseness. It is likely that the nature of LD decay in HBSS and RK corresponded precisely to the isolation of these populations and their small numbers in the past.

Restoring genetic diversity in populations that are few in number is of great importance for the conservation of rare breeds. Programs based on LD analysis for breeding small populations are being developed [110]. Previously, we examined in detail comparative changes in LD in the Russian White breed, i.e., in a population that went through a “bottleneck” vs. another population with restored genetic diversity [111]. The modern population of Russian White chickens was characterized by the decay of long LD regions and a reduction in the frequency of haplotypes of the ancestral population. In the studies of Gao et al. [112], the *r*^2^ values in local Asian chickens decreased faster, while European breeds demonstrated the slowest rate of LD decline, similar to what we observed for the Asian CB and SW breeds and the European HBSS breed, respectively.

Our investigation of the PWB, RWD, CB and SW breeds demonstrated higher homogeneity of LD decay plots across all chromosomes (Supplementary Figure S3), including smaller distances between SNPs at high LD values. This is indicative of high-quality SNP data and the current absence of selection pressure in the populations we examined. LD typically decays faster in older populations (such as PWB, CB and SW) and/or populations with greater *N*_e_ (PWB, RWD, CB and SW), where recombination has had time to break linkages [105]. Rapid decay means that *r*^2^ drops to low values (e.g., <0.2) over short distances. In the younger RWD breed, the LD decay curve reached a plateau (i.e., background LD level) earlier than in the others. This means that intermarker distances of >500 kb for this breed carry almost no linkage information. Other studies [113,114] have shown that a faster decline in LD (as in the case of PWB, CB, SW and RWD) suggests less directional selection and higher genetic diversity in a population.

### 4.4. Candidate Genes: Prime PCGs on GGA10

One effective tool for exploring selective sweeps and genomic inbreeding is analysis of ROHs that are continuous homozygous segments of DNA passed on to offspring from parents sharing a common ancestor. Accordingly, this study allowed us not only to assess the degree of inbreeding in six dwarf breeds but also, by characterizing the extent of ROH islands, to identify candidate genes within them that may be under selection pressure (Table 3).

Based on the genome scanning results for dwarf chicken breeds, five genes (*NR2F2*, *ARRDC4*, *FAM169B*, *SYNM* and *IGF1R*) were of primary interest. These genes were annotated in a common homozygous region in RK and HBSS. We will briefly discuss them below as prime PCGs.

*NR2F2*: upregulated in broilers [115]; an inhibitor of fat deposition in birds, it affects a number of potential regulators associated with adipogenesis [116,117,118]. In our case, the homozygous region in this gene may be associated with a specific fat metabolism in RK and other bantams, since they do not accumulate visceral fat, unlike standard breeds.

*ARRDC4*: involved in glucose metabolism in mammals. Systemic and molecular gluconeogenesis and glucagon responses are compromised in mice with a total loss of the ARRDC4 protein [119].

*FAM169B*: a new gene of protein family 169. The functions of this protein are unclear.

*SYNM*: an intermediate filament type VI protein, it plays an important role in the muscle cell cytoskeleton. Cytoskeletal proteins provide resistance to mechanical stress. In Qinchuan cattle, mutations in the *SYNM* gene are associated with skeletal muscle hypertrophy [120].

*IGF1R*: has tyrosine kinase activity; putatively related to skeletal muscle development and growth, as shown in beef cattle [121,122], and growth retardation in mice [123,124]. Evolution of the insulin-like growth factor system has played a role in diversification during chicken breeding [125].

### 4.5. Candidate Genes: Other PCGs

#### 4.5.1. GGA2

*GRB10*: produces a protein that binds to growth factor receptors and interacts with both insulin and insulin-like growth factor receptors. Overexpression of certain isoforms of the encoded protein suppresses tyrosine kinase activity and leads to growth inhibition via negative modulation of the *IGF1R* cascades [123,124]. RK is a dwarf breed, which may explain the genomic footprints of selective breeding aimed at reducing body size during the breed’s development. Using analysis of population structure, ROH islands, and selection signatures, the *GRB10* gene was shown to be associated with body size, growth and development in chickens [1]. This provides a theoretical basis for the subsequent development of molecular markers and analysis of the genetic mechanism underlying body size in chickens.

*RPRD1A*: another gene identified in the homozygous region in RK encodes a protein that regulates the cell cycle and transcription of genes in the Wnt/β-catenin pathway. This pathway determines the regulation of embryogenesis, morphogenesis, tissue renewal and regeneration, proliferation, and cell differentiation [126]. It is possible that it also influences the development of the dwarf phenotype in RK and other dwarf fowls.

*GALNT1*: one of the important genes located at a homozygous locus, it catalyzes the initial reaction in the biosynthesis of O-linked oligosaccharides and the transfer of an N-acetyl-D-galactosamine residue to a serine or threonine residue on a protein receptor. Polymorphism in the *GALNT1* gene can affect ovarian development and function in chickens [127]. A specific haplotype in this gene is a possible cause of decreased reproductive function in the RK population.

Two other PCGs in the same region of GGA2 (*VSTM2A* and *FHOD3*) may also be of interest due to their functional importance. *VSTM2A* is one of the candidate genes under selection during chicken domestication and subsequent breed formation [128,129,130], and *FHOD3* gene expression is associated with functional processes in the heart and in muscle development in general [121,131].

#### 4.5.2. GGA4

The homozygous region located on GGA4 (8,274,461–8,965,874) occurred with a frequency of 94% in the RK population. PCGs annotated in this region, i.e., *DACH2* (dachshund family transcription factor 2), *POF1B* (POF1B actin binding protein), *CHM* (CHM Rab escort protein), *APOOL* (apolipoprotein O-like), and *HDX* (highly divergent homeobox), are possibly associated with embryonic development and reproduction in poultry.

*DACH2*: encodes a protein that may be involved in the regulation of organogenesis and myogenesis [132] and may also play a role in ovarian development, fertility/fecundity, premature ovarian failure and the development of female primary sexual characteristics [133,134,135,136].

*POF1B*: may be involved in the etiology of premature ovarian failure and ovarian development and fertility in general [134,137].

*CHM*: encodes component A of the RAB holoenzyme geranylgeranyltransferase. Mutations in this gene in humans can cause a rare form of X-linked chorioretinal dystrophy, i.e., choroideremia [138].

*APOOL*: encodes a protein containing a domain of the apolipoprotein O superfamily. This domain is found in proteins in circulating lipoprotein complexes. Overexpression of *APOOL* leads to mitochondrial fragmentation, a decrease in the basal oxygen consumption rate, and altered morphology of cristae (the ridge-shaped inner folded membranes of mitochondria). Downregulation of *APOOL* impairs mitochondrial respiration and causes severe changes in crista morphology [139].

*HDX*: provides DNA-binding transcription factor activity, RNA polymerase II-specific DNA-binding activity, and RNA polymerase II cis-regulatory region-specific DNA-binding activity. In humans, mutations in this gene are associated with premature ovarian failure [140].

Overall, this homozygous locus may contribute to decreased reproductive capacity in RK and requires further study.

#### 4.5.3. GGA7

The homozygous locus on GGA7 in the region 26,318,341–27,858,543 embraces the following PCGs: *EAF2* (ELL associated factor 2), *SLC15A2* (solute carrier family 15 member 2), *SEMA5B* (semaphorin 5B), *PDIA5* (protein disulfide isomerase family A member 5), *SEC22A* (SEC22 homolog A, vesicle trafficking protein), *ADCY5* (adenylate cyclase 5), *HACD2* (3-hydroxyacyl-CoA dehydratase 2), *MYLK* (myosin light chain kinase) and *KALRN* (kalirin RhoGEF kinase) associated with the reproductive function in animals and humans. The genes in this ROH were also shown to be candidates for the growth and development of the body in crossbred chickens [141].

*EAF2*: mediates transcriptional elongation regulator activity. It has been linked to several other malignancies and multiple physiological processes, such as transcription, apoptosis, embryogenesis, and DNA repair [142]. In chickens, it may be a candidate gene associated with growth and meat productivity [141].

*SLC15A2*: a member of the SLC gene family and may regulate microRNA expression in the chicken oviduct [143] and be involved in growth and meat performance [141].

*SEMA5B*: found at a homozygous locus, possibly as a significant gene related to growth and meat productivity in chickens [141]. It also appears to be responsible for growth and development in the Chinese Zhongshan duck breed that is characterized by a small body size, high resistance to diseases, tender meat and a small amount of subcutaneous fat [144].

*PDIA5*: in addition to growth and meat productivity in chickens [141], it may also be associated with a decline in reproductive performance in the RK population. This gene encodes a member of the disulfide isomerase family of endoplasmic reticulum proteins that catalyze protein folding and thiol–disulfide exchange reactions. It is one of the key genes associated with pathology in human granulosa cells (primary ovarian failure) [145]. It is involved in pathways during osteoclast differentiation and cytokine interactions. It is associated with the development of osteoporosis in humans [146].

*SEC22A*: another gene potentially associated with growth, meat performance [141] and reproduction. The protein encoded by this gene belongs to the SEC22 family of vesicular transport proteins. It has been identified as a putative biomarker of oocyte maturation in vitro in cattle [147].

*ADCY5*: encodes part of the membrane-bound enzyme adenylate cyclase. It may also be associated with reproduction, as other studies have identified it as a candidate gene for the egg-laying trait in Muscovy ducks [148].

*HACD2*: associated with growth and meat productivity in crossbred chickens [141] and in fatty acid metabolism in broilers [149,150]. The protein encoded by this gene can catalyze the third step (dehydration) in the conversion of long-chain fatty acids to very long-chain fatty acids.

*MYLK*: may influence growth and meat performance [141] and egg-laying-related traits in RK and other dwarfs, as it is a significant candidate gene for the development of the broodiness instinct in chickens [151]. This gene, which is a muscle member of the immunoglobulin gene superfamily, produces a calcium/calmodulin-dependent enzyme (myosin light chain kinase). This kinase phosphorylates the regulatory light chains of myosin to facilitate the interaction of myosin with actin filaments to generate contractile activity.

*KALRN*: possibly associated with growth and meat productivity in chickens [141] and with reproduction in cattle [152], the gene encodes a protein that interacts with huntingtin associated protein 1 (HAP1). HAP1 is a huntingtin-binding protein that can function in vesicular trafficking and promote the exchange of GDP (guanosine diphosphate) for GTP (guanosine triphosphate).

### 4.6. Candidate Genes: General Insights

Altogether, we can classify the candidate genes we found in the ROH islands specific to RK and other compared breeds (Table 3) into several groups according to their functional purposes.

One of these groups embraces genes (*SIM1*, *NANOG*, *GATA3*, *ASCL1*, *PAX3*, *PHC1*, *ING4*, *NR2F2*, *ZNF384*, *ATN1*, *DACH1*, *DACH2*, *GLI2*, *TFCP2L1* and *BCL6*) that are transcription factors and developmental regulators. They encode key regulatory proteins that control fundamental biological processes from embryonic development to the specialization of immune system cells.

Another group involves genes (*IL2RA*, *BECN1*, *AICDA*, *C1R*, *MASP1*, *CD86*, *CD4*, *PTPN6*, *C1S*, *C8B* and *IFI35*) associated with immunity. This set of genes encompasses both adaptive and innate immunity, ensuring the recognition and activation of a specific response.

A group of genes (*G6PC*, *ATP5C1*, *TXNRD1*, *TPI1*, *ENO2*, *IGF1*, *SLC2A14*, *PAH*, *GSTK1*, *ME3*, *TG*, *ST3GAL1*, *GALNT1*, *GPIHBP1*, *PREP*, *ADI1* and *PECR*) were also identified that encode enzymes, transporters and regulatory proteins, covering fundamental pathways of cellular metabolism, including ATP production, carbohydrate, lipid and amino acid metabolism, etc.

A group of genes encoding transport and membrane proteins (*PODXL*, *SLC41A2*, *NUP37*, *CHPT1*, *KPNA1*, *SCNN1A*, *SLC15A2*, *SLC35F5*, *VAMP1*, *PEX5*, *SLC25A36* and *SLC24A1*) provide fundamental cellular functions. They function in the plasma membrane, nuclear envelope, organelle membranes and vesicles, forming the basis for cellular homeostasis, intercellular communication and intracellular interactions.

Another group of genes (*MKLN1*, *LARGE1*, *TIMP3*, *ZYX*, *PICALM*, *PTK2*, *PCDH20*, *DLG2*, *CLASP1*, *ACTR3*, *ACTL6A* and *SYNM*) encode proteins that form the cell’s cytoskeleton and extracellular matrix. They control cell shape, division, movement, intracellular transport, and the transmission of mechanical and chemical signals, which underlies morphogenesis, immune responses and the maintenance of tissue architecture.

The ROH-based analysis also identified a group of genes (*CSTB*, *DRAM1*, *BECN1*, *USP5*, *CASP2*, *EED*, *USP45* and *USP13*) that encode key regulators of apoptosis and autophagy.

The diverse functions of these genes, from developmental regulation to metabolism and immunity, require further, more detailed study, as many are tissue-specific. For example, genes involved in metabolic and transcriptional processes are activated in the liver and brain, respectively. Therefore, our experimental data on genome structure and candidate genes in the dwarf breeds can serve as a source of information for further studies of transcriptomics in individual tissues and elucidate gene function in *G. gallus*.

Collectively, a number of discovered candidate genes (e.g., *GRB10*, *RPRD1A, APOOL, EAF2*, *SEMA5B*, *HACD2*, *IGF1R*, etc.) may play a significant role in the formation of dwarfism and physiological features of body build in the studied chicken breeds. One more PCG, *TMEM263* (transmembrane protein 263), identified on GGA1 in our study (Table 3) was previously attributed to the classical *ADW* gene due to a nonsense mutation in this gene [3]. The verification, clarification and elucidation of these genes’ involvement in forming specific phenotypic traits in fancy and commercial dwarf chickens will be anticipated as a subject of further investigations using, for instance, single-cell transcriptomic technologies [153,154].

Since our research used the Illumina 60K SNP array, it can be assumed that this could to some extent have limited the resolution of detection of ROHs and, accordingly, candidate genes under selection pressure. In this regard, it should be noted that in all ROHs (Table 3), the density of detected SNPs (Supplementary Table S1) was quite high (21–74 SNPs per 1 Mb), and the homozygous regions with an occurrence frequency of more than 90% were long (more than 0.5 Mb). Thus, the use of the Illumina 60K SNP chip apparently did not compromise too much the resolving accuracy of the ROH analysis and the identification of candidate genes. Other homozygous loci shown in Table 3 suggest the need for further studies using whole-genome sequencing. While being, in principle, positional candidates, the identified genes require more in-depth consideration in future studies. That is, these gene–phenotype associations require further experimental functional validation or GWASs to verify the suggestive links to specific phenotypes.

## 5. Conclusions

Our study assessed the phenotypic and genetic characteristics and diversity of the unique RK breed in comparison with five other dwarf breeds, all being rare chicken breeds. They exhibit non-ordinary ornamental and other phenotypic characters (small body size) and originated, as a rule, from a limited number of individuals. Methodological advantages in analyzing genome-wide genotyping results using SNP array technology made it possible to determine the genetic diversity of these rare dwarf breeds. Higher inbreeding values and lower heterozygosity in some of them may be due to temporal fluctuations in the size of these populations. A large proportion of homozygous regions (ROHs) in the genome of individuals was revealed in HBSS, although an increased ROH level was also observed in RK and CB. The identified patterns of phylogeny, admixture, and effective population size are consistent with historical data on the origins of these breeds. Twenty-six PCGs potentially associated with the dwarf phenotype, reproductive, and production traits of RK chickens and other dwarf breeds were identified in the regions of artificial selection signatures (ROHs). Specifically, such PCGs as *GRB10*, *RPRD1A*, *APOOL*, *EAF2*, *SEMA5B*, *HACD2*, *IGF1R*, *TMEM263* and others may be putatively associated with the development of dwarfism and physiological body conformation characteristics in the studied chickens. A number of genes potentially influencing reproductive and performance (egg production) traits in these populations were also established: *GALANT1*, *DACH2*, *CHM*, *POF1B*, *HDX*, *SLC15A2*, *PDIA5*, *SEC22*, *ADCY5*, *MYLK* and *KALRN*.

Small population size in these breeds, as well as a decline in their reproduction rates, may be indicative of a risk of possible inbreeding increase in future generations. These results expand our understanding of how selective breeding might have shaped the RK genome, as well as those of other dwarf breeds, and further contribute to their conservation.

## Figures and Tables

**Table 1:** The main phenotypic (including exterior) indicators (M ± SEM ^1^) of the Russian Korolyok and other bantam (dwarf) breeds at the age of 270 days.

	Breeds											
	RK		CB		HBSS		PWB		RWD		SW	
Origin ^2^	Russia; from crossing Bantams (Southeast Asia, Europe) and local fowls; a purebred strain (a mottled variety)		China; from the Cochin breed; a mottled variety		Holland, Germany, United Kingdom; from crossing local North Sea coast fowls and Ottoman Empire chickens; an inbred strain		The Netherlands, Poland(?); from crossing local fowls and Czubatka chickens from Poland; a purebred strain		England; from crossing Plymouth Rock White, New Hampshire and extinct White Surrey; a synthetic sire parent stock line of the dam strain (of an imported French commercial broiler cross)		China, Southeast Asia; from local fowls; a purebred strain	
Index	1		2		3		4		5		6	
Sex	♀	♂	♀	♂	♀	♂	♀	♂	♀	♂	♀	♂
*n*	30	10	41	13	24	4	27	9	14	5	30	14
BW	927 ± 16 ^↑^***^2,^ ^↓^***^3,4,5^	1289 ± 24 ^↑^***^2,^*^6,^ ^↓^***^3,4^	828 ± 17 ^↓^***^1,3,4,5,^ **^6^	1126 ± 24 ^↓^***^1,3,4,5^	1163 ± 31 ^↑^***^1,2,6,^ ^↓^***^4^	1757 ± 40 ^↑^***^1,2,5,6^	1334 ± 30 ^↑^***^1,2,3,5,6^	1836 ± 53 ^↑^***^1,2,5,6^	1094 ± 34 ^↑^***^1,2,6,^ ^↓^***^4^	1362 ± 50 ^↑^***^2,^**^6^ ^↓^***^3,4^	915 ± 21 ^↑^**^2,^ ^↓^***^3,4,5^	1191 ± 29 ^↓^*^1,^***^3,4,^ **^5^
BL	13.49 ± 0.08 ^↑^***^2,5,^ ^↓^***^3,4^	15.77 ± 0.14 ^↑^***^2,5,^ ^↓^***^3,4^	11.85 ± 0.11 ^↓^***^1,3,4,6,^**^5^	13.55 ± 0.15 ^↓^***^1,3,4,6^	15.43 ± 0.19 ^↑^***^1,2,5,6^	18.52 ± 0.19 ^↑^***^1,2,5,6^	15.19 ± 0.14 ^↑^***^1,2,5,6^	17.89 ± 0.23 ^↑^***^1,2,5,6^	12.57 ± 0.20 ^↑^**^2,^ ^↓^***^1,3,4,^ **^6^	13.62 ± 0.28 ^↓^***^1,3,4,6^	13.35 ± 0.16 ^↑^***^2,^ **^5,^ ^↓^***^3,4^	15.46 ± 0.13 ^↑^***^2,5,^ ^↓^***^3,4^
SL	7.26 ± 0.06 ^↑^***^2,5,^ ^↓^***^3,4,^**^6^	8.96 ± 0.10 ^↑^***^2,^*^5,^ ^↓^***^3,4^	5.82 ± 0.08 ^↓^***^1,3,4,5,6^	6.85 ± 0.15 ^↓^***^1,3,4,5,6^	8.28 ± 0.09 ^↑^***^1,2,5,6,^ ^↓^*^4^	10.37 ± 0.06 ^↑^***^1,2,5,6^	8.64 ± 0.10 ^↑^*^3,^***^1,2,5,6^	10.58 ± 0.12 ^↑^***^1,2,5,6^	6.42 ± 0.10 ^↑^***^2,^ ^↓^***^1,3,4,6^	8.22 ± 0.32 ^↓^*^1,^***^3,4,^**^6,^ ^↑^***^2^	7.56 ± 0.07 ^↑^**^1,^***^2,5,^ ^↓^***^3,4^	9.19 ± 0.15 ^↑^***^2,^**^5,^ ^↓^***^3,4^
SD	9.9 ± 0.1 ^↓^***^4,5^	12.50 ± 0.20 ^↓^***^4,^**^5^	10.1 ± 0.2 ^↓^***^4,^**^5^	12.70 ± 0.40 ^↓^*^4,5^	10.3 ± 0.1 ^↓^***^4,5^	12.90 ± 0.40 ^↓^*^4^	11.1 ± 0.1 ^↑^***^1,2,3,6^	13.80 ± 0.10 ^↑^***^1,^*^2,3^**^6^	11.36 ± 0.25 ^↑^***^1,3,6,^**^2^	14.42 ± 0.68 ^↑^**^1,^*^2,6^	10.1 ± 0.1 ^↓^***^4,5^	13.00 ± 0.20 ^↓^**^4,^*^5^
CD	9.17 ± 0.10 ^↓^***^3,4,^ ^↑^***^5^	10.10 ± 0.07 ^↑^**^2,5,^ ^↓^***^3,4^	9.08 ± 0.14 ^↓^***^3,4,↑^**^5^	9.29 ± 0.26 ^↓^**^1,^***^3,4,^*^6^	10.47 ± 0.10 ^↑^***^1,2,5,6^	11.52 ± 0.15 ^↑^***^1,2,5,6^	10.76 ± 0.13 ^↑^***^1,2,5,6^	11.83 ± 0.24 ^↑^***^1,2,5,6^	8.32 ± 0.16 ^↓^***^1,3,4,6,^**^2^	9.52 ± 0.15 ^↓^**^1,^***^3,4^	9.18 ± 0.09 ^↓^***^3,4,^ ^↑^***^5^	9.97 ± 0.13 ^↑^*^2,^ ^↓^***^3,4^
PA	65.90 ± 0.60 ^↓^***^3,5,^*^4^	69.30 ± 1.10 ^↓^**^5^	67.50 ± 0.70 ^↓^**^3,^***^5^	68.90 ± 0.80 ^↓^**^5^	70.40 ± 0.90 ^↑^***^1,^**^2,6,^*^4,↓^*^5^	71.20 ± 2.50	67.90 ± 0.80 ^↑^*^1,↓^*^3,^***^5^	69.70 ± 1.40 ^↓^**^5^	75.93 ± 2.21 ^↑^***^1,2,4,6,^*^3^	77.00 ± 2.61 ^↑^**^1,2,^*^4,^***^6^	67.30 ± 0.70 ^↓^**^3,^***^5^	69.10 ± 0.70 ^↓^***^5^
PA/BW	71.09	53.76	81.52	61.19	60.53	40.52	50.90	37.96	69.47	56.53	73.55	58.02
EN	123.5 ± 3.5		133.0 ± 3.0		123.0 ± 3.0		122.0 ± 2.0		162.5 ± 2.5		81.5 ± 1.5	
EW	47.0 ± 1.0		57.5 ± 0.5		55.0 ± 1.0		48.5 ± 0.5		57.5 ± 0.5		39.0 ± 1.0	
IPI	6.60		10.06		5.10		4.23		8.57		3.70	

^1^ M, mean; SEM, standard error of the mean; *n*, number of birds per breed/sex. ^2^ As summarized in [52]. Significant differences (within a trait): * *p* < 0.05, ** *p* < 0.01, and *** *p* < 0.001 (^↑^ increase; ^↓^ decrease) relative to breeds ^1,2,3,4,5^ or ^6^. Breeds: RK, Russian Korolyok; CB, Cochin Bantam; HBSS, Hamburg Bantam Silver Spangled; PWB, Polish White-crested Black; RWD, Red White-tailed Dwarf; SW, Silkie White. Sex: ♀, females; ♂, males. Traits measured: BW, body weight, g; BL, body length, cm; SL, shank length, cm; SD, shank diameter, mm; CD, chest depth, cm; PA, pectoral angle, °; PA/BW, specific PA index; EN, egg number; EW, egg weight, g; IPI, Narushin’s Integral Performance Index [52,53,54,56]. Data for EN, EW and IPI were produced by the authors and taken from Vakhrameev et al. [54].

**Table 2:** Indicators of genetic diversity ^1^ in the studied chicken breeds.

	Breed	*n*	*A_R_*	*H_O_*	*H_E_*	*F*_IS_
1	CB	54	1.774 ± 0.002 ^↑^***^2,4,^*^6,↓^***^3,5^	0.228 ± 0.001 ^↑^***^2,↓^***^3,4,5,6^	0.263 ± 0.001 ^↑^***^2,4,↓^***^3,5,6^	0.116 ± 0.001 ^↑^***^2,3,4,5,6^
2	HBSS	25	1.556 ± 0.002 ^↓^***^1,3,4,5,6^	0.200 ± 0.001 ^↓^***^1,3,4,5,6^	0.186 ± 0.001 ^↓^***^1,3,4,5,6^	−0.040 ± 0.001 ^↓^***^1,3,5,↑^***^4^
3	PWB	36	1.895 ± 0.001 ^↑^***^1,2,4,5,6^	0.308 ± 0.001 ^↑^***^1,2,4,6,↓^***^5^	0.306 ± 0.001 ^↑^***^1,2,4,6,↓^***^5^	0.001 ± 0.001 ^↓^***^1,↑^***^2,4,5,6^
4	RK	40	1.698 ± 0.002 ^↓^***^1,3,5,6,↑^***^2^	0.252 ± 0.001 ^↑^***^1,2,↓^***^3,5,6^	0.232 ± 0.001 ^↑^***^2,↓^***^1,3,5,6^	−0.060 ± 0.001 ^↓^***^1,2,3,5,6^
5	RWD	19	1.889 ± 0.001 ^↑^***^1,2,4,6,↓^***^3^	0.330 ± 0.001 ^↑^***^1,2,3,4,6^	0.319 ± 0.001 ^↑^***^1,2,3,4,6^	−0.033 ± 0.001 ^↓^***^1,3,↑^***^2,4,6^
6	SW	44	1.767 ± 0.002 ^↓^*^1,^***^3,5,↑^***^2,4^	0.290 ± 0.001 ^↑^***^1,2,4,↓^***^3,5^	0.275 ± 0.001 ^↑^***^1,2,4,↓^***^3,5^	−0.041 ± 0.001 ^↓^***^1,3,5,↑^***^4^

^1^ *A_R_*, allelic diversity; *H_O_*, observed heterozygosity; *H_E_*, expected heterozygosity; *F*_IS_, inbreeding coefficient. Significant differences (within a trait): * *p* < 0.05, and *** *p* < 0.001 (^↑^ increase; ^↓^ decrease) relative to breeds ^1,2,3,4,5^ or ^6^. Breeds: CB, Cochin Bantam; HBSS, Hamburg Bantam Silver Spangled; PWB, Polish White-crested Black; RK, Russian Korolyok; RWD, Red White-tailed Dwarf; SW, Silkie White.

**Table 3:** Runs of homozygosity (ROHs) and candidate genes annotated within them.

GGA ^1^	ROHs	Annotated Genes within ROHs
1	3,688,051–4,987,231	*PODXL*, *MKLN1*, *K123*, *IL2RA*, *ITIH2*, *ATP5C1*, *TAF3*, *GATA3*
	52,677,319–55,590,991	*LARGE1*, *TIMP3*, *FBXO7*, *PWP1*, *CRY1*, ***TMEM263***, ^2^ *POLR3B*, *TCP11 × 2*, *C12orf75*, *SLC41A2*, *TXNRD1*, *GLT8D2*, *TDG*, *C12orf73*, *HSP9OB1*, *PAH*, *ASCL1*, *IGF1*, *PMCH*, *NUP37*, *CHPT1*, *DRAM1*, *CCDC53*
	75,548,455–78,805,452	*SLC2A14*, *NANOG*, *AICDA*, *PHC1*, *OVST*, *CD86*, *CSTB*, *CCDC58*, *FAM162A*, *KPNA1*, *TAPBPL*, *SCNN1A*, *VAMP1*, *NCAPD2*, *MRPL51*, *CNP1*, *GAPDH*, *IFFO1*, *ING4*, *ZNF384*, *PIANP*, *COPS7A*, *MLF2*, *PTMS*, *CD4*, *GPR162*, *GNB3*, *USP5*, *TPI1*, *ENO2*, *CDCA3*, *ATN1*, *C12orf57*, *EMG1*, *PTPN6*, *PHB2*, *LPCAT3*, *C1S*, *C1R*, *PEX5*, *EPHA1*, *ZYX*, *FAM131B*, *CASP2*, *RAP1GAP3*, *GSTK1*, *EPHB6*, *PRSS2*
	156,375,568–163,630,641	*UCHL3*, *MZT1*, *DACH1*, *TDRD*, *KLH1*, *PCDH20*
	189,025,664–191,763,199	*NOX4*, *TYR*, *GRM5*, *CTSC*, *FZD4*, *EED*, *RAB38*, *TMEM135*, *PRSS23*, *FZD4*, *ME3*, *RAB30*, *DLG2*, *PRCP*, *TMEM126A*, *CCDC89*, *PICALM*
2	**80,850,520–83,898,189**	***GRB10***, ***RPRD1A***, ***GALNT1***, ***VSTM2A***, ***FHOD3***
	141,620,636–148,345,259	*LRLC6*, *RHF20L1*, *TG*, *SLA*, *WISP1*, *ST3GAL1*, *COL22A1*, *AGO2*, *RPLP1*, *PTK2*, *PTP4A3*, *ARC*, *LY6E*, *ZFAT*, *KHDRBS3*, *FAM135B*, *KCNK9*, *TRAPPC9*, *CHRAC1*, *SLC45A4*, *DENND3*, *TSNARE1*, *GPIHBP1*, *LYPD2*
3	68,715,224–71,511,986	*PREP*, *POPDC3*, *LIN28B*, *HACE1*, *ASCC3*, *CCNC*, *USP45*, *PNISR*, *GRIK2*, *SIM1*
	72,435,078–73,021,083	*MMS22L*, *FHL5*, *UFL1*, *FUT9*, *NDUFAF4*
	73,910,974–75,134,412	*EPHA7*, *MAP3K7*
	93,570,116–93,761,038	*TSSC1*, *ADI1*, *RNASEH1*, *COLEC11*
4	**8,274,461–8,965,874**	***DACH2***, ***CHM***, ***POF1B***, ***APOOL***, ***ZNF711***, ***HDX***
7	23,231,559–26,225,419	*IGFBP5*, *IGFBP2*, *RPL37A*, *MARCH4*, *PECR*, *MAP3K2*, *ERCC3*, *BIN1*, *GYPC*, *CNTNAP5*, *MRAS*, *PTPN4*, *EPB41L5*, *RALB*, *INHBB*, *GLI2*, *TFCP2L1*, *CLASP1*
	**26,318,341–27,858,543**	***EAF2***, ***SLC15A2***, ***SEMA5B***, ***PDIA5***, ***SEC22A***, ***ADCY5***, ***HACD2***, ***MYLK***, ***KALRN***
	29,610,915–29,675,284	*ACTR3*, *SLC35F5*
8	26,048,184–26,572,079	*PLPP3*, *PRKAA2*, *C8B*, *DAB1*
9	6,380,344–8,089,323	*SPSB4*, *SLC25A36*, *PXYLP1*, *EPHA4*, *PAX3*
	14,196,831–15,030,112	*TP63*, *LPP*, *BCL6*, *SST1*, *MASP1*, *RTP2*
	15,955,700–17,602,925	*DVL2*, *AP2M1*, *PSMD2*, *HSPB7L*, *EPHB3*, *POLR2H*, *THPO*, *CHRD*, *FETUB*, *EIF4A2*, *RFC4*, *MCF2L2*, *B3GNT5*, *LAMP3*, *MCCC1*, *DCUN1D1*, *ATP11B*, *FXR1*, *DNAJC19*, *TTC14*, *PEX5L*, *USP13*, *NDUFB5*, *ACTL6A*
10	*16,202,403–17,300,743*	***NR2F2***, ***ARRDC4***, ***FAM169B***, ***IGF1R***, ***SYNM***
	17,809,921–18,875,683	*ALDH1A3*, *LRRK1*, *CHSY1*, *SELENOS*, *PCSK6*, *SNRPA1*, *ANKDD1A*, *CLPX*, *SPG21*, *UBAP1L*, *GNRHR*, *PARP16*, *IGDCC3*, *DPP8*, *HACD3*, *SLC24A1*, *VWA9*, *RAB11A*
13	9,974,440–10,313,250	*CPEB4*, *HMP19*

^1^ GGA, chicken (*Gallus gallus*) chromosome. Regions highlighted in bold are found in over 90% of individuals, and the regions highlighted in italics are common to both RK and HBSS. ^2^ *TMEM263* coincides with the classical *ADW* gene [3]. The corresponding prioritized candidate genes are highlighted in bold.

## Data Availability

The proprietary SNP genotyping data produced and analyzed in this study were generated using the 60K chicken SNP chip produced by Illumina Inc. for the GWMAS Consortium represented by Cobb-Vantress Inc. and Hendrix Genetics B.V. As such, the datasets generated using this chip are confidential and protected as intellectual property or as trade secrets. As a consequence, the SNP genotyping information used in this study was not made public but is kept in a secure database at the RRIFAGB. However, the data can be provided upon reasonable request and can be shared with third parties upon approval from the GWMAS Consortium. The authors affirm that all other data necessary to confirm conclusions reported in this article are present within the article, figures, and tables.

## Supplementary Materials

The following supporting information can be downloaded at: https://www.mdpi.com/article/10.3390/ani16040642/s1, Figure S1: Variations in plumage coloration patterns and other phenotypic characteristics of hens and roosters of the Russian Korolyok breed; Figure S2: Cross-validation error plot showing that the optimal number of ancestral populations was K = 6; Figure S3: LD decay analysis results for six chicken dwarf breeds across 28 chromosomes; Table S1: Density of detected SNPs within runs of homozygosity (ROHs) per 1 Mb; Supplementary Information Box S1: Russian Dwarf (Korolyok) Chicken Breed.

## Abbreviations

The following abbreviations are used in this manuscript:

*ADW*	Autosomal dwarfism gene
BCRECB	Bioresource Collection of Rare and Endangered Chicken Breeds
BL	Body length
BW	Body weight
CB	Cochin Bantam
CD	Chest depth
*DW*	Sex-linked (GGAZ-linked) dwarfism gene
EN	Egg number
EW	Egg weight
GGA	Chicken (*Gallus gallus*) chromosome
GWAS	Genome-wide association study
HBSS	Hamburg Bantam Silver Spangled
HC	Hierarchical clustering
IPI	Integral Performance Index
LD	Linkage disequilibrium
NJ	Neighbor-Joining
PA	Pectoral angle
PA/BW	Specific PA index
PCA	Principal component analysis
PCGs	Prioritized candidate genes
PWB	Polish White-crested Black
RK	Russian Korolyok
ROHs	Runs of homozygosity
RRIFAGB	Russian Research Institute of Farm Animal Genetics and Breeding
RWD	Red White-tailed Dwarf
SD	Shank diameter
SL	Shank length
SNP	Single-nucleotide polymorphism
SW	Silkie White
