# Supplementary material for: Kinglet in the Poultry Court of Russia: Whole-Genome Insights into Ancestry, Genetic Variability, Selection Footprints and Candidate Genes in a Unique Local Chicken Breed Relative to Other Bantam/Dwarf Breeds
Source: Animals (Basel). 2026 Feb 17;16(4):642. doi: 10.3390/ani16040642 (PMC12937304; doi:10.3390/ani16040642)
Supplement: Supplementary file 1 [file animals-16-00642-s001.zip › Supplementary Figure S3.pdf]

Supplementary Figure S3. LD decay analysis results in six chicken dwarf breeds across 28 chromosomes (GGA).

Breeds: RK, Russian Korolyok; CB, Cochin Bantam; HBSS, Hamburg Bantam Silver Spangled; PWB, Polish White-crested Black; RWD, Red White-tailed Dwarf; SW, Silkie White.

RK: GGA1

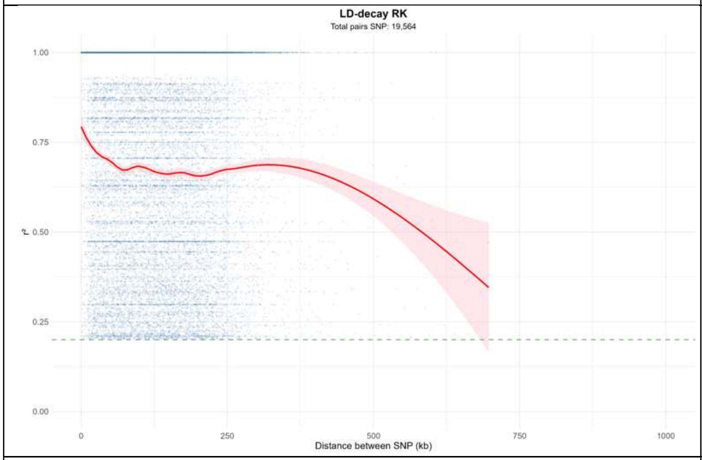

RK: GGA2

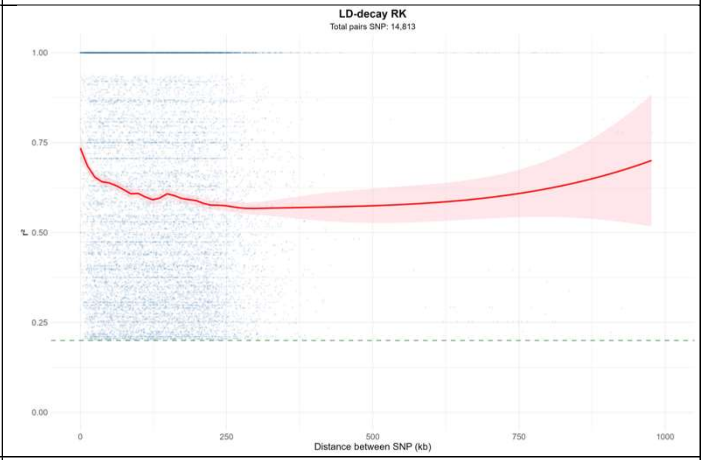

RK: GGA3

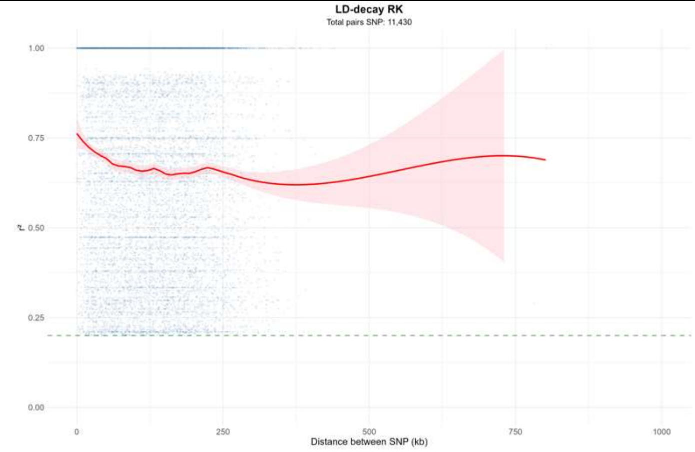

RK: GGA4

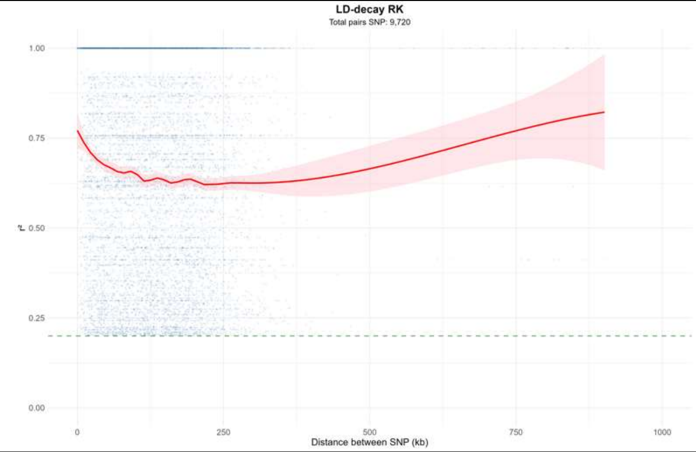

RK: GGA5

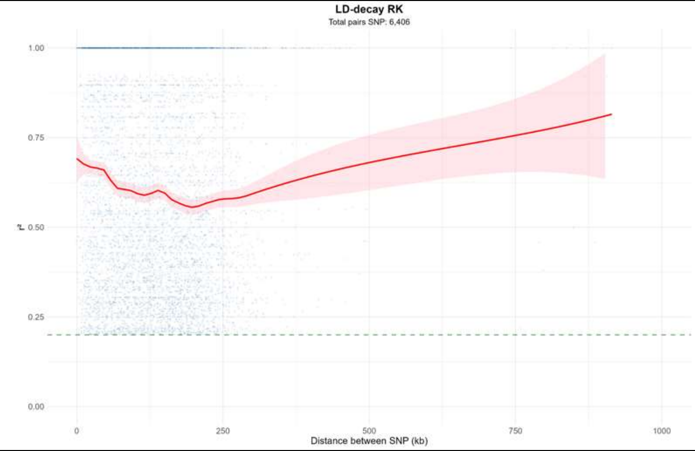

RK: GGA6

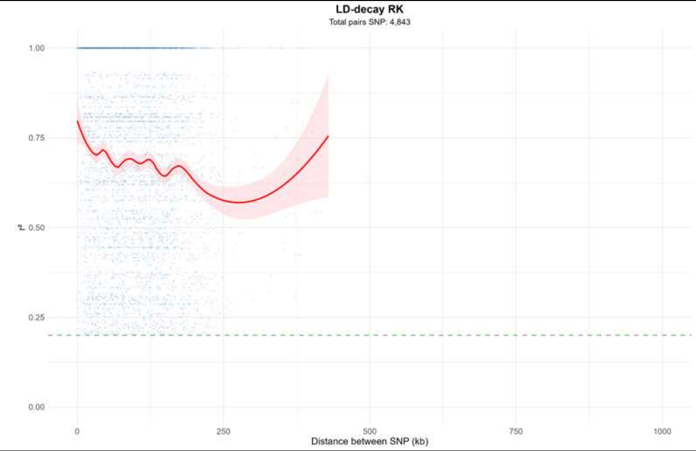

RK: GGA7

RK: GGA8

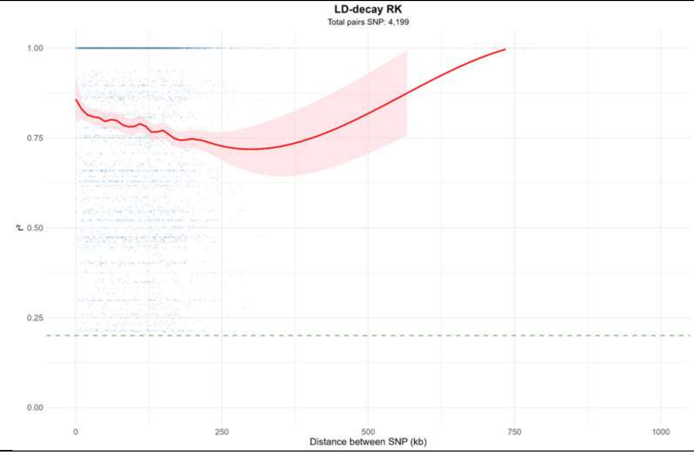

RK: GGA9

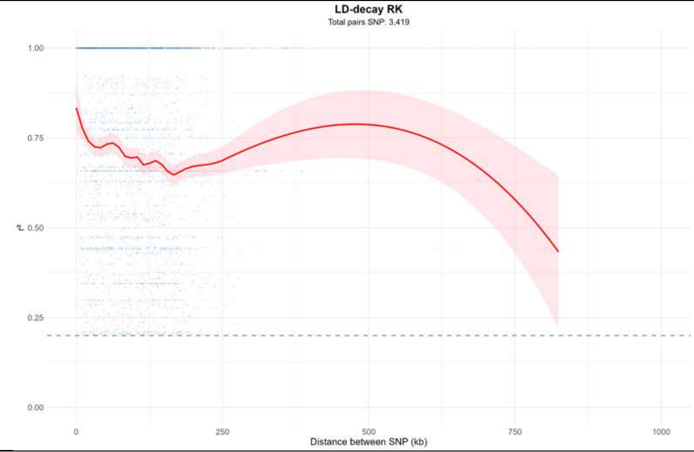

RK: GGA10

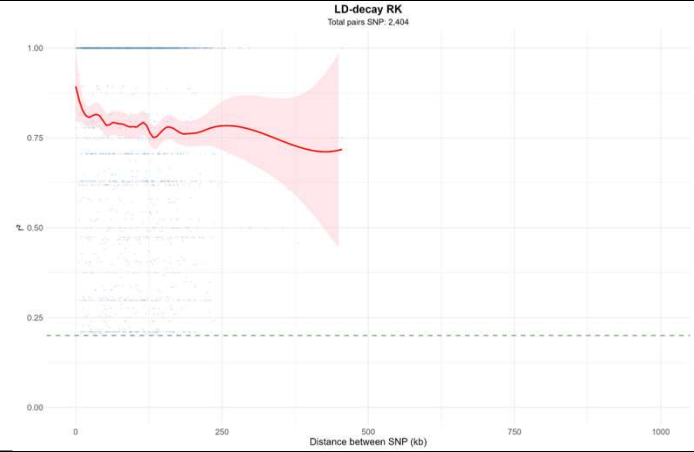

RK: GGA11

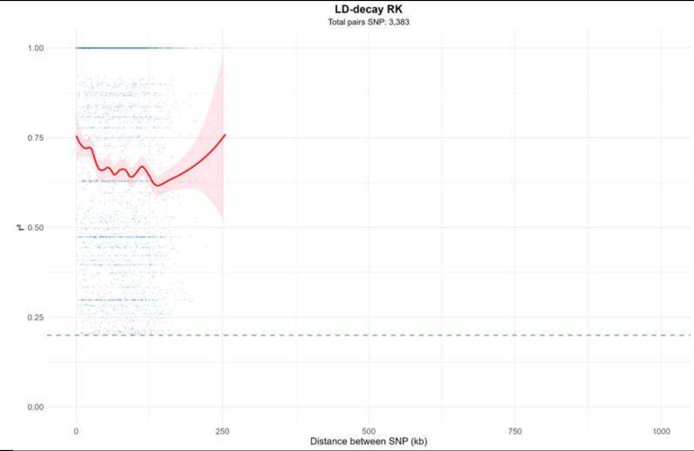

RK: GGA12

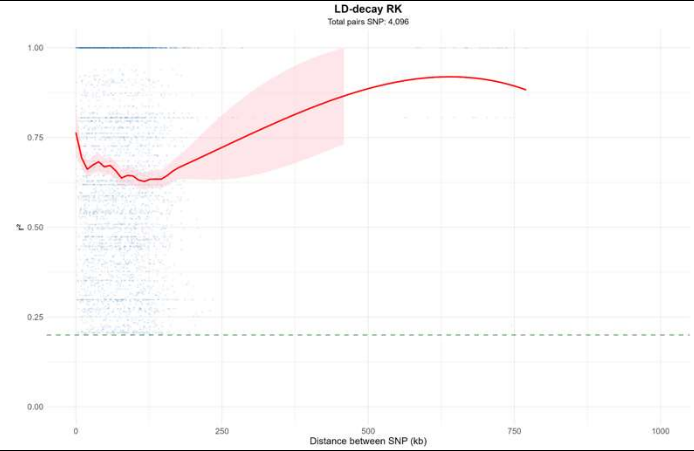

RK: GGA13

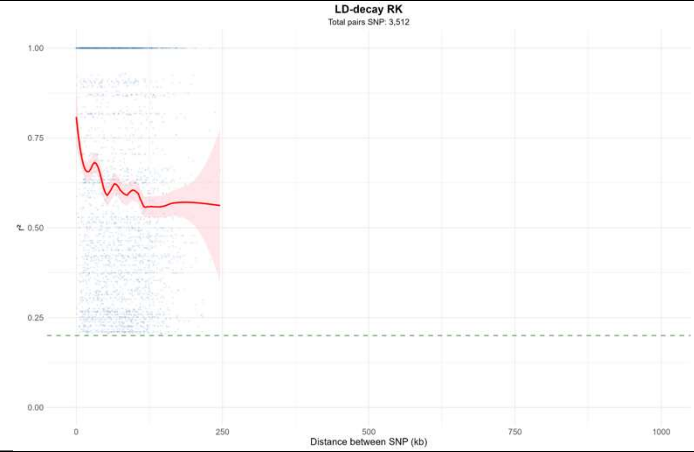

RK: GGA14

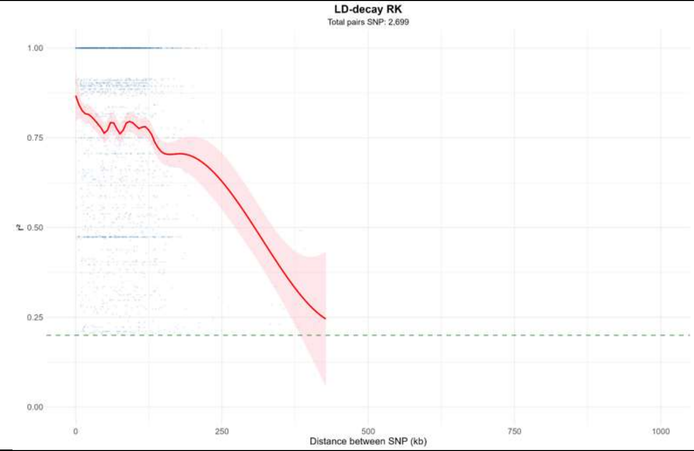

RK: GGA15

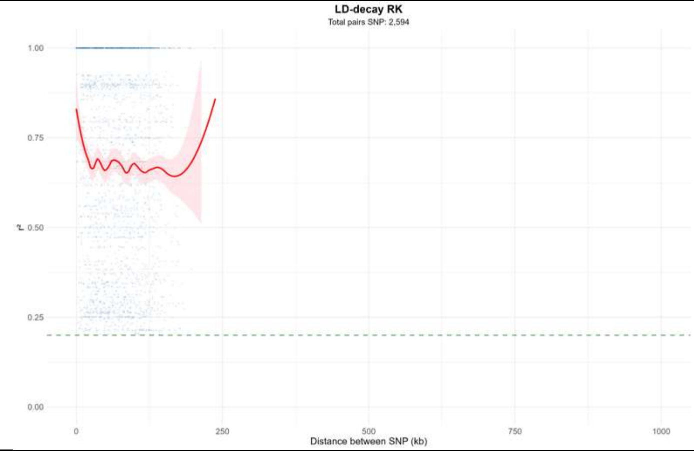

RK: GGA16

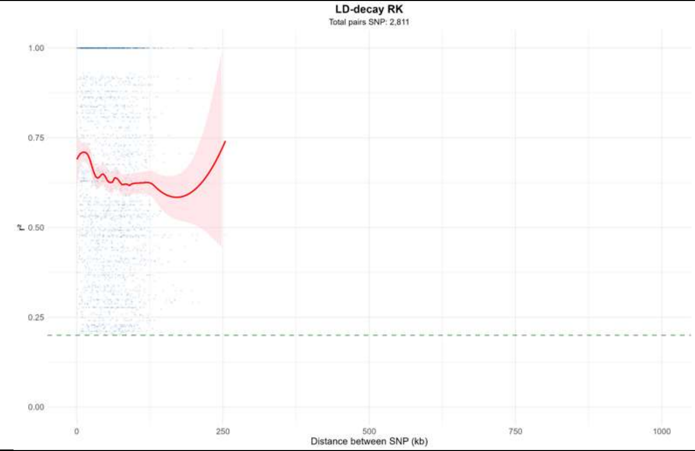

RK: GGA17

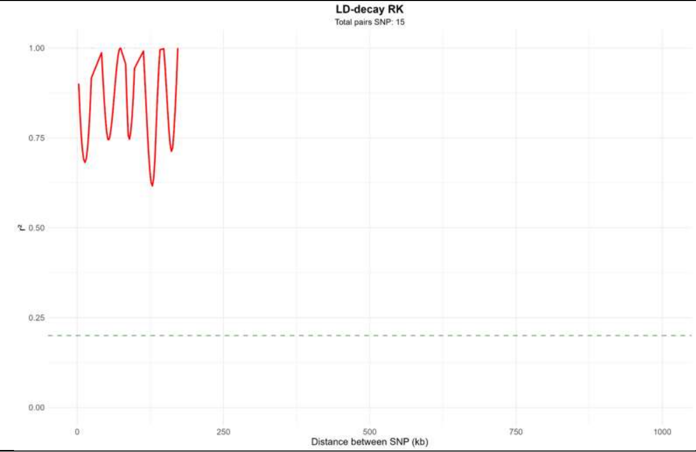

RK: GGA18

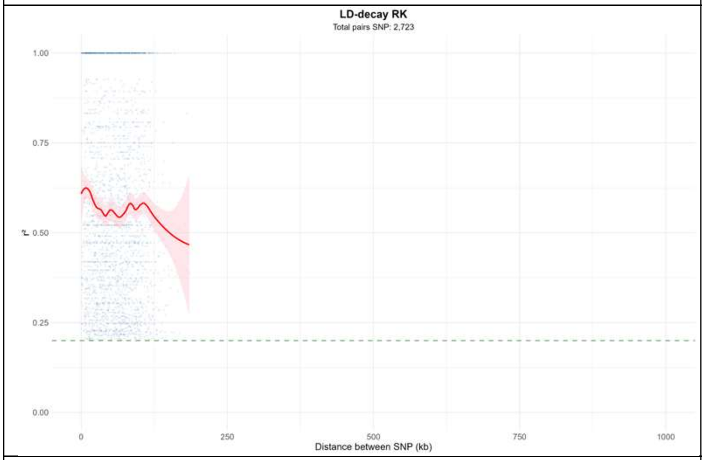

RK: GGA19

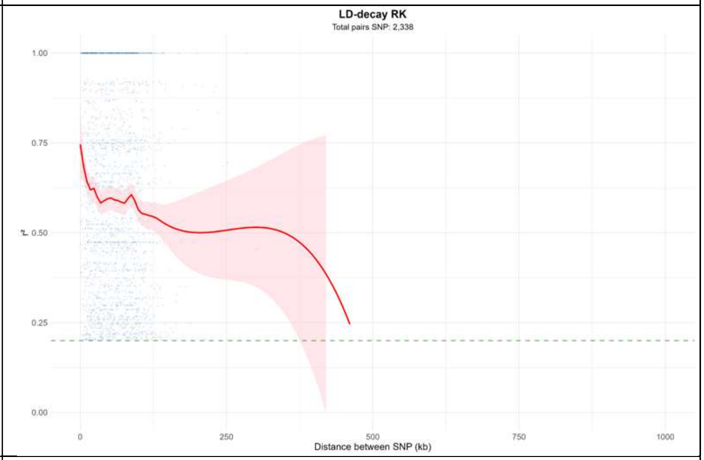

RK: GGA20

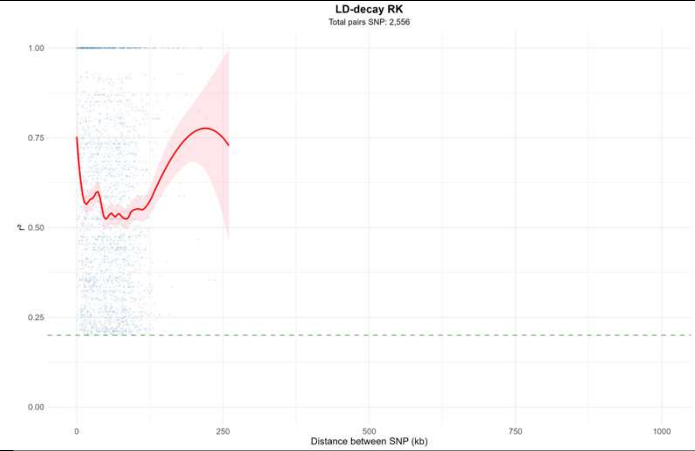

RK: GGA21

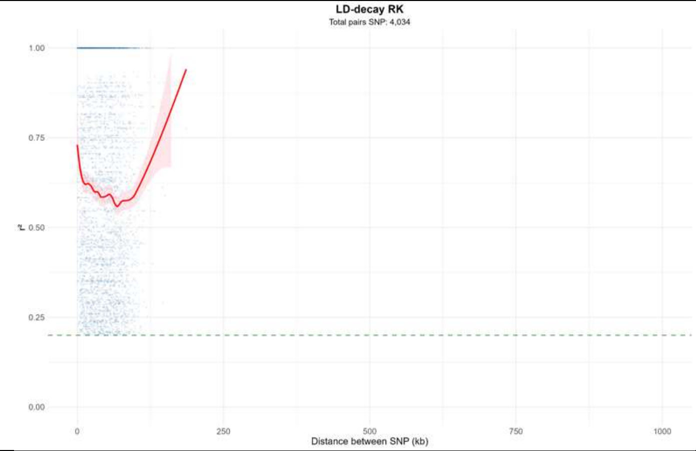

RK: GGA22

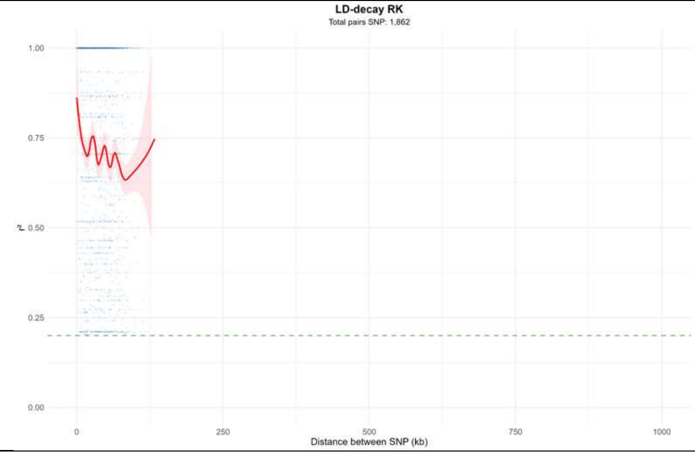

RK: GGA23

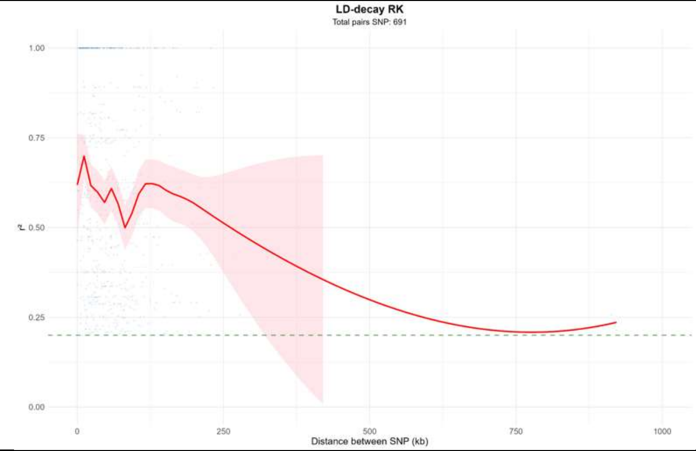

RK: GGA24

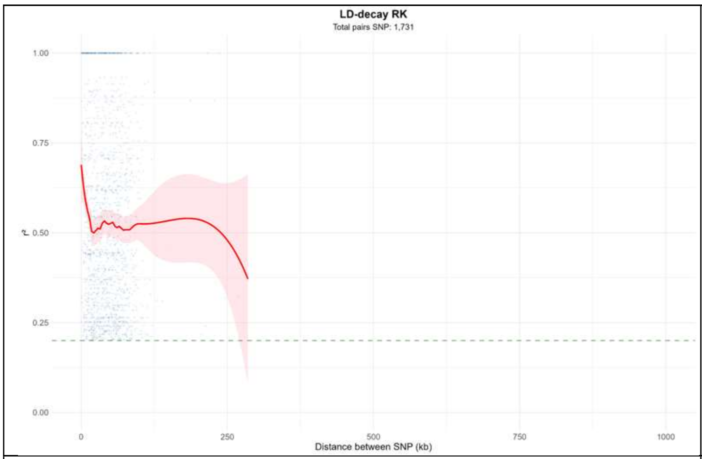

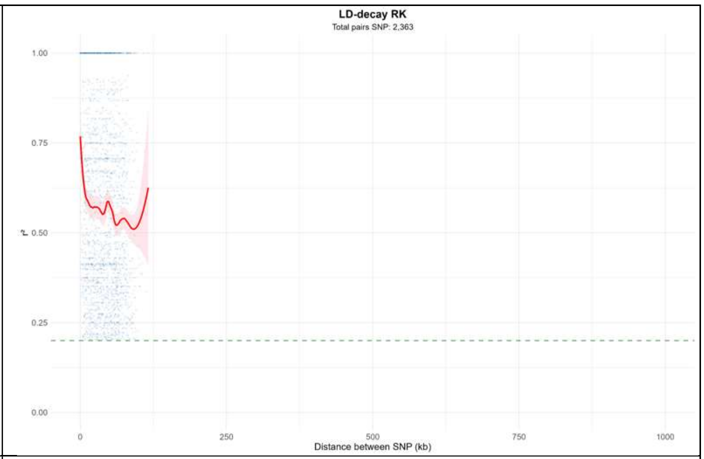

RK: GGA25	RK: GGA26

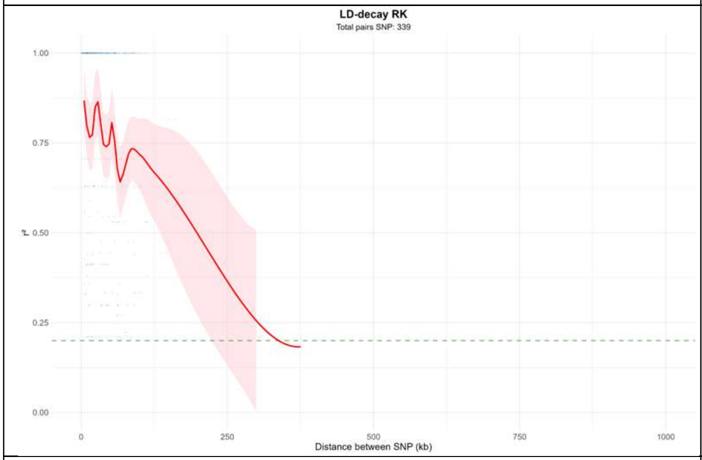

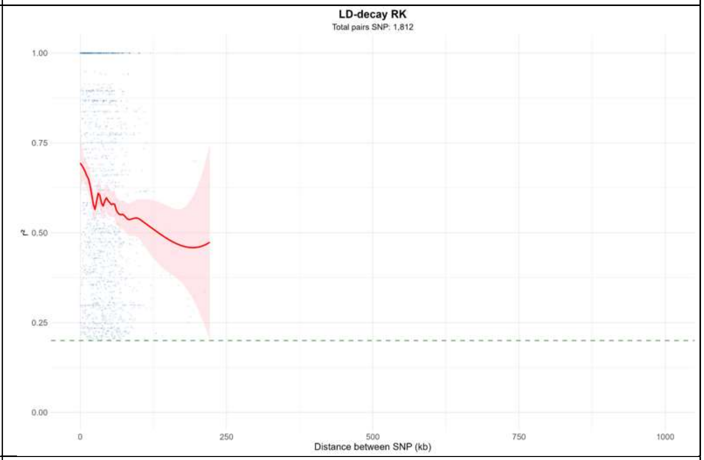

RK: GGA27	RK: GGA28

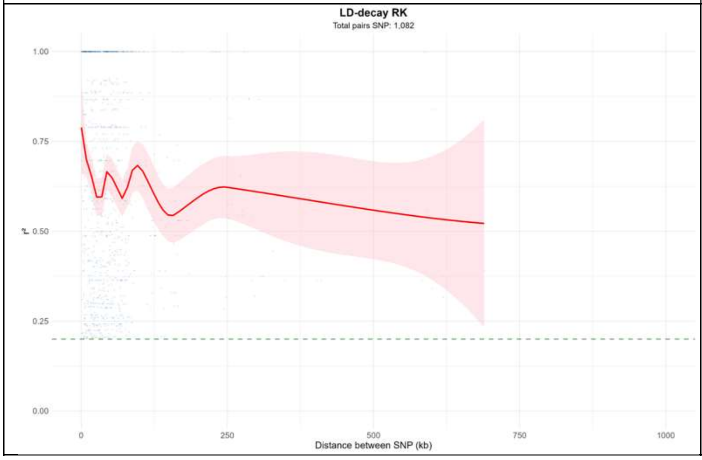

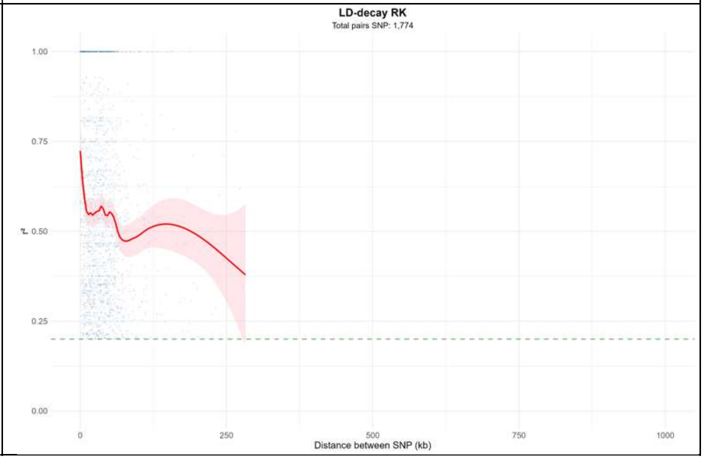

CB: GGA1	CB: GGA2

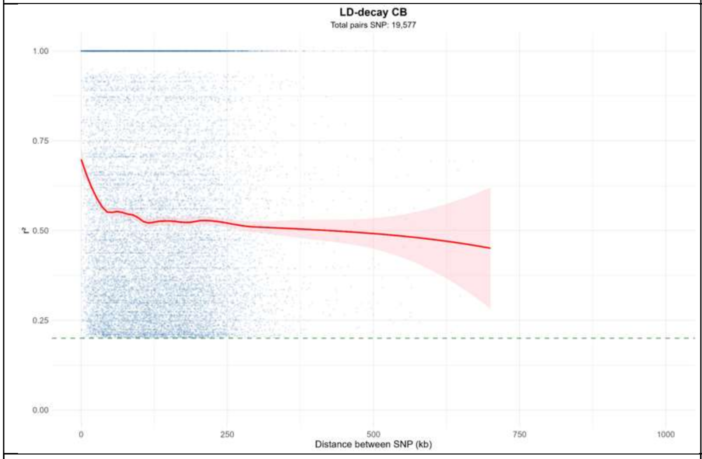

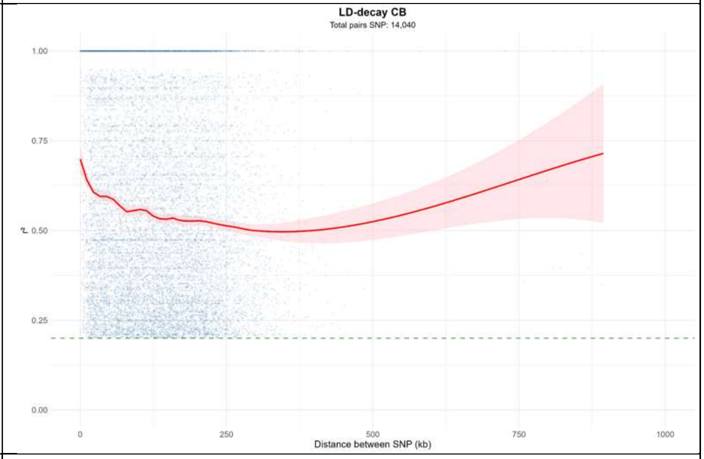

CB: GGA3	CB: GGA4

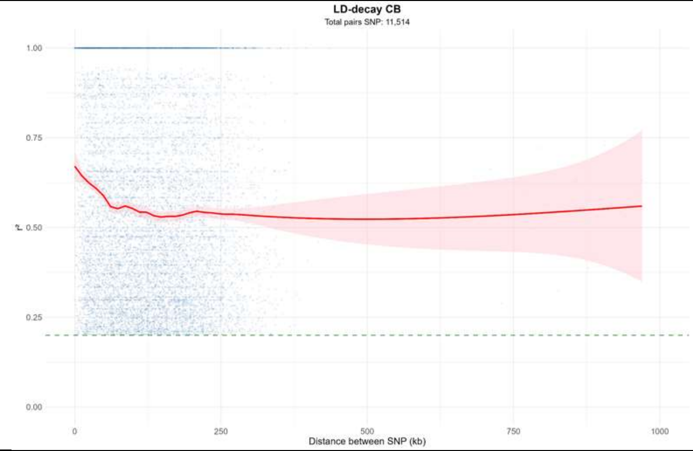

CB: GGA5

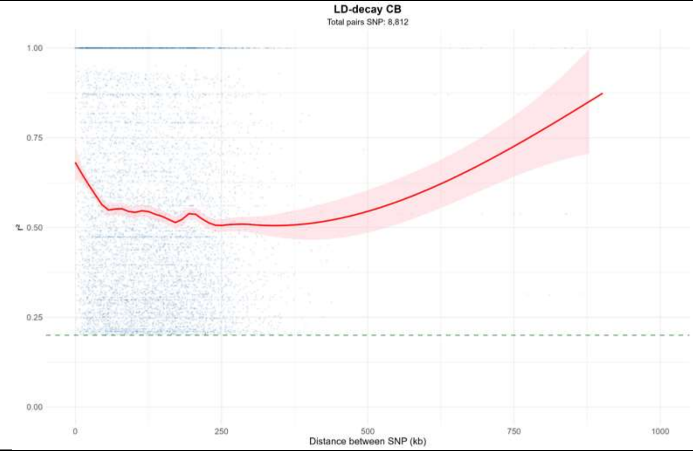

CB: GGA6

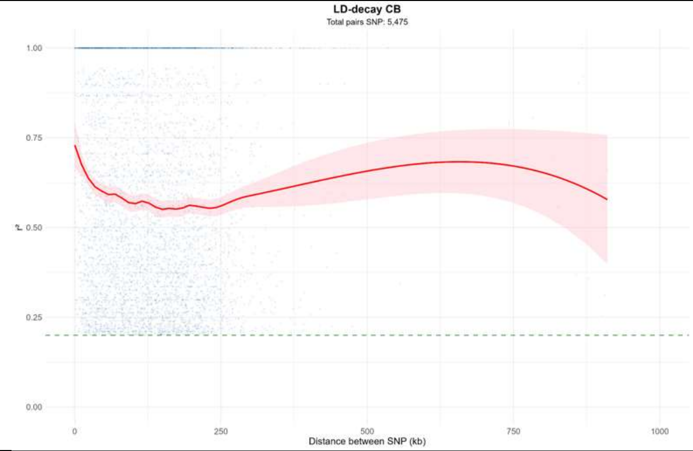

CB: GGA7

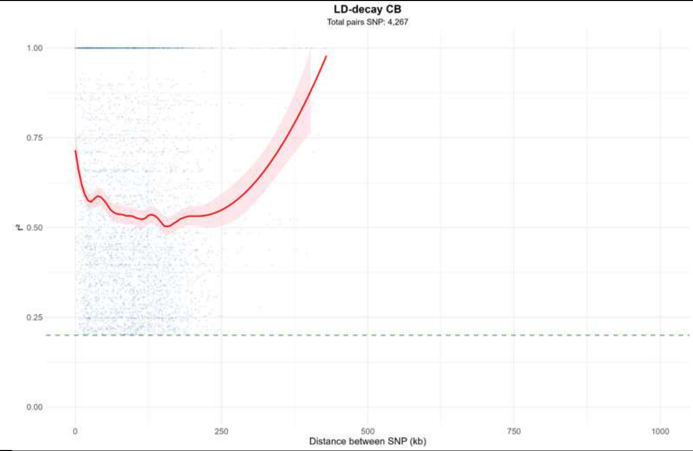

CB: GGA8

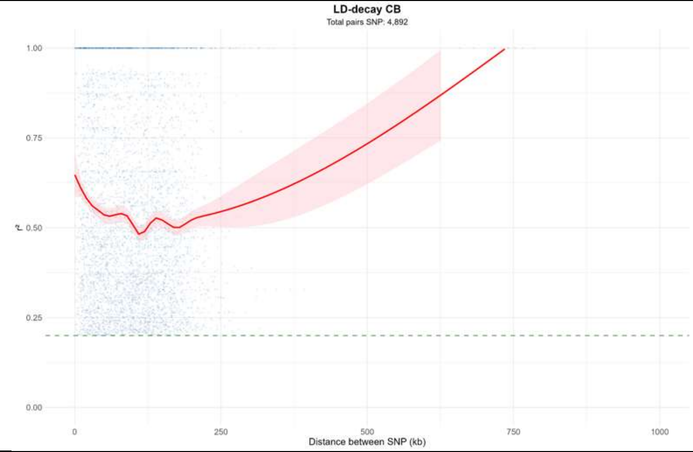

CB: GGA9

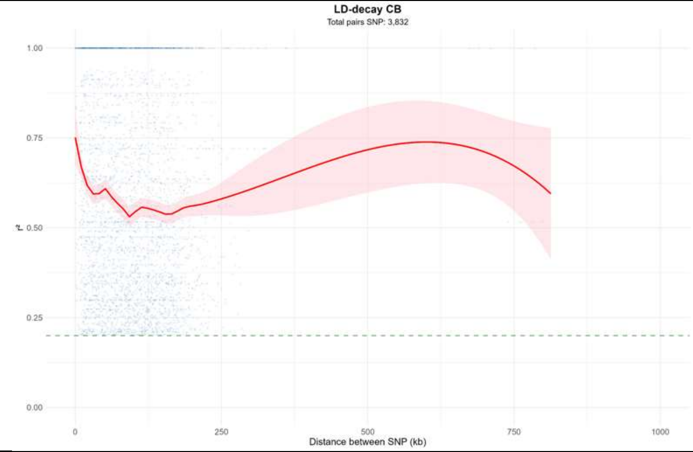

CB: GGA10

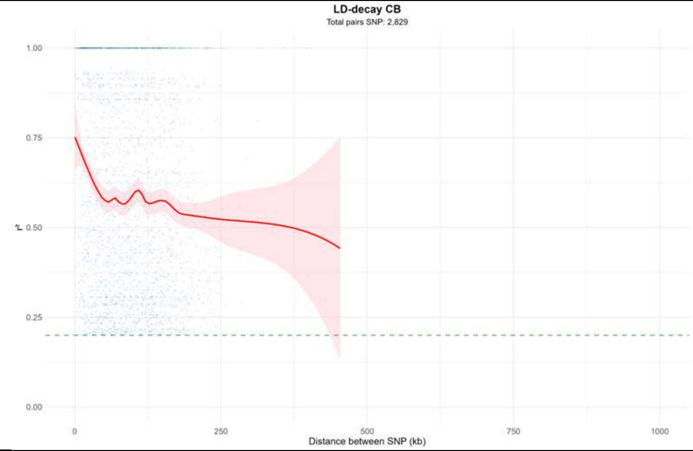

CB: GGA11

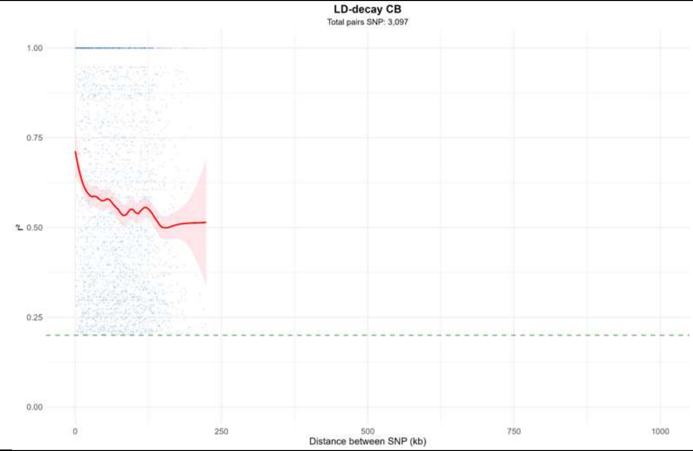

CB: GGA12

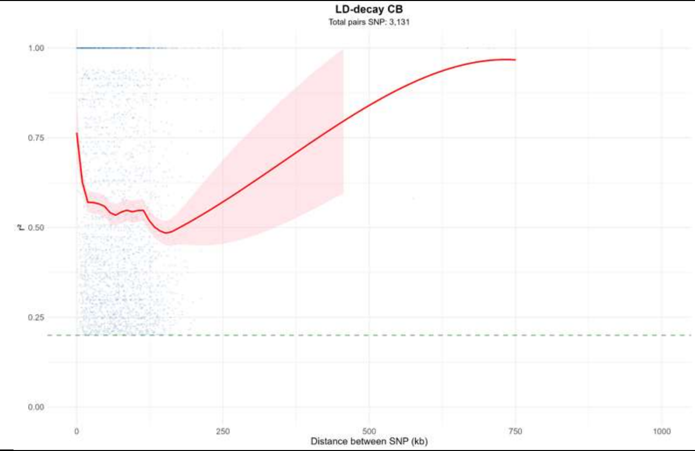

CB: GGA13

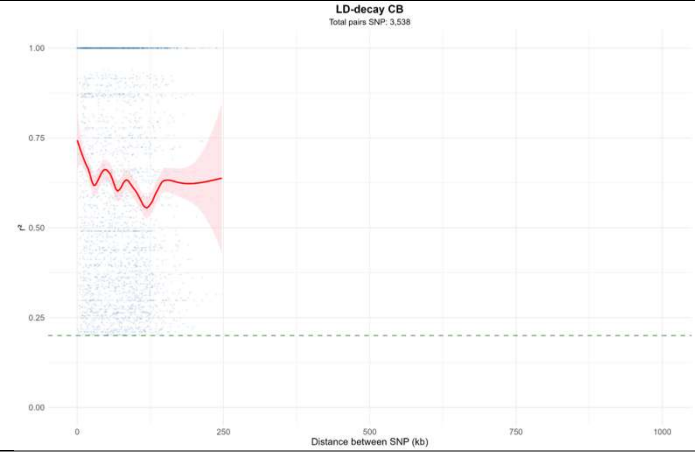

CB: GGA14

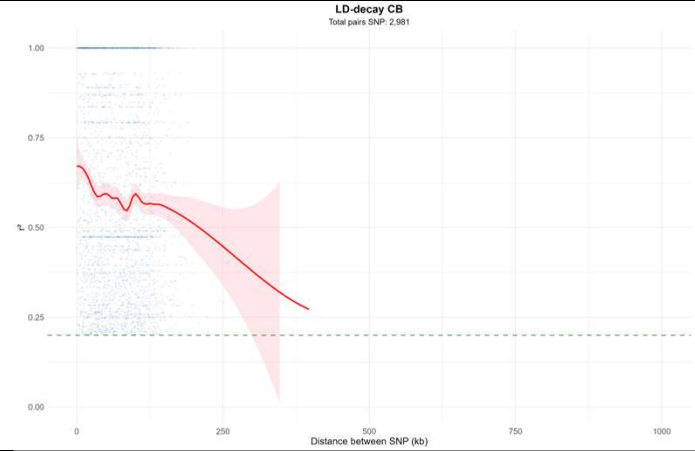

CB: GGA15

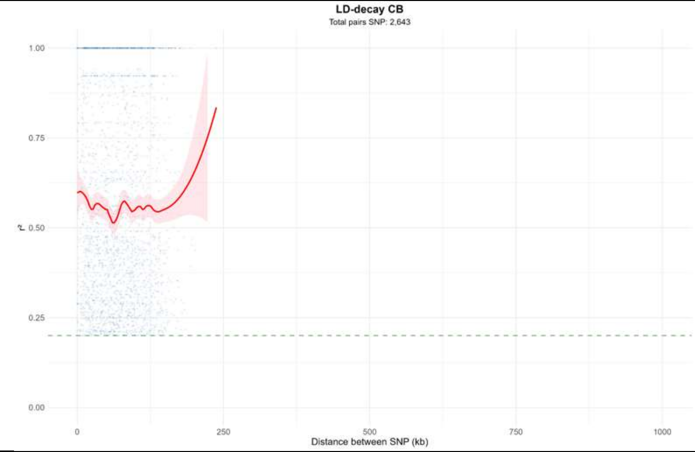

CB: GGA16

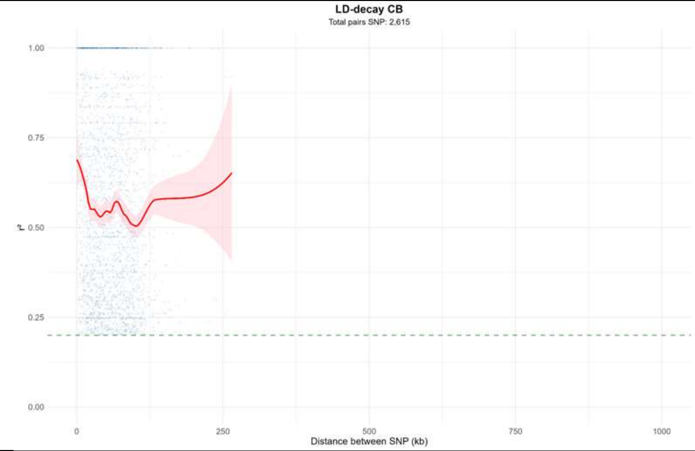

CB: GGA17

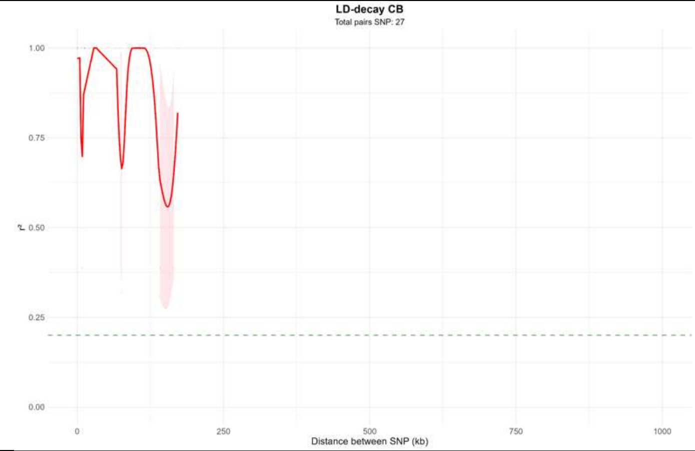

CB: GGA18

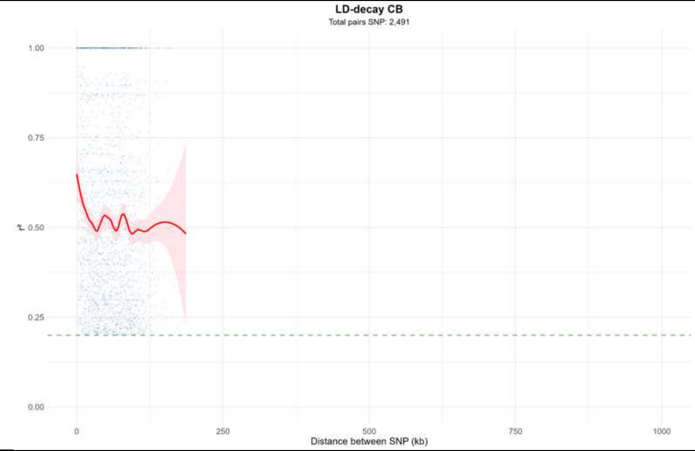

CB: GGA19

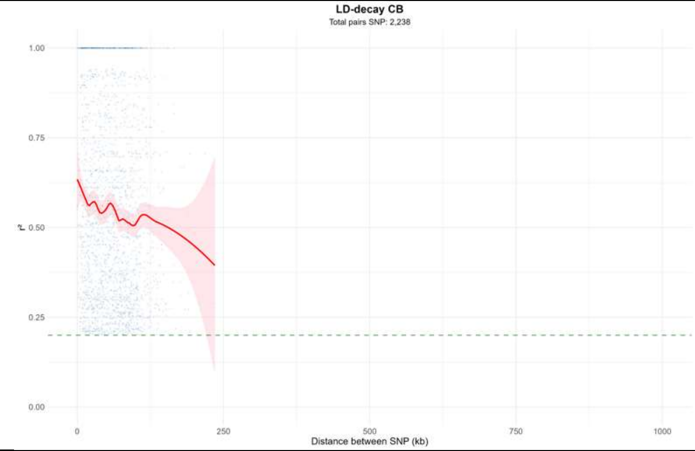

CB: GGA20

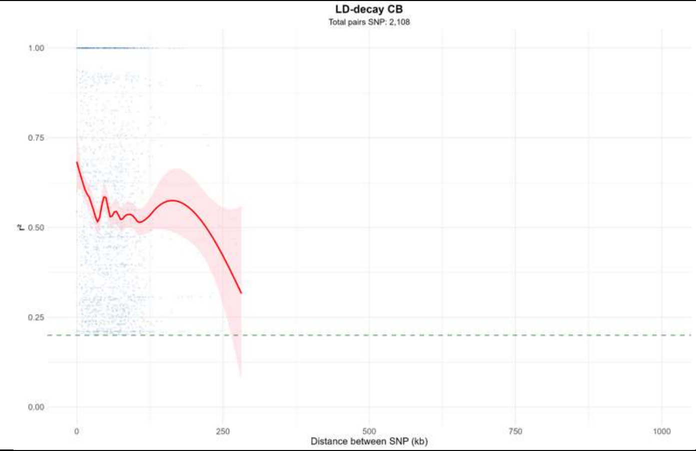

CB: GGA21

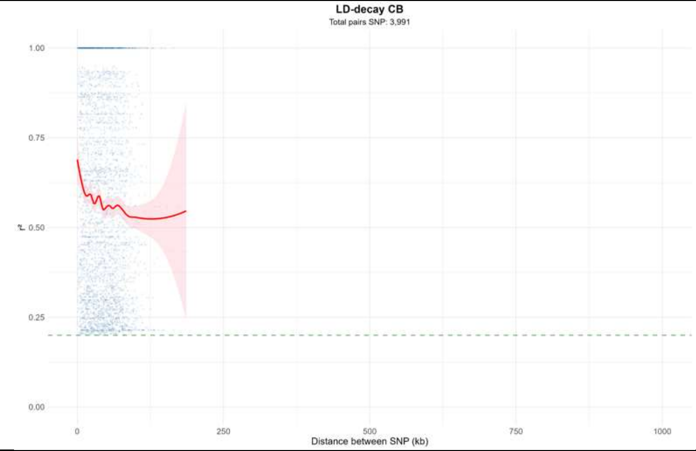

CB: GGA22

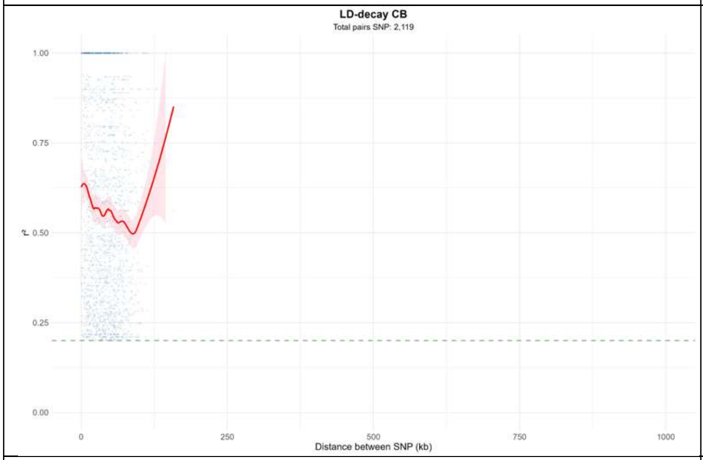

CB: GGA23

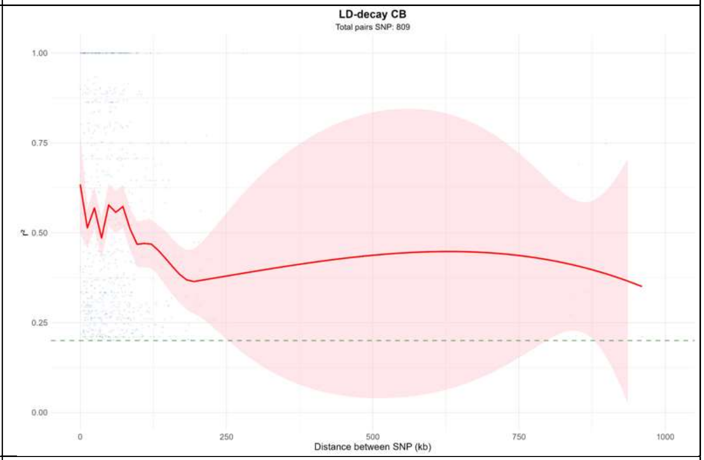

CB: GGA24

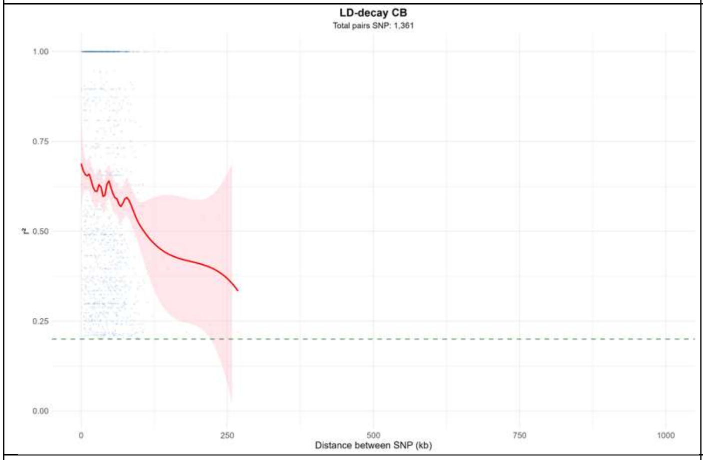

CB: GGA25

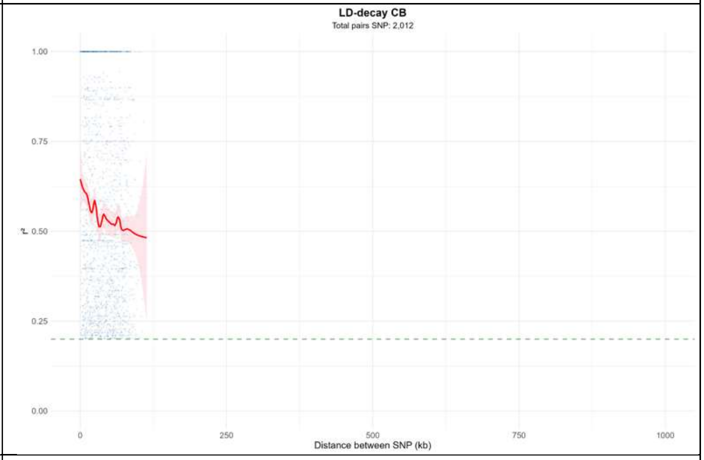

CB: GGA26

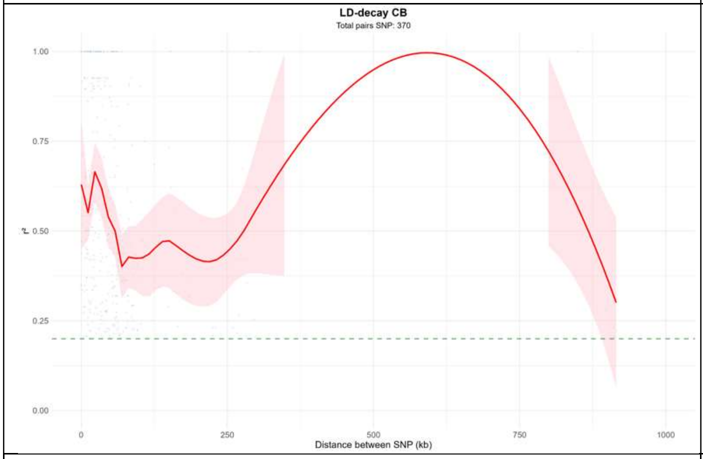

CB: GGA27

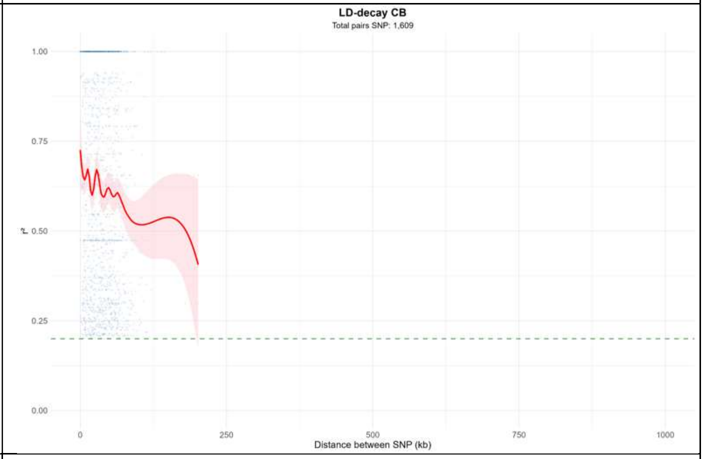

CB: GGA28

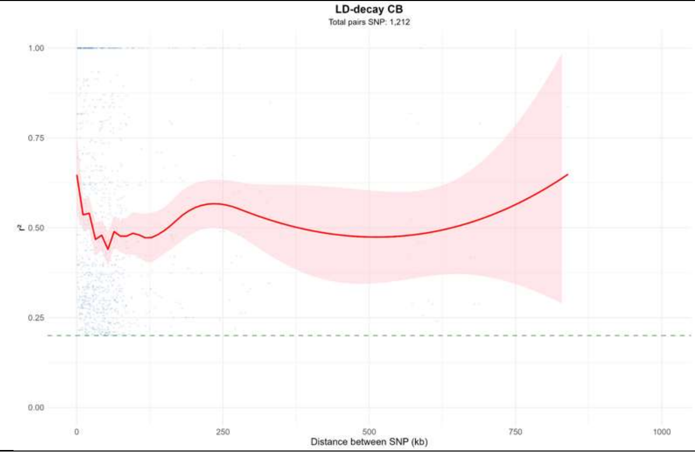

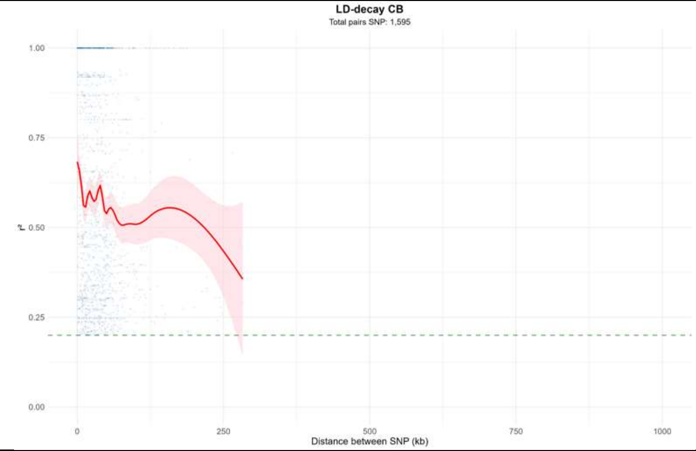

HBSS: GGA1	HBSS: GGA2

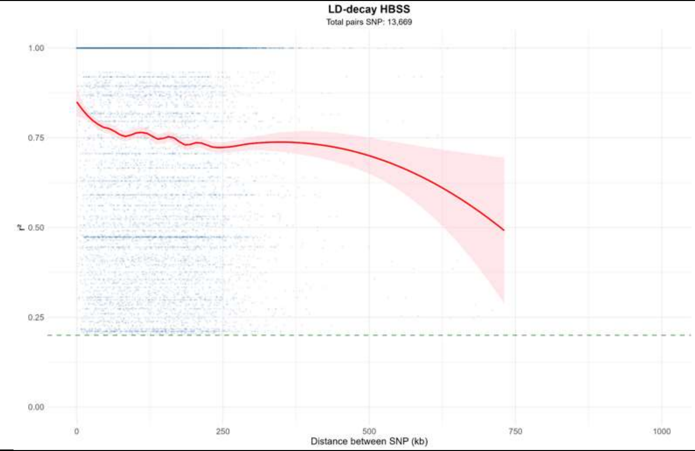

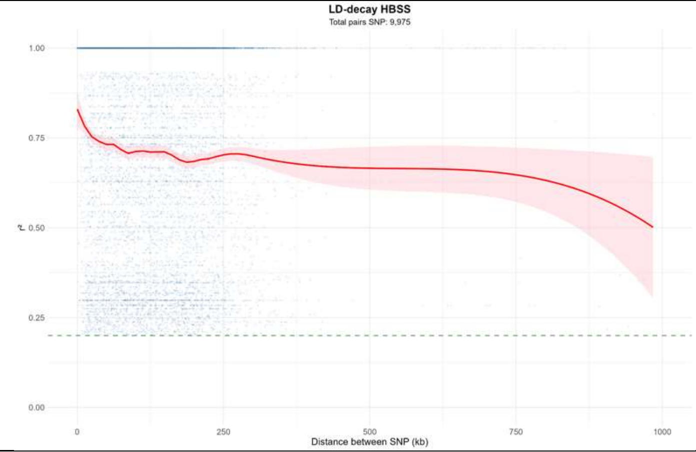

HBSS: GGA3	HBSS: GGA4

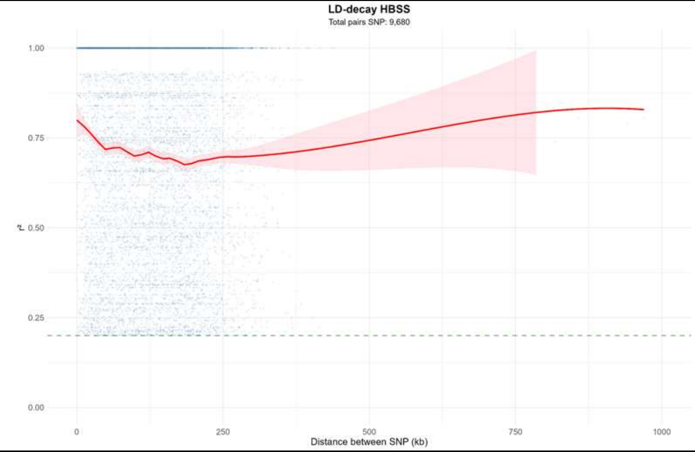

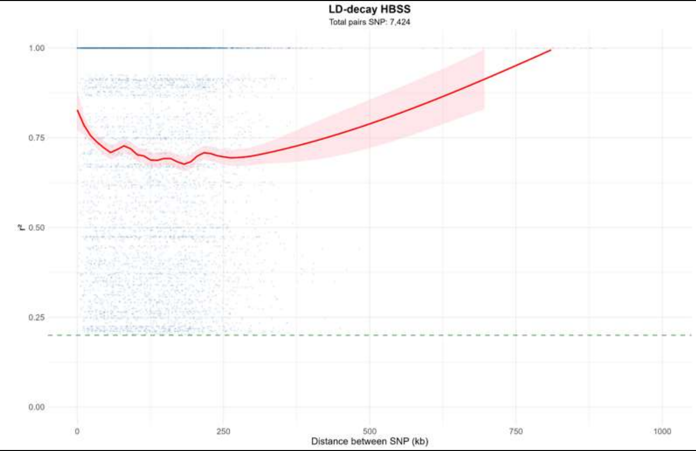

HBSS: GGA5	HBSS: GGA6

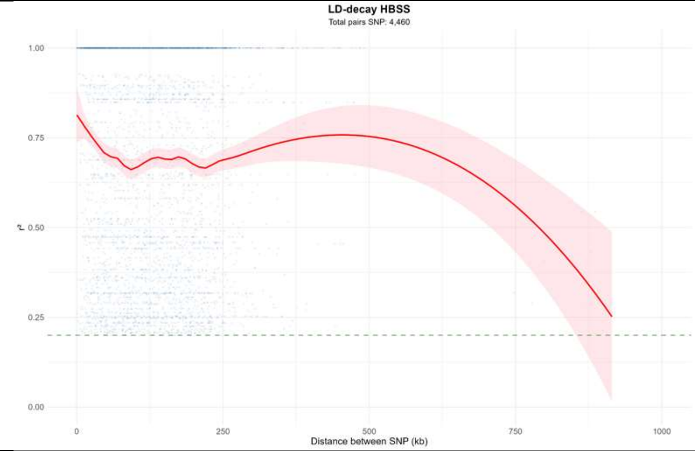

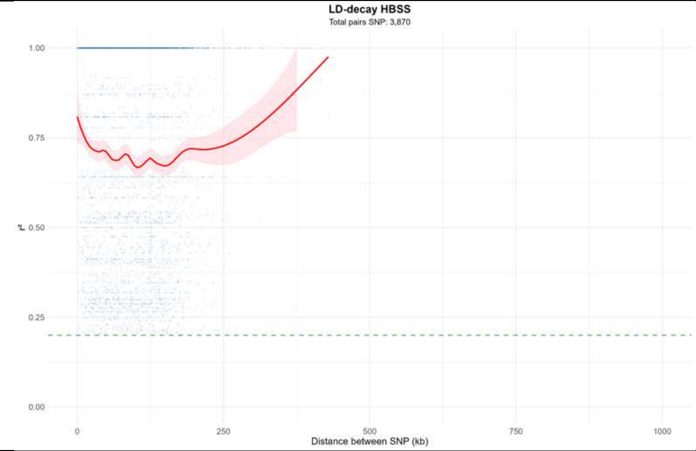

HBSS: GGA7	HBSS: GGA8

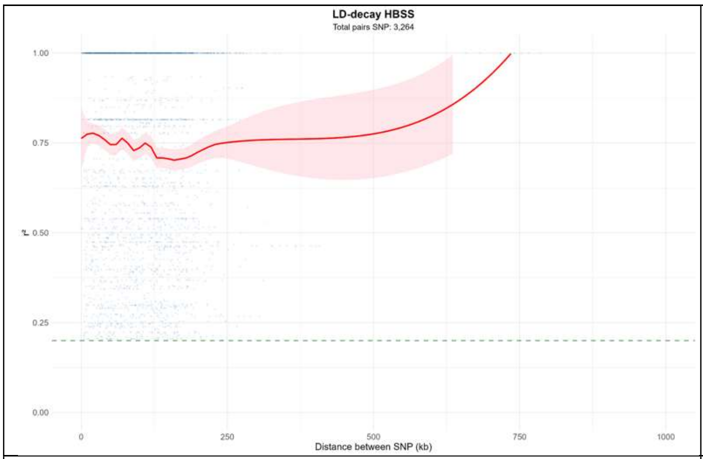

HBSS: GGA9

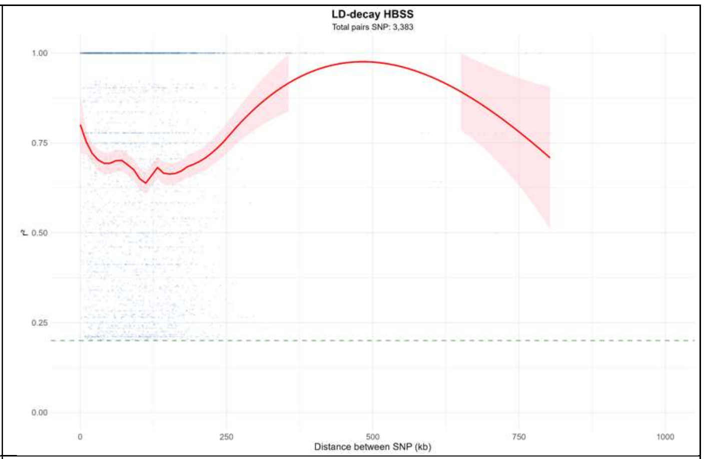

HBSS: GGA10

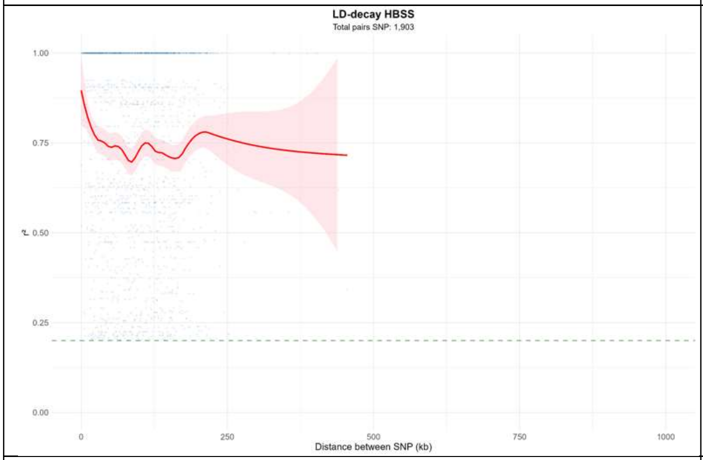

HBSS: GGA11

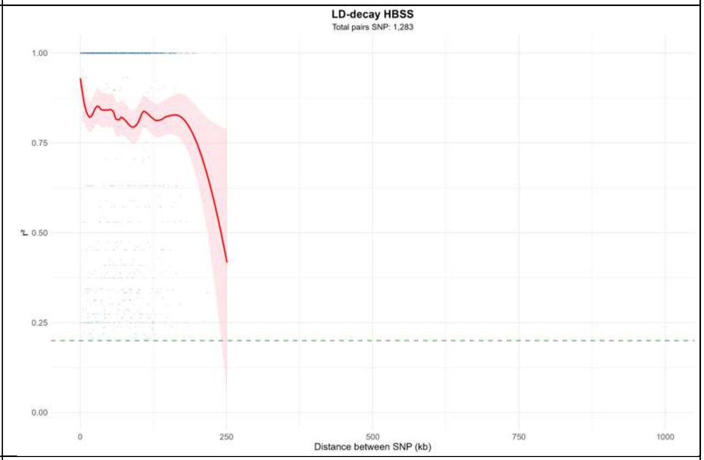

HBSS: GGA12

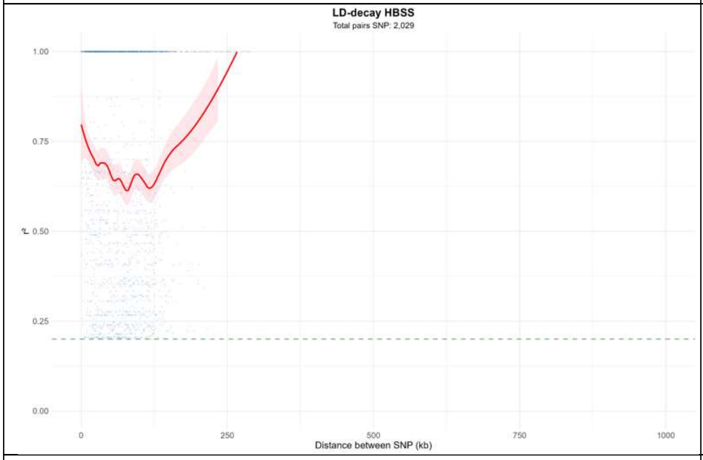

HBSS: GGA13

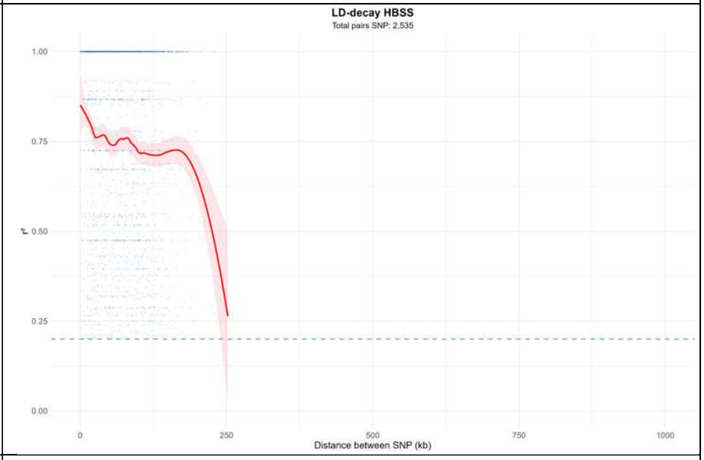

HBSS: GGA14

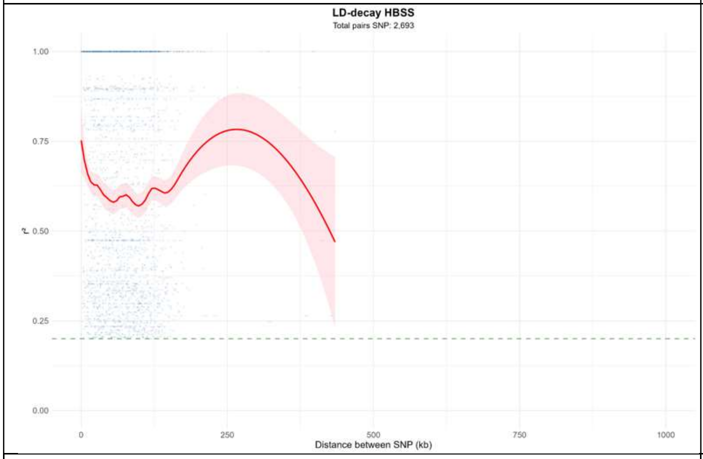

HBSS: GGA15

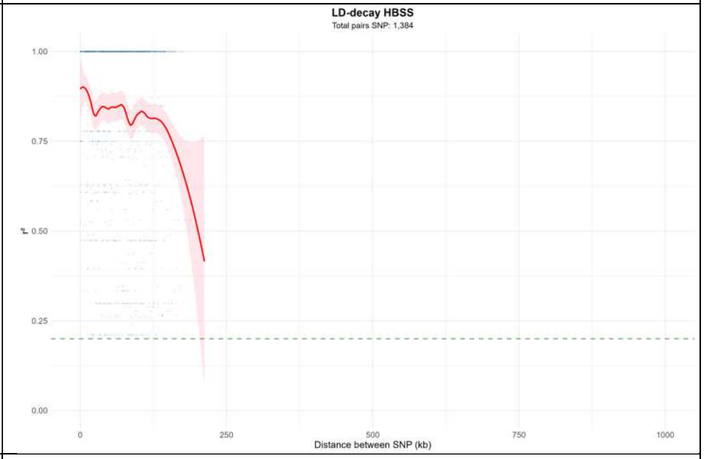

HBSS: GGA16

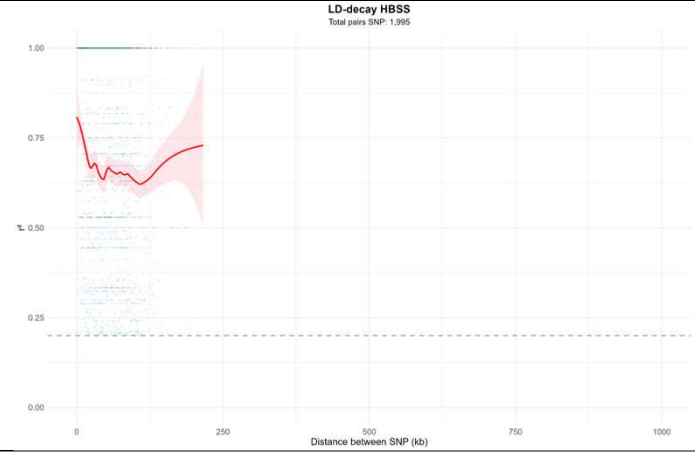

HBSS: GGA17

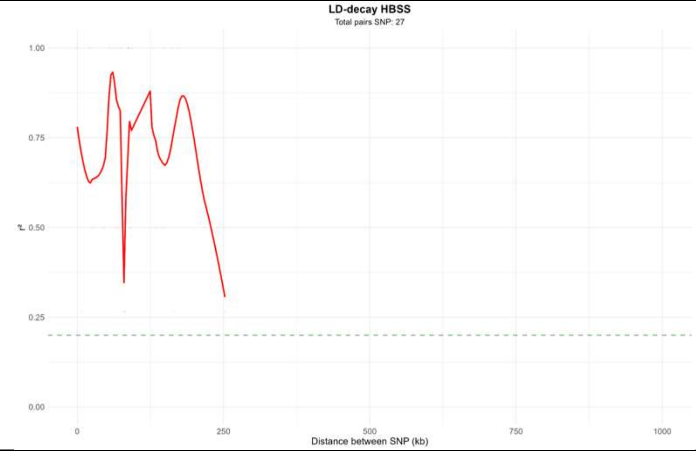

HBSS: GGA18

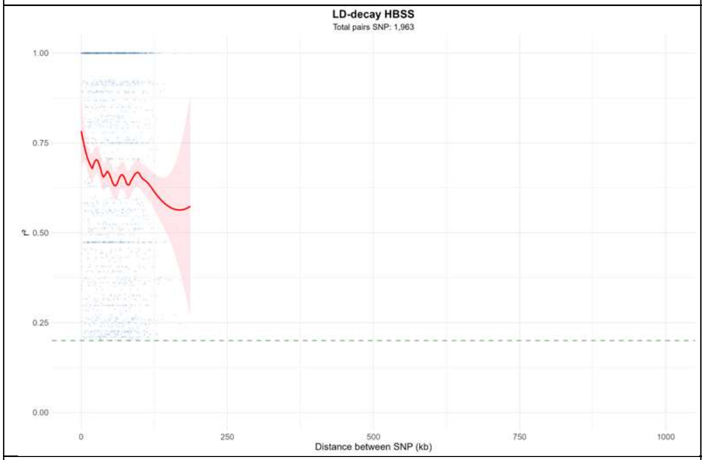

HBSS: GGA19

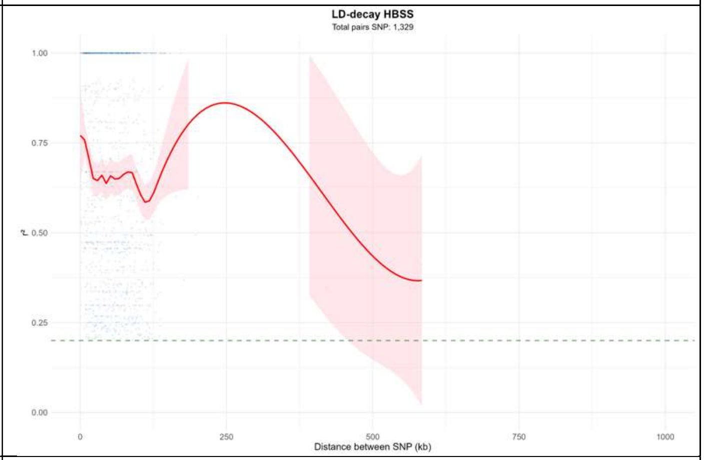

HBSS: GGA20

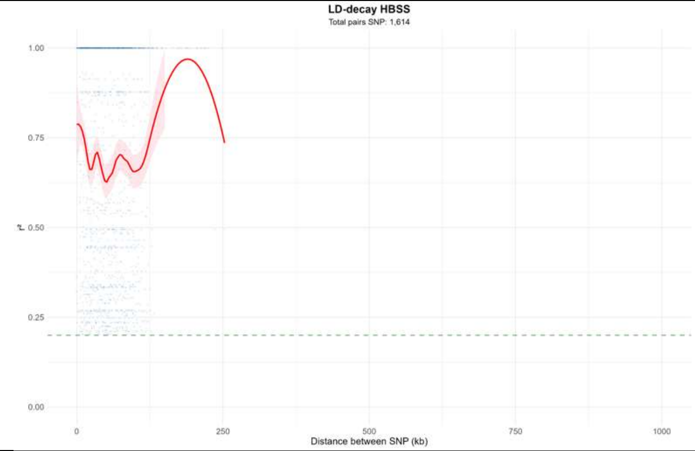

HBSS: GGA21

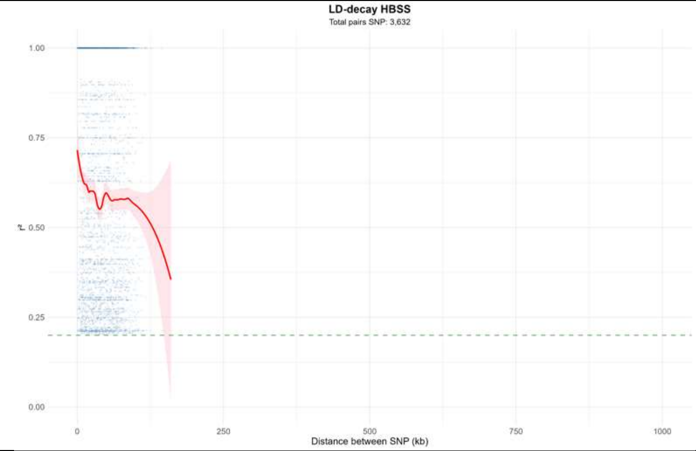

HBSS: GGA22

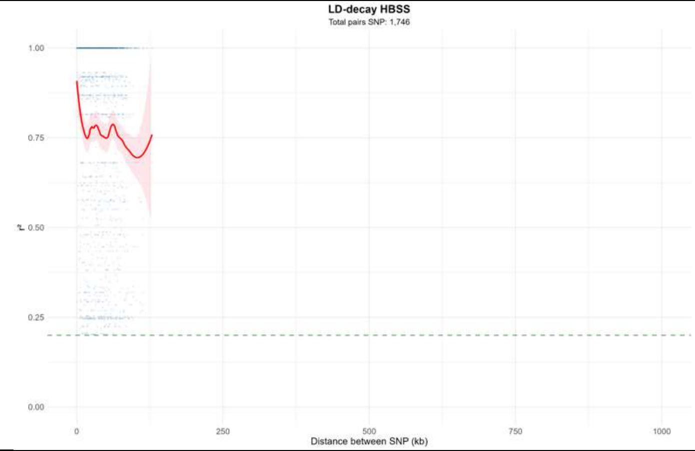

HBSS: GGA23

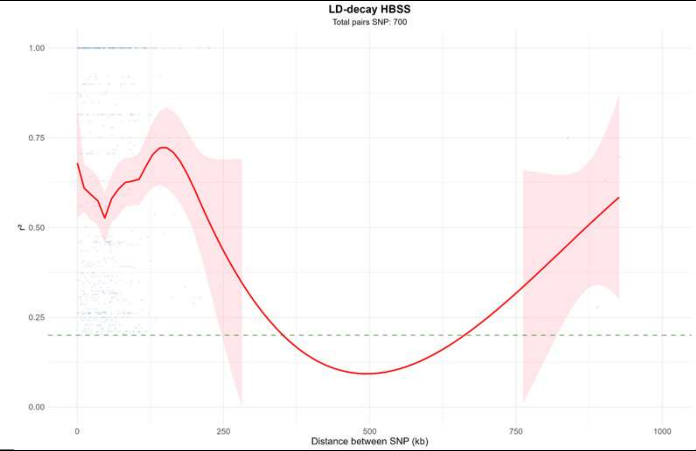

HBSS: GGA24

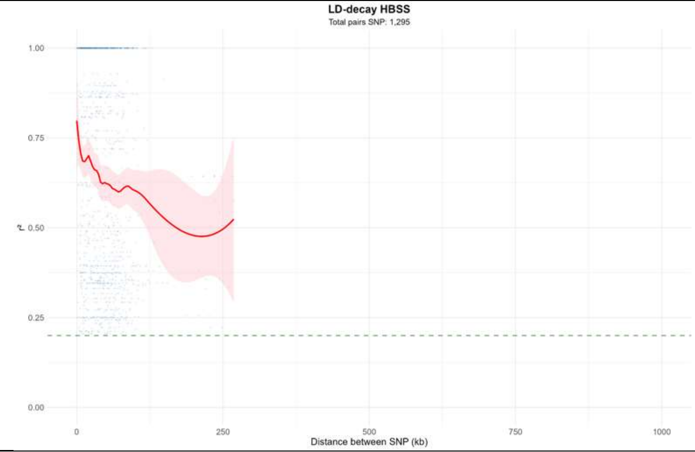

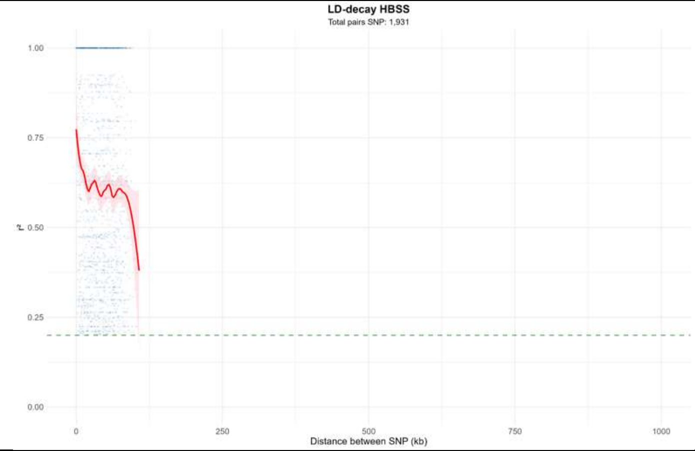

HBSS: GGA25

HBSS: GGA26

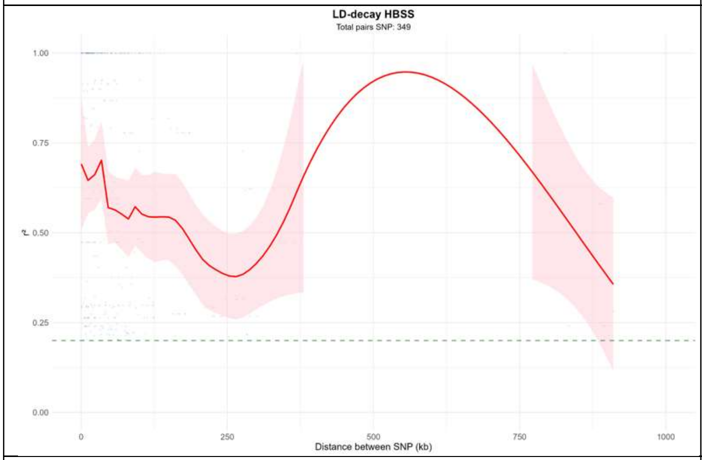

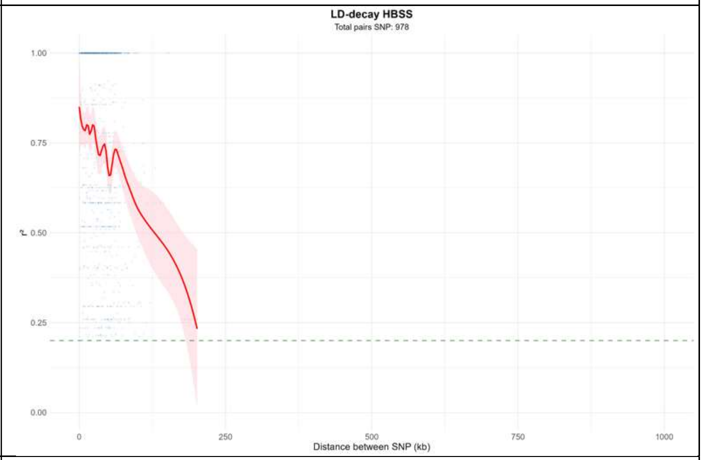

HBSS: GGA27

HBSS: GGA28

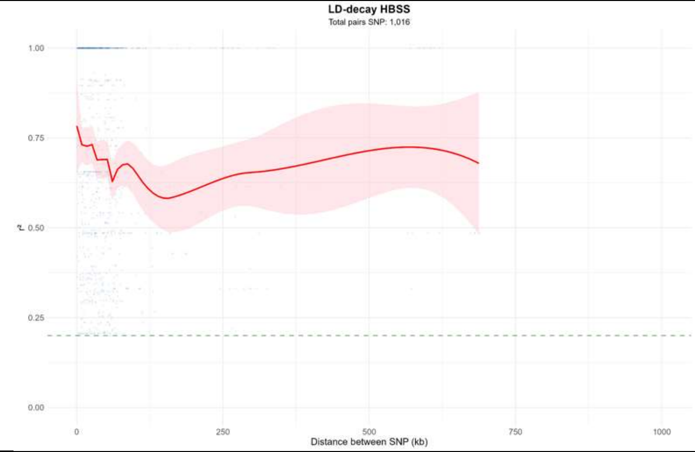

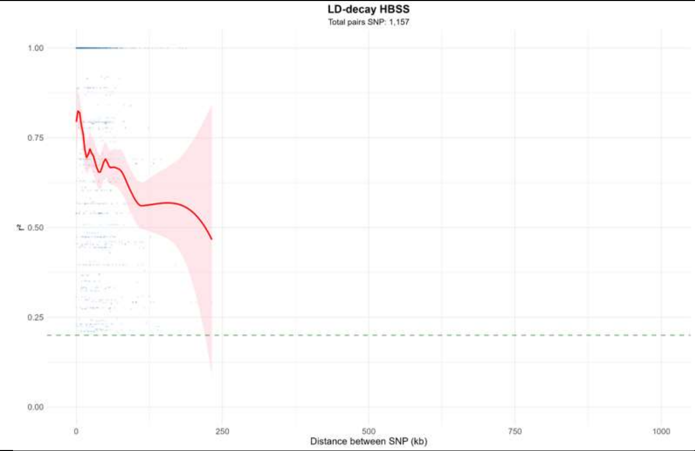

PWB: GGA1

PWB: GGA2

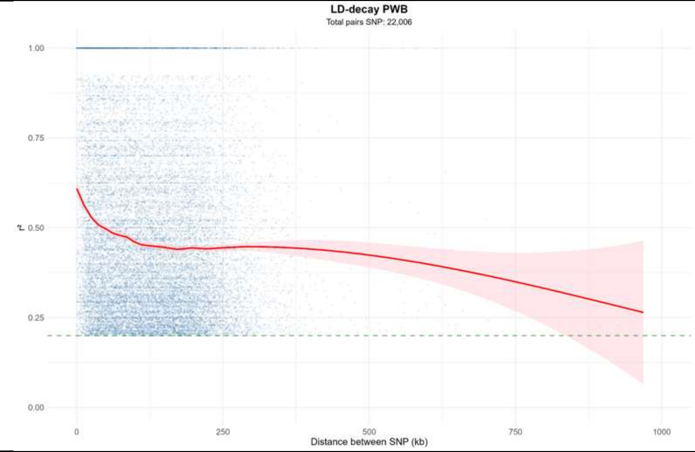

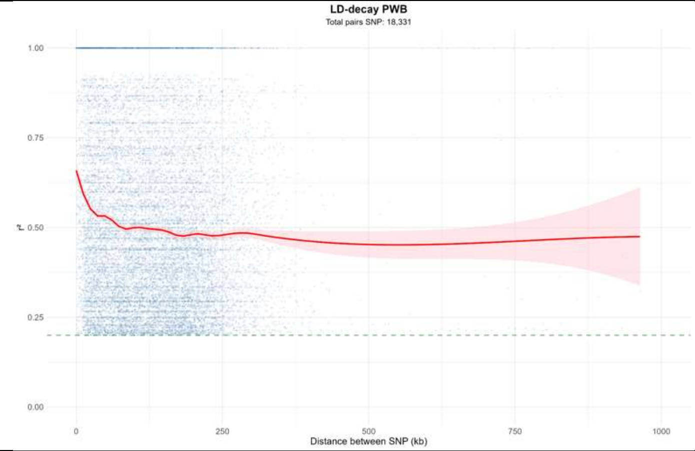

PWB: GGA3

PWB: GGA4

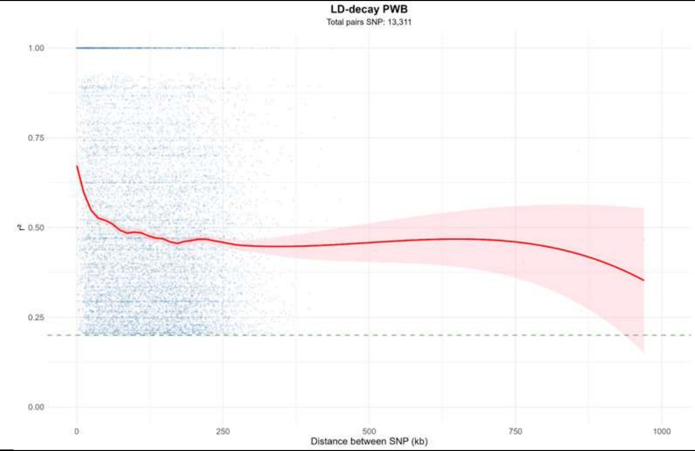

PWB: GGA5

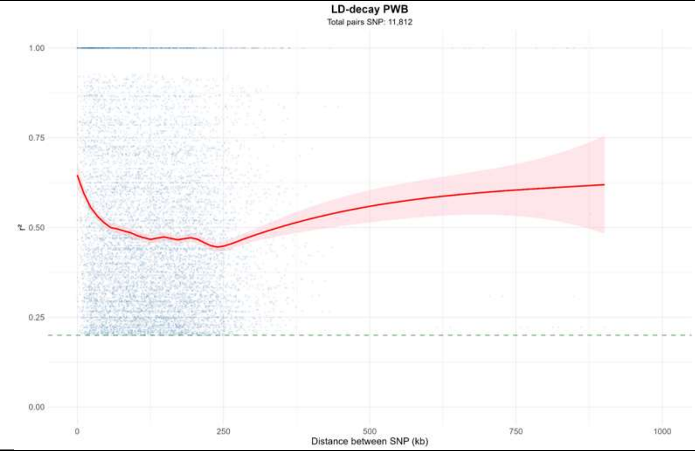

PWB: GGA6

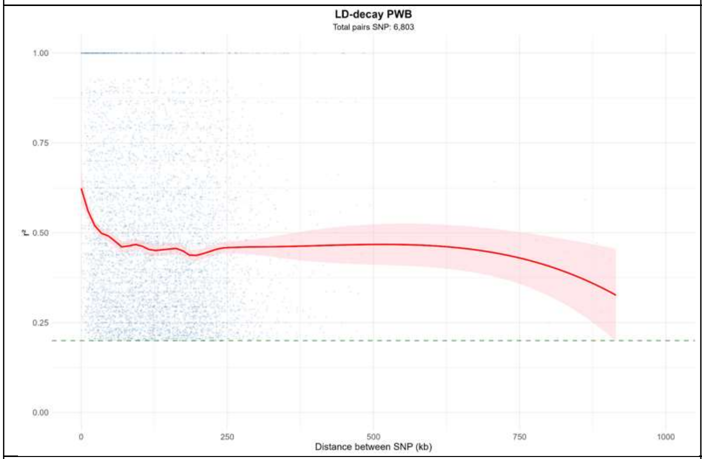

PWB: GGA7

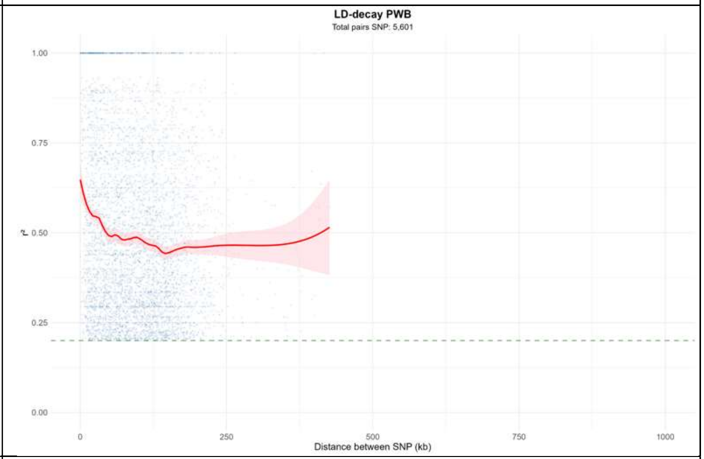

PWB: GGA8

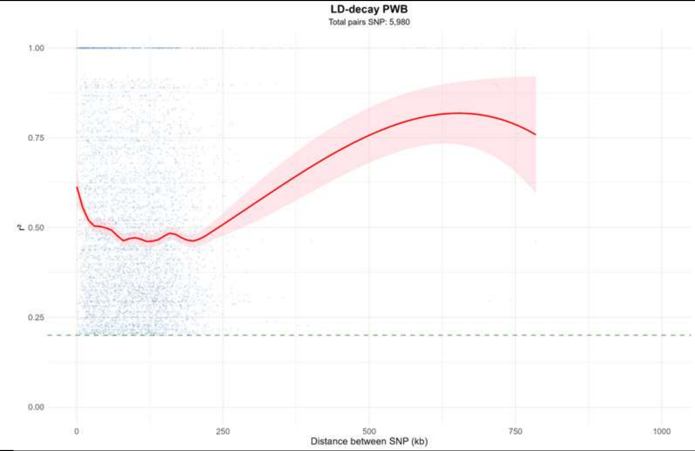

PWB: GGA9

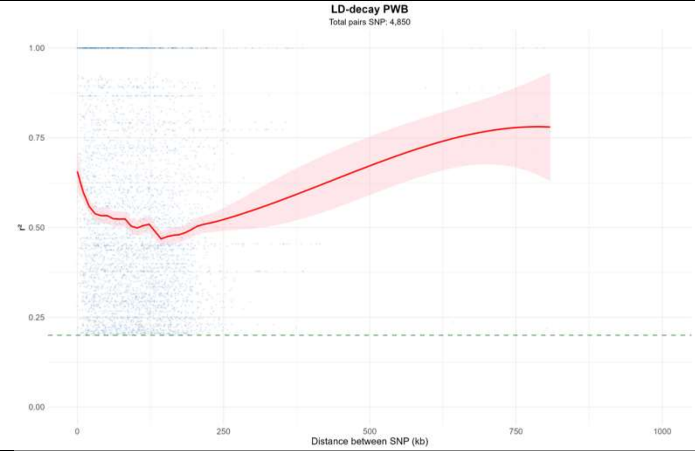

PWB: GGA10

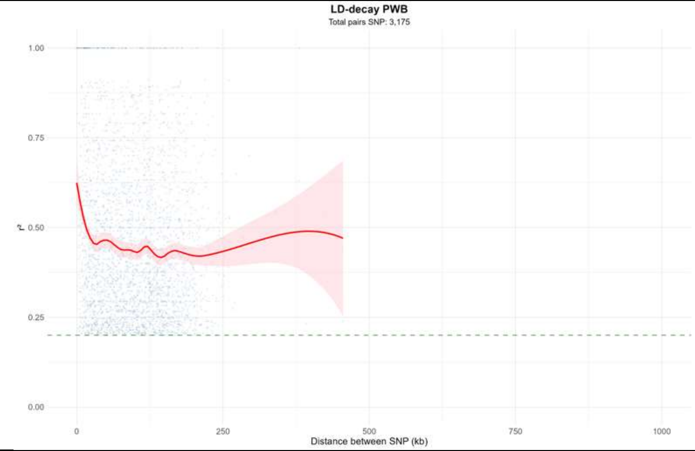

PWB: GGA11

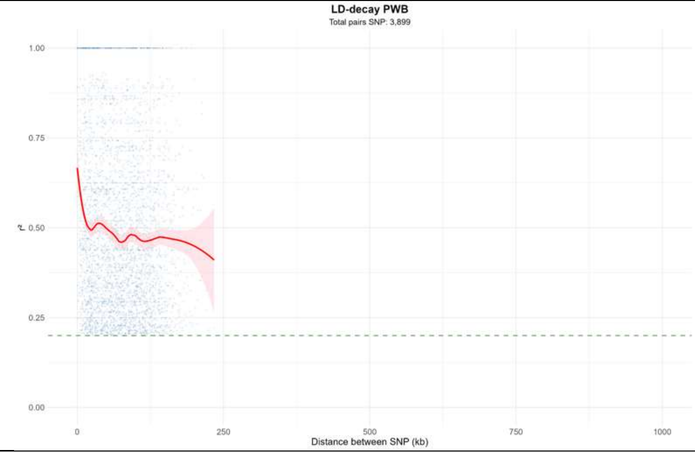

PWB: GGA12

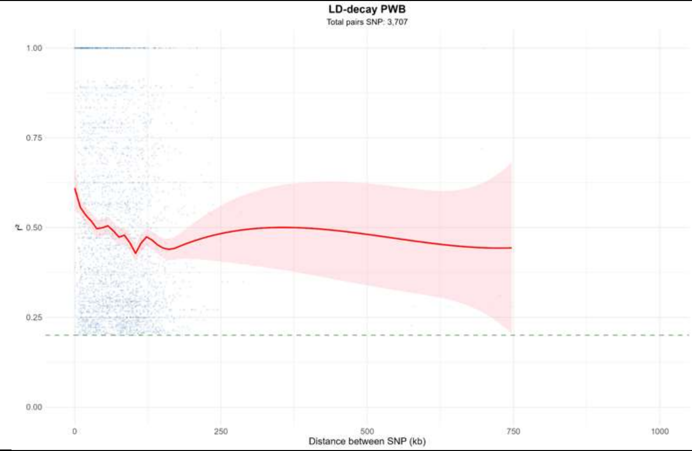

PWB: GGA13

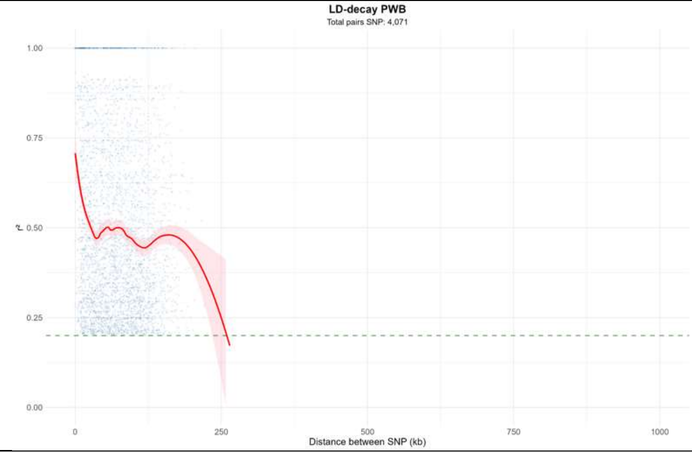

PWB: GGA14

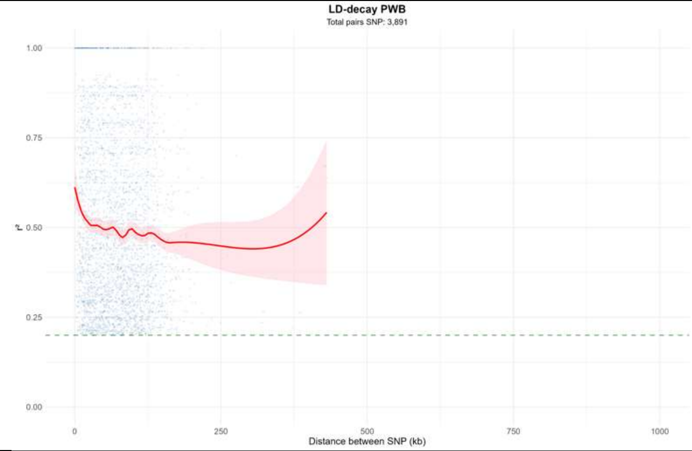

PWB: GGA15

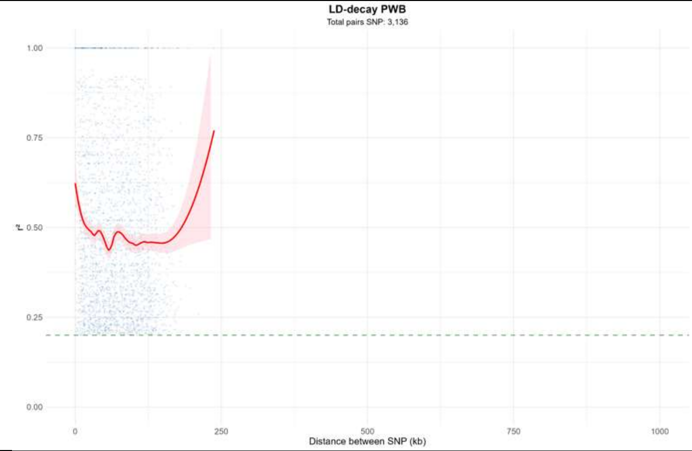

PWB: GGA16

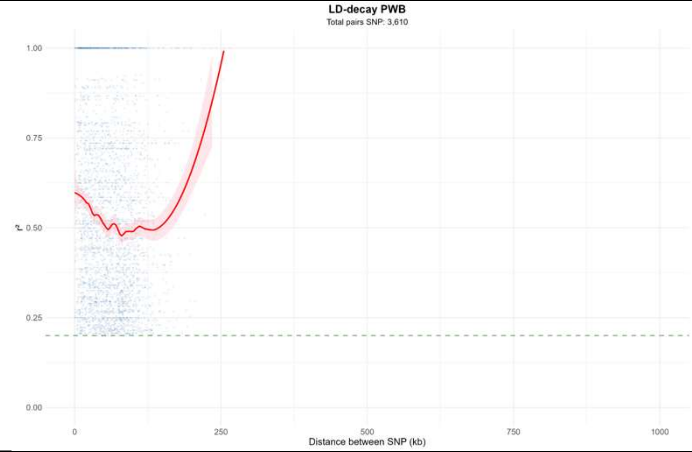

PWB: GGA17

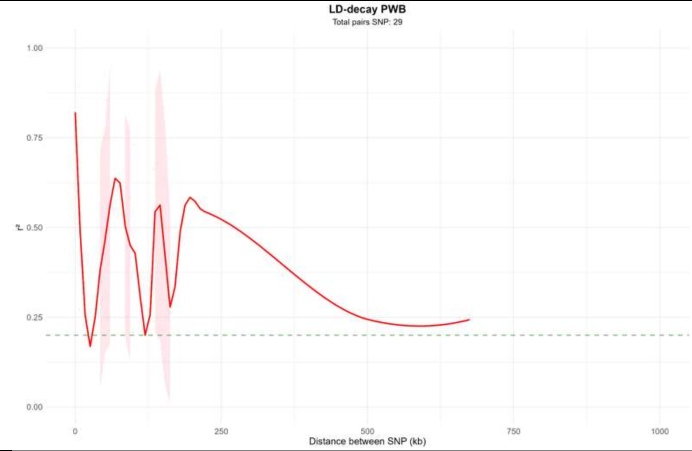

PWB: GGA18

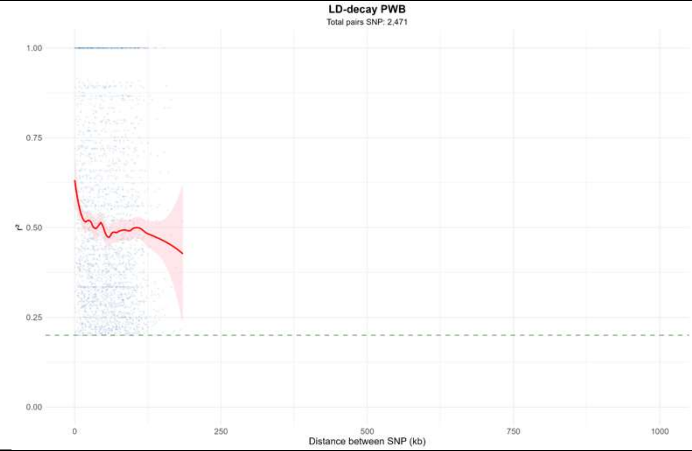

PWB: GGA19

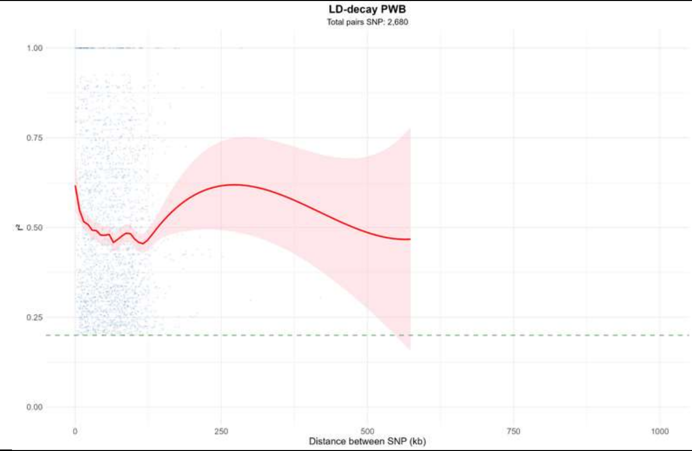

PWB: GGA20

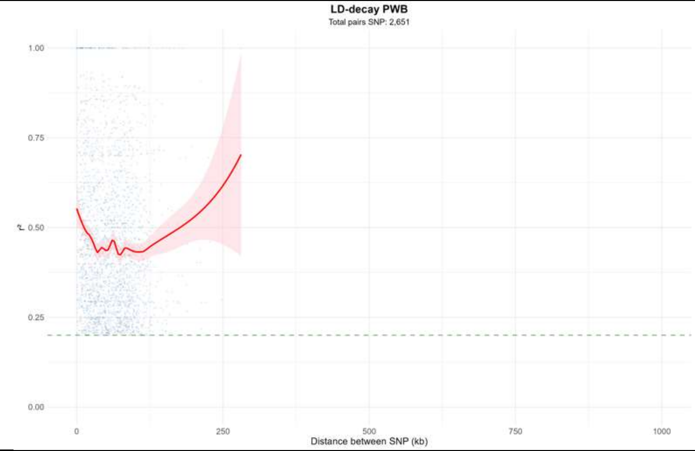

PWB: GGA21

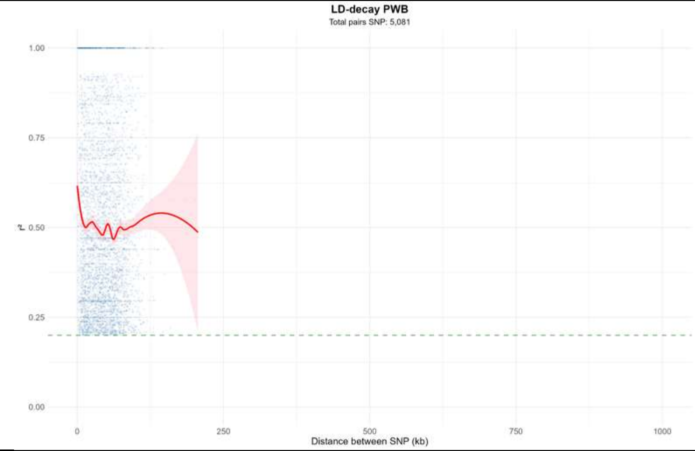

PWB: GGA22

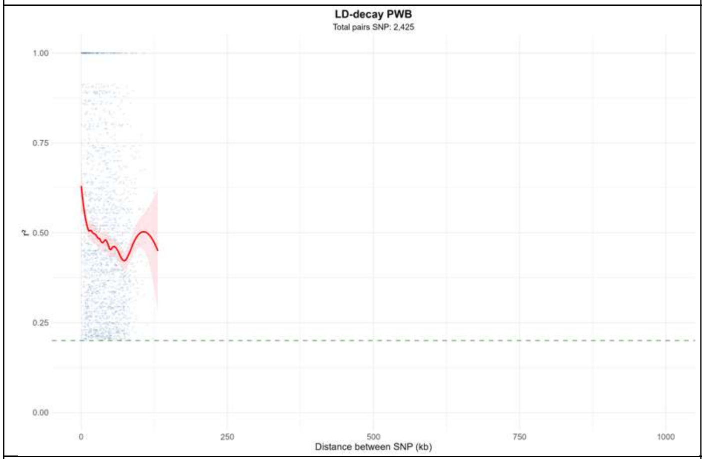

PWB: GGA23

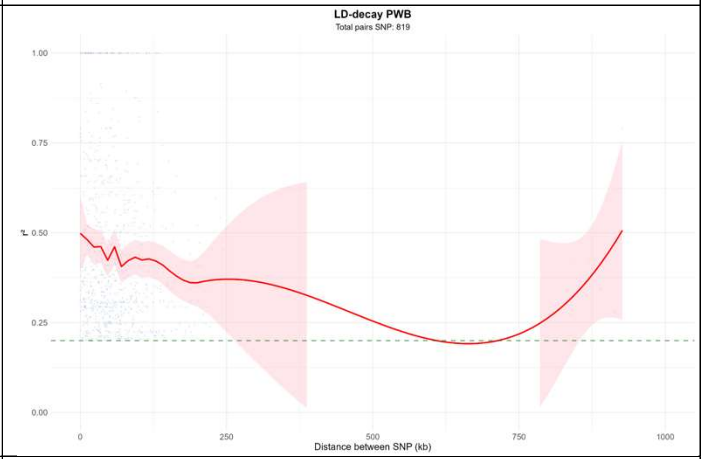

PWB: GGA24

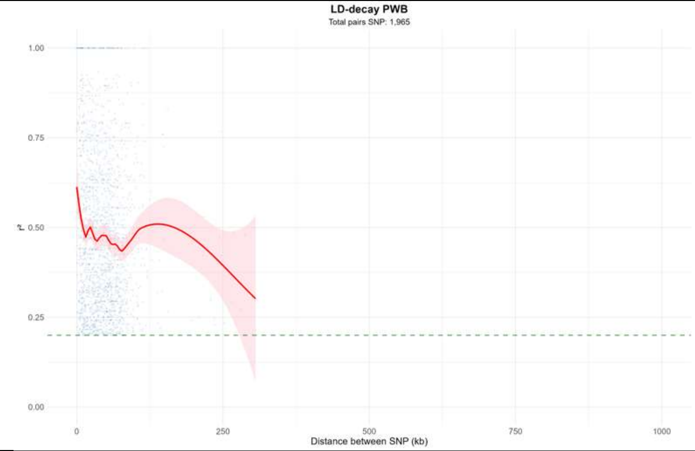

PWB: GGA25

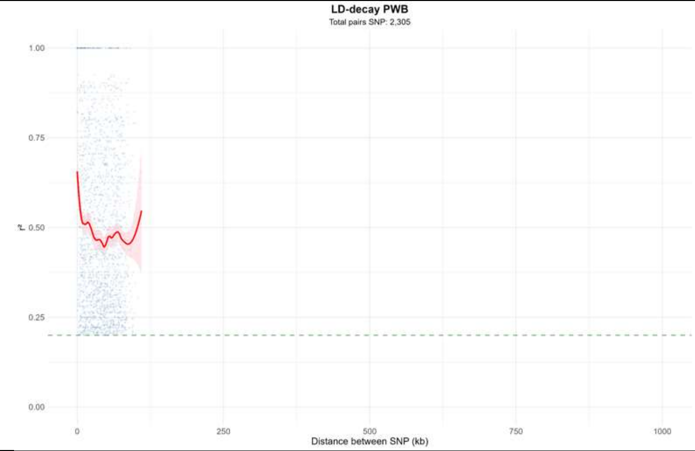

PWB: GGA26

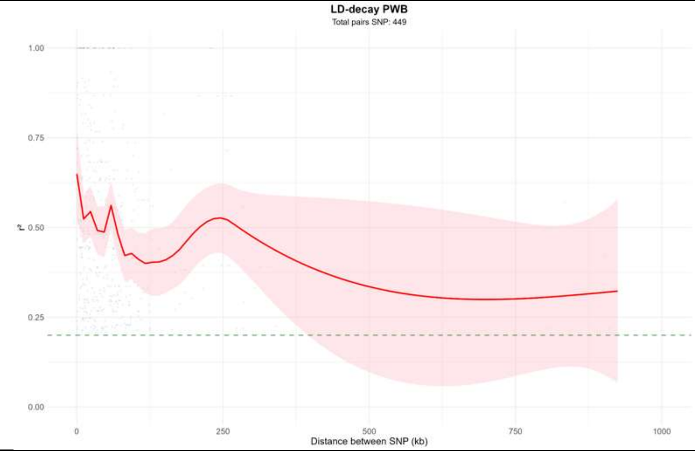

PWB: GGA27

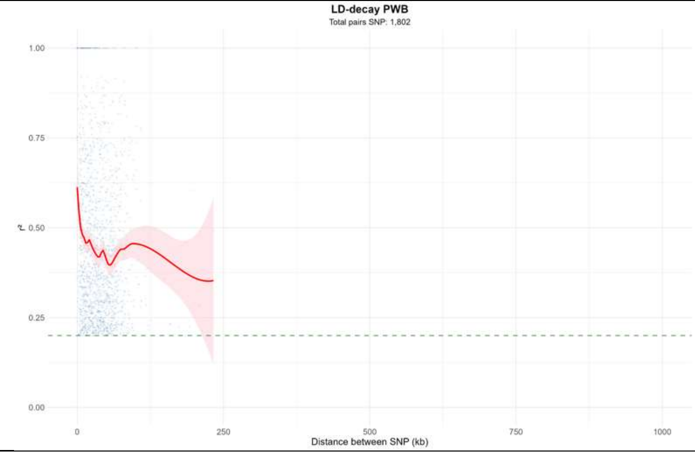

PWB: GGA28

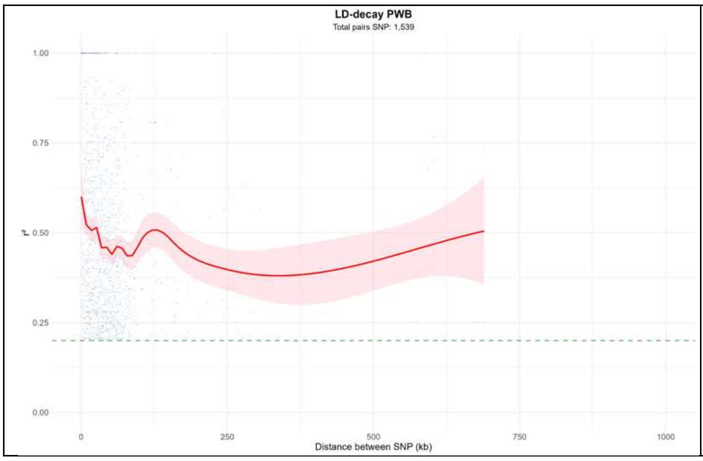

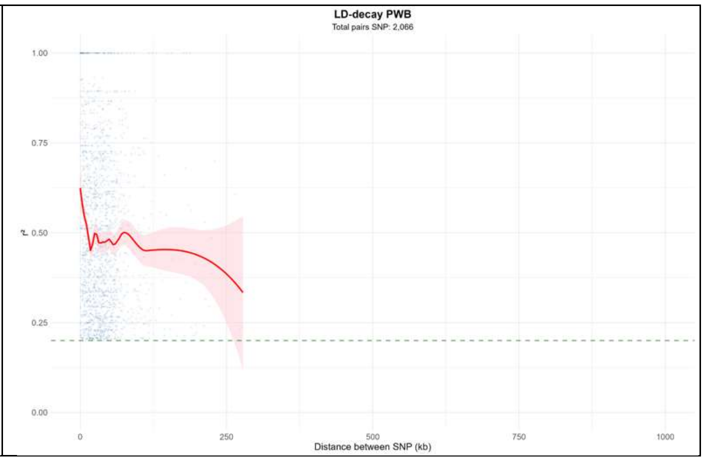

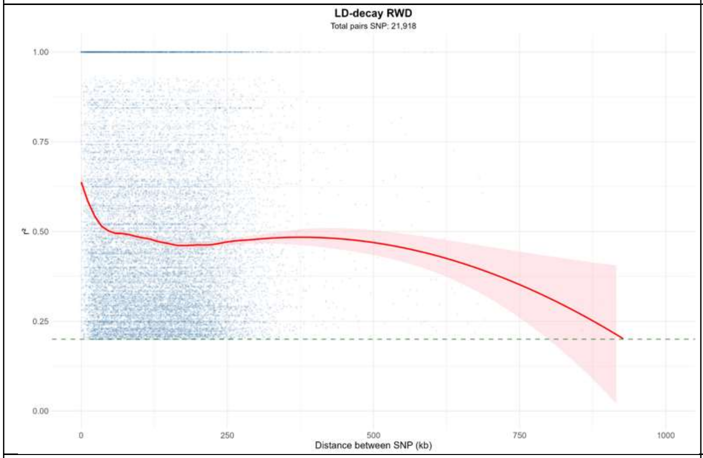

RWD: GGA1

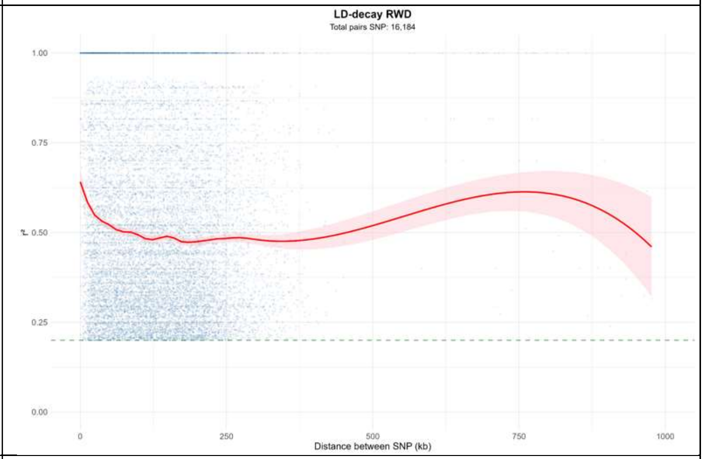

RWD: GGA2

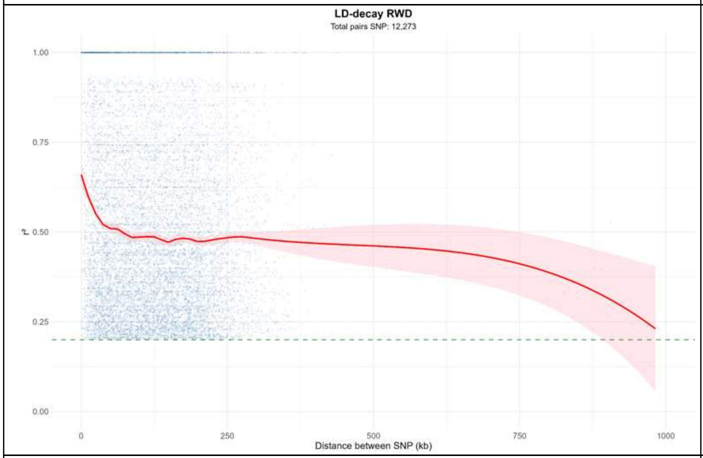

RWD: GGA3

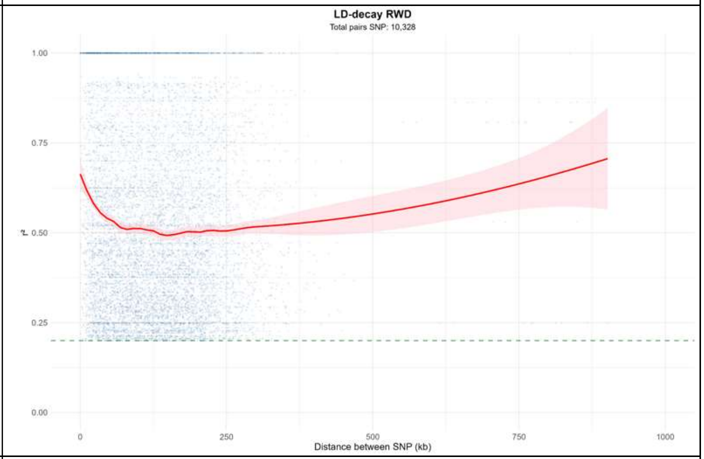

RWD: GGA4

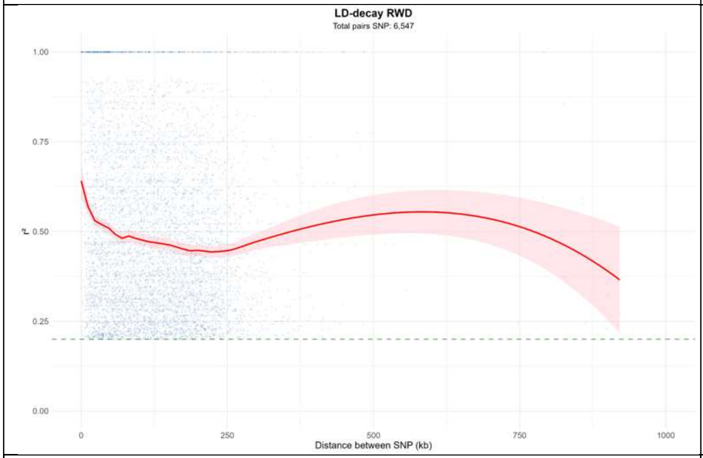

RWD: GGA5

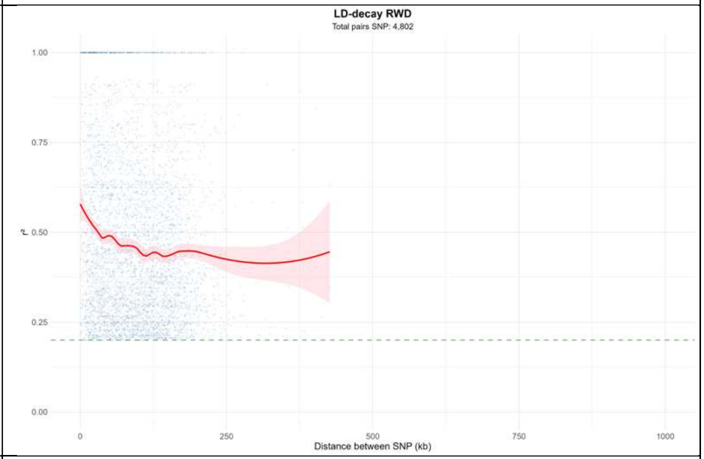

RWD: GGA6

RWD: GGA7

RWD: GGA8

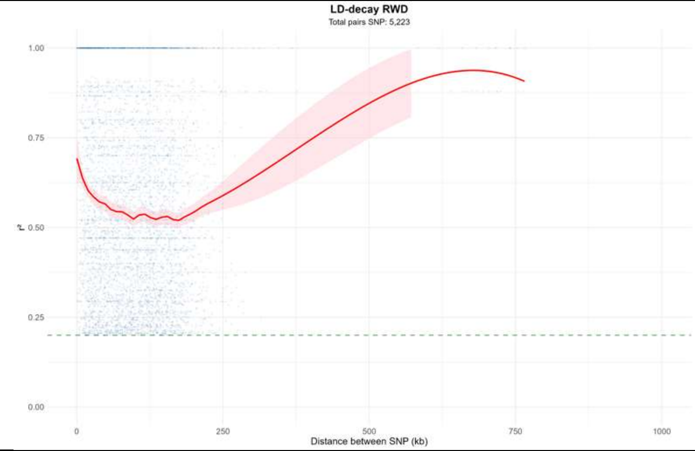

RWD: GGA9

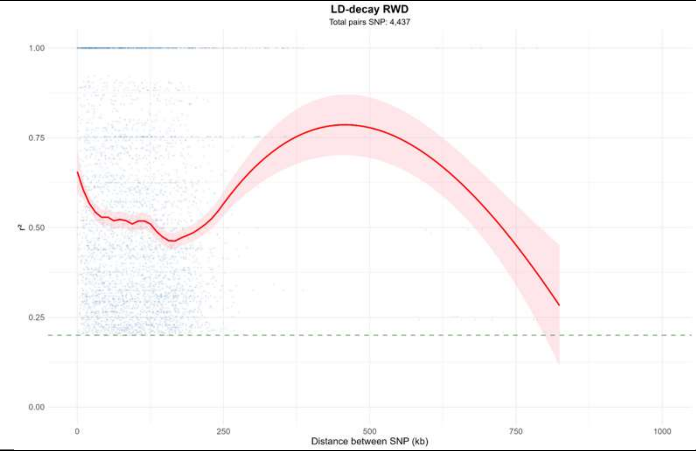

RWD: GGA10

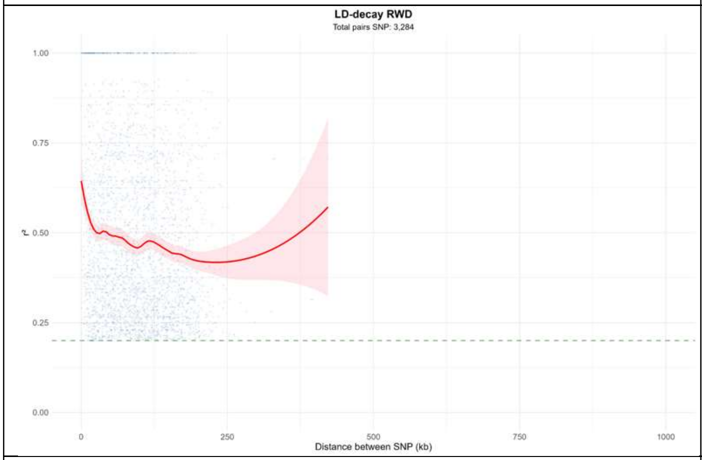

RWD: GGA11

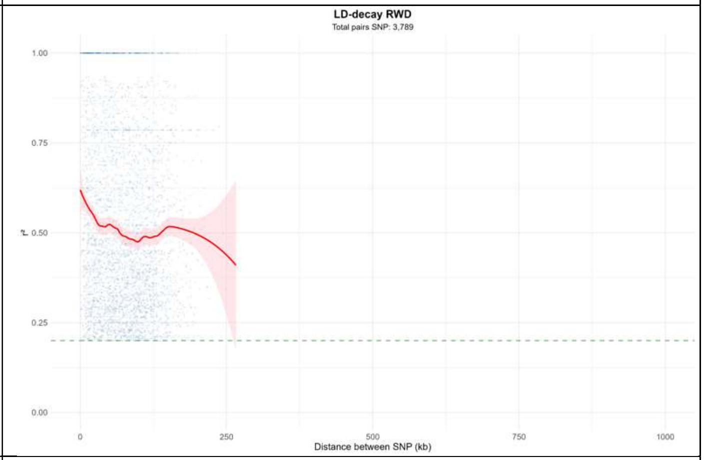

RWD: GGA12

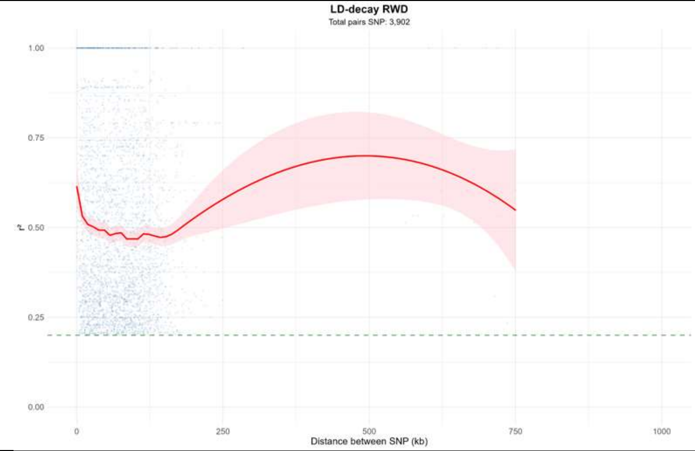

RWD: GGA13

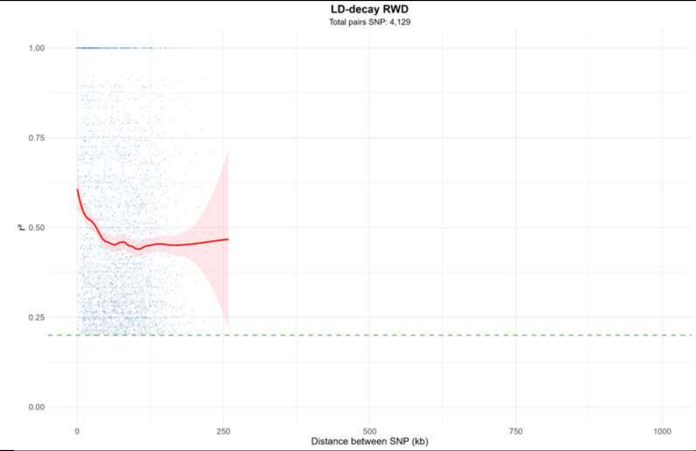

RWD: GGA14

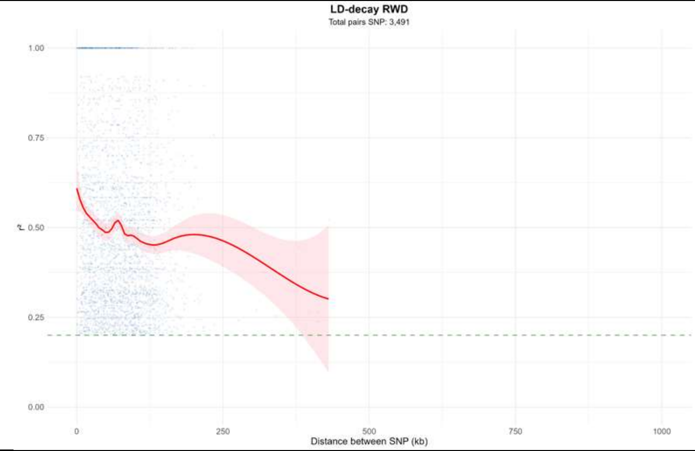

RWD: GGA15

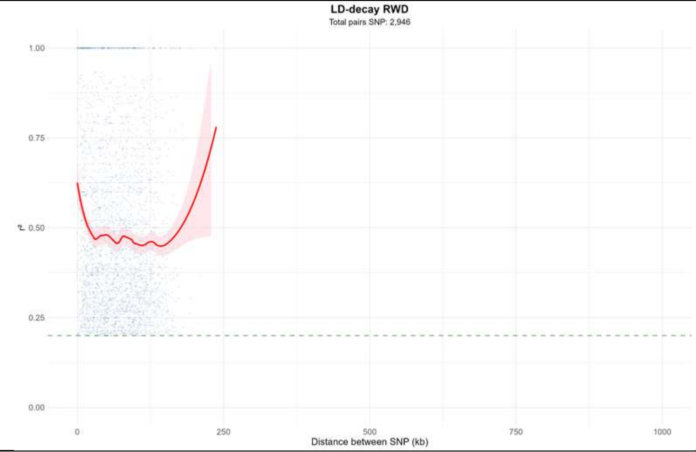

RWD: GGA16

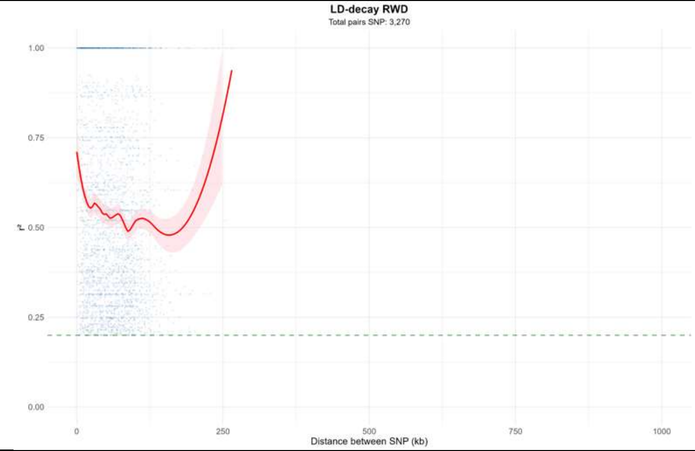

RWD: GGA17

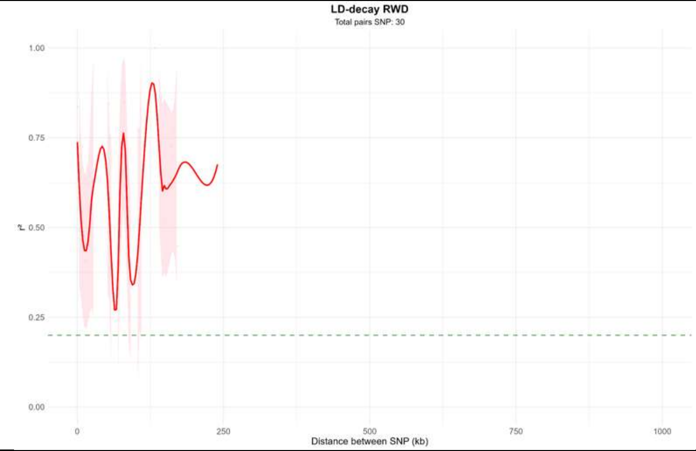

RWD: GGA18

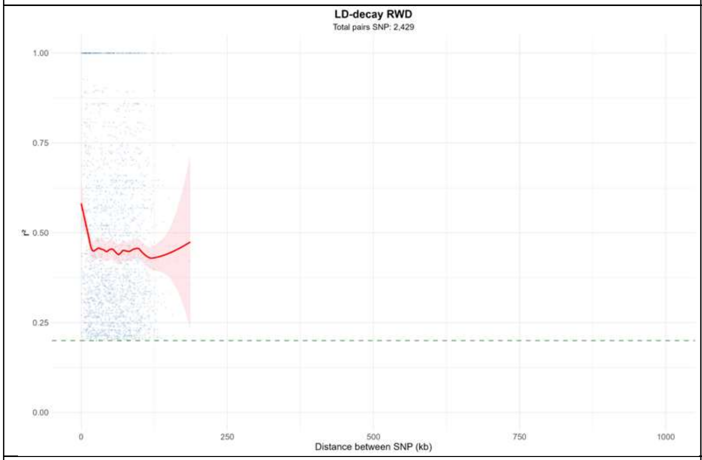

RWD: GGA19

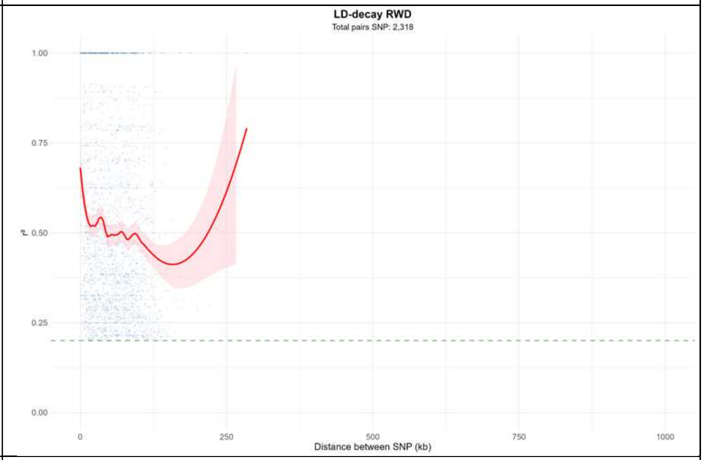

RWD: GGA20

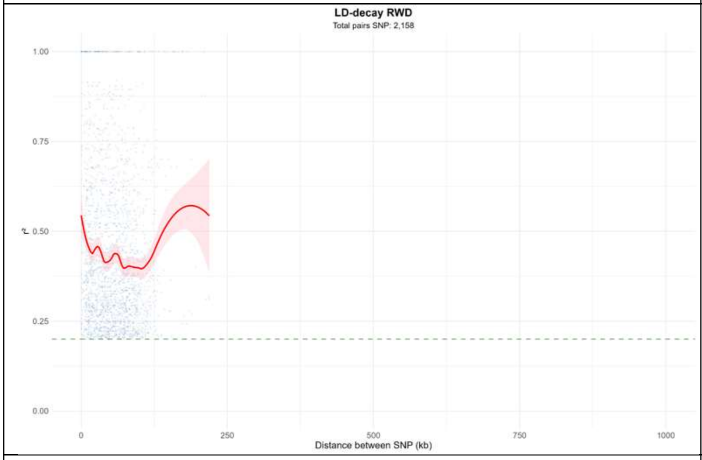

RWD: GGA21

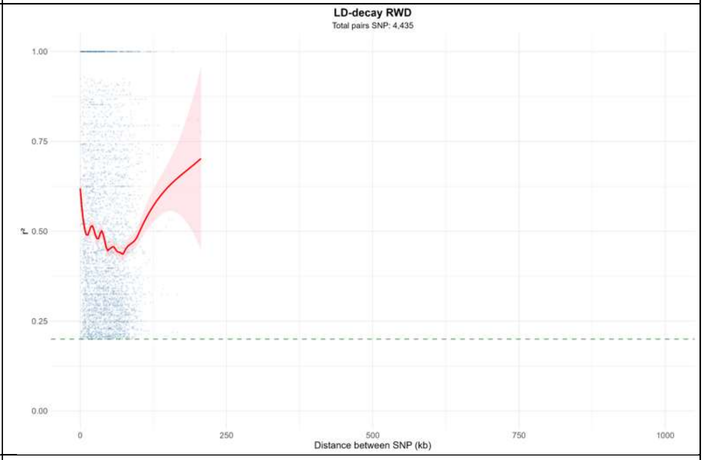

RWD: GGA22

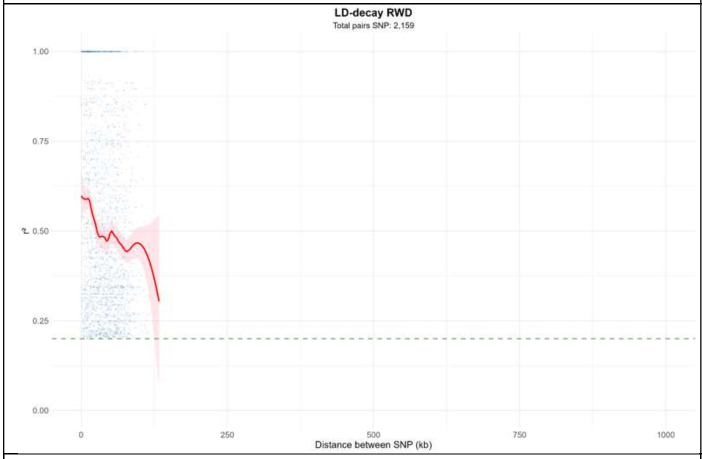

RWD: GGA23

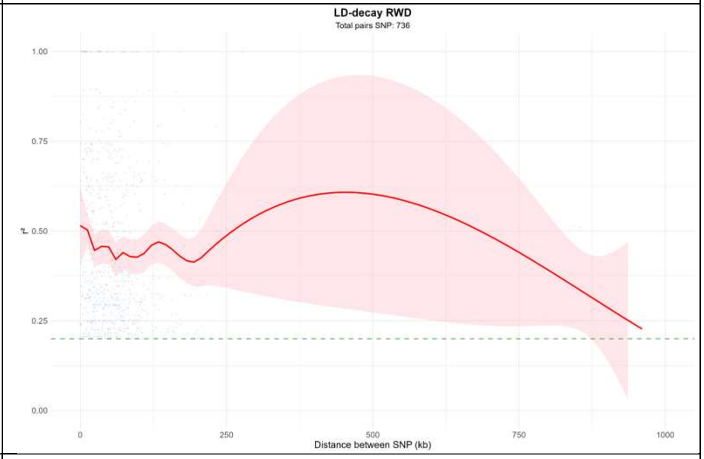

RWD: GGA24

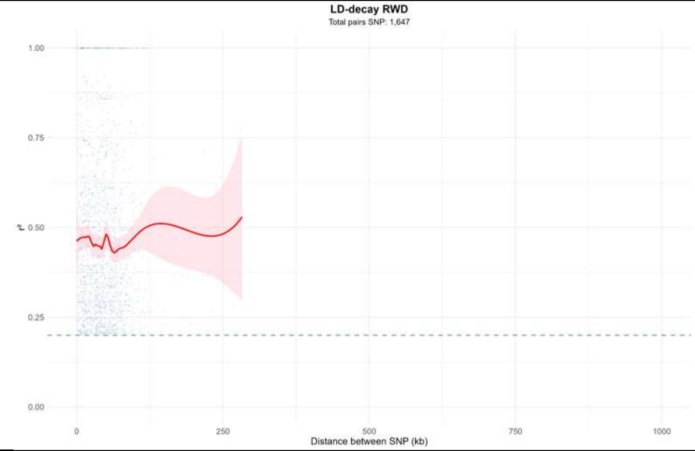

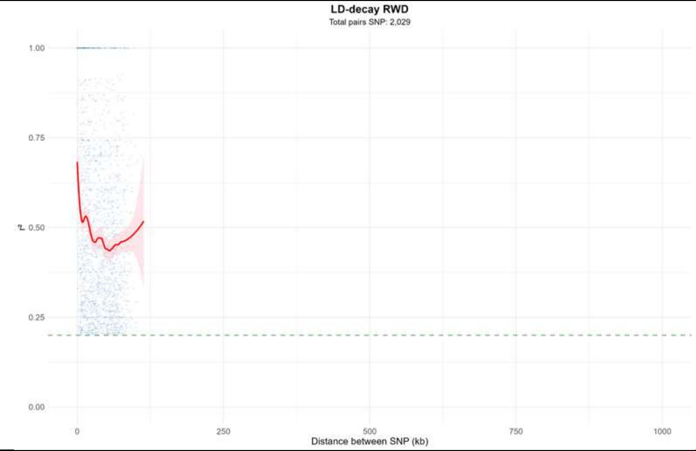

RWD: GGA25	RWD: GGA26

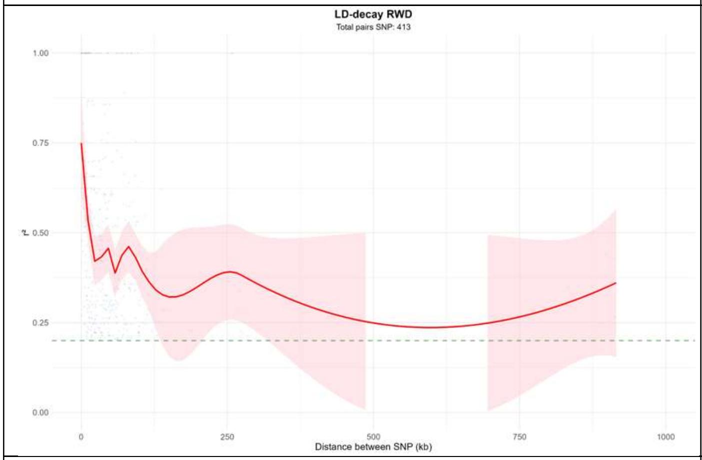

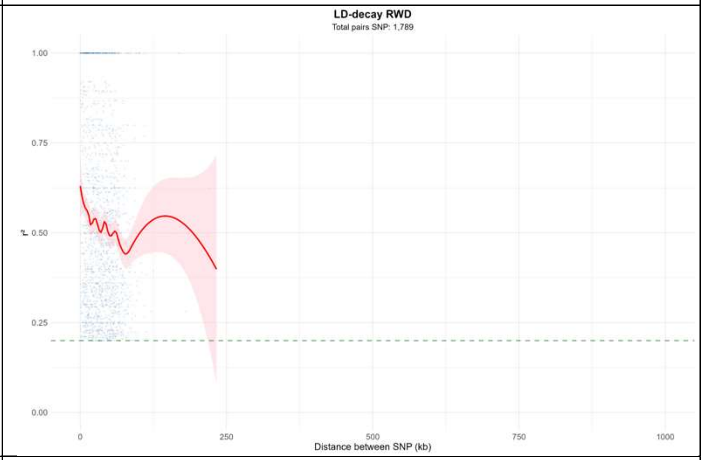

RWD: GGA27	RWD: GGA28

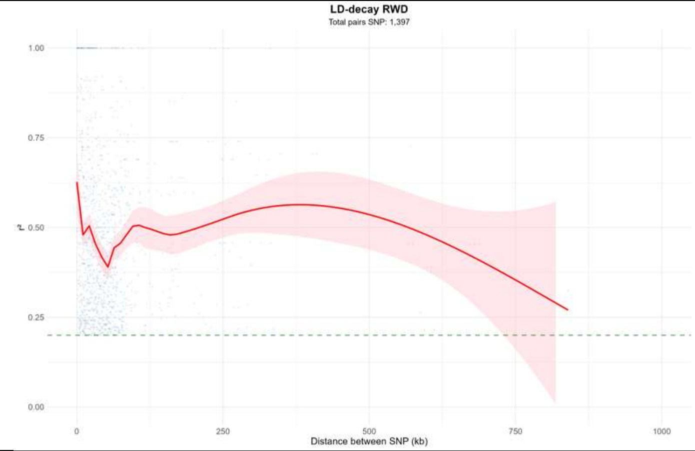

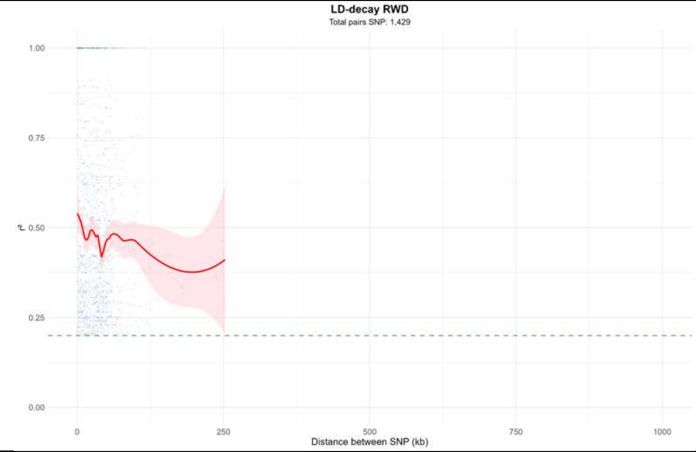

SW: GGA1	SW: GGA2

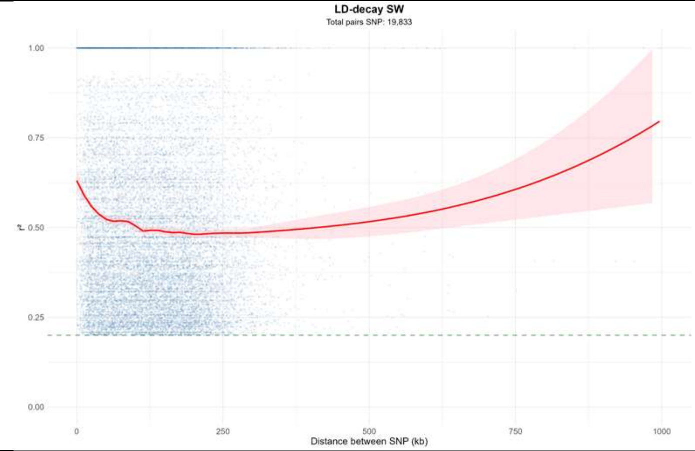

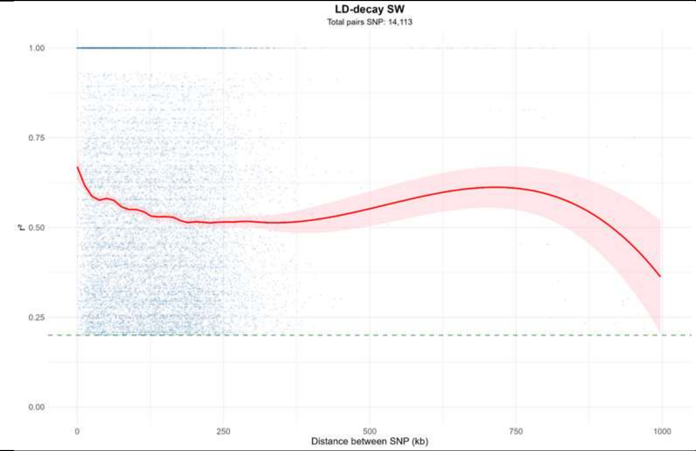

SW: GGA3	SW: GGA4

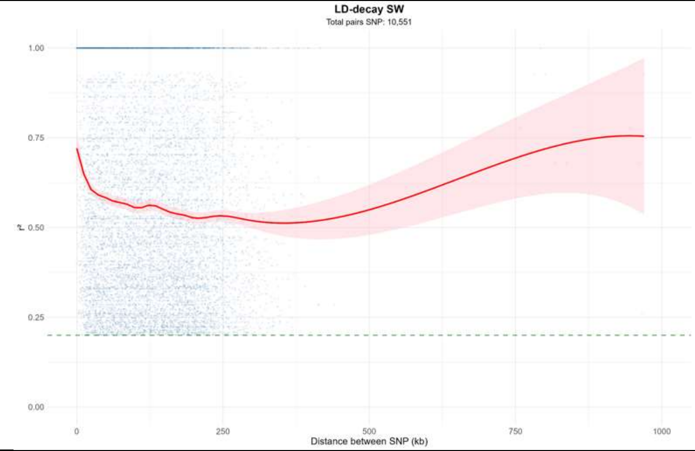

SW: GGA5

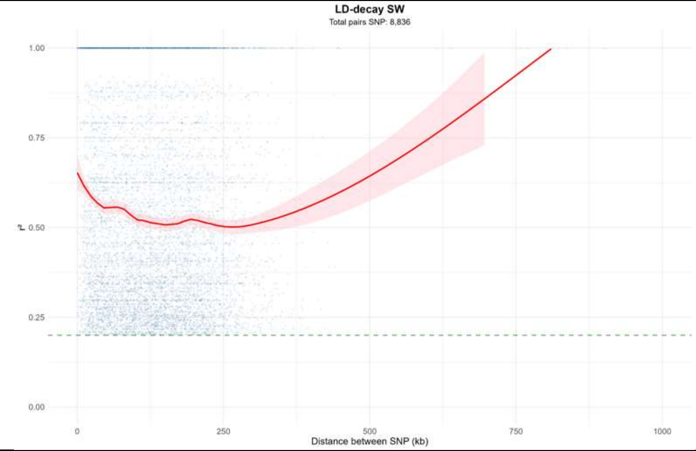

SW: GGA6

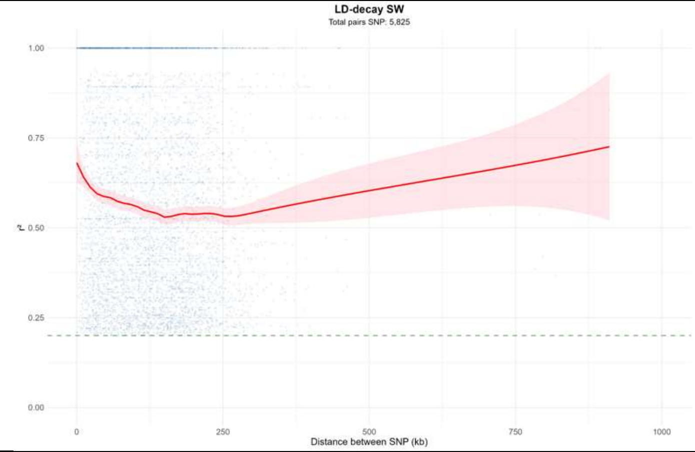

SW: GGA7

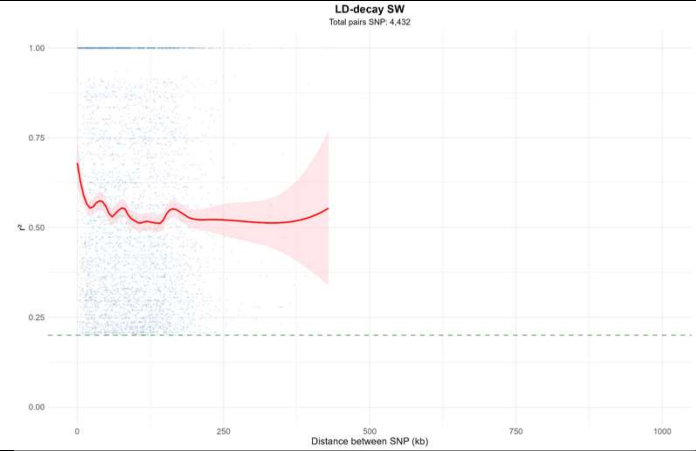

SW: GGA8

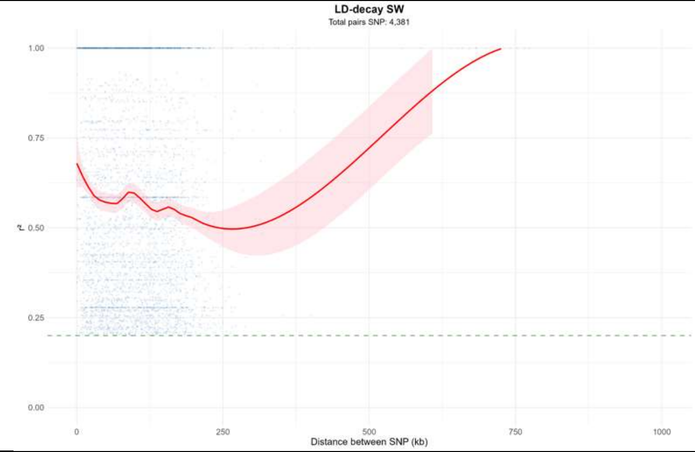

SW: GGA9

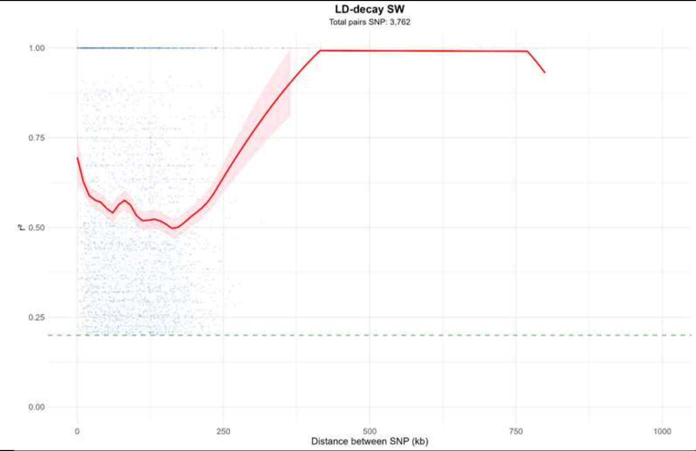

SW: GGA10

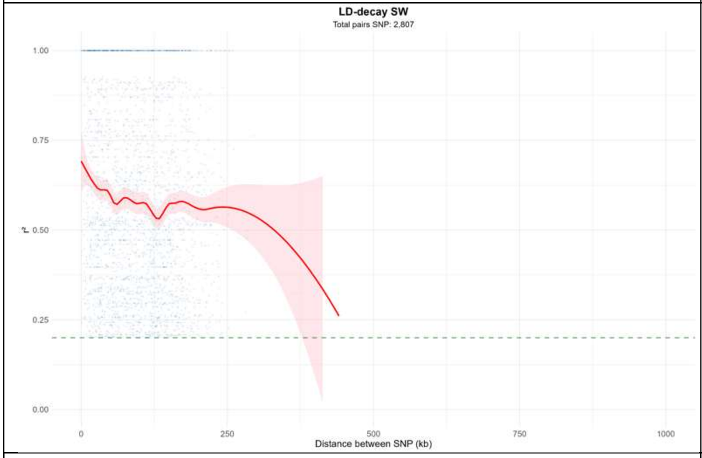

SW: GGA11

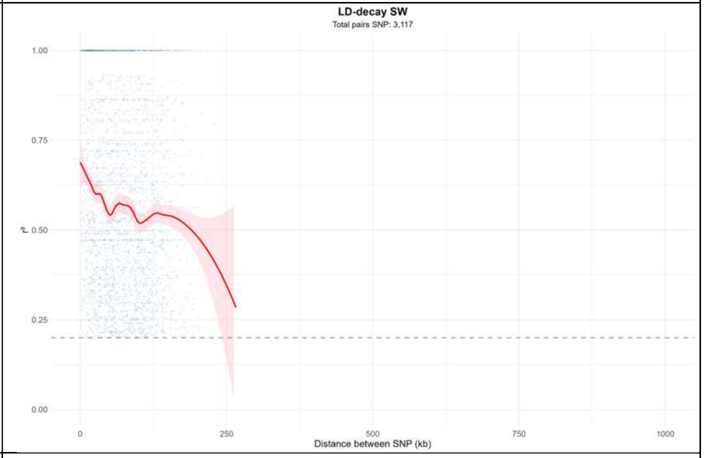

SW: GGA12

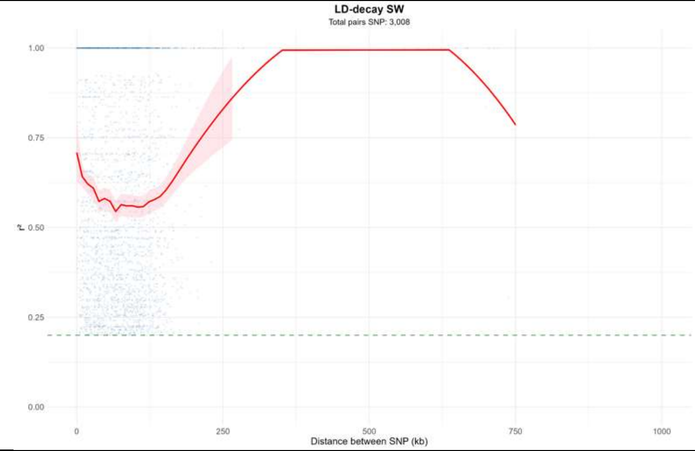

SW: GGA13

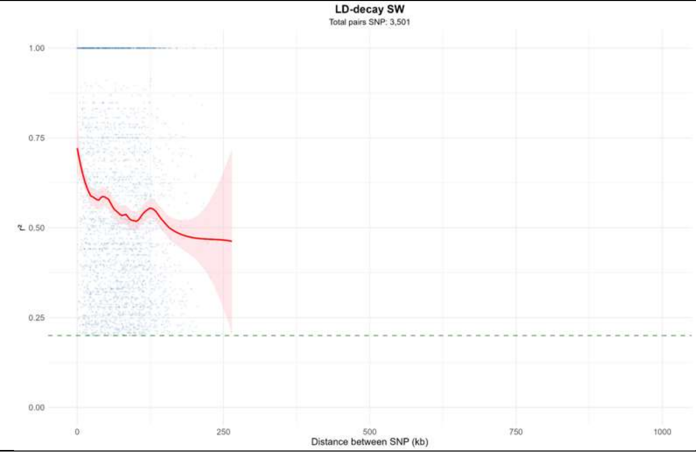

SW: GGA14

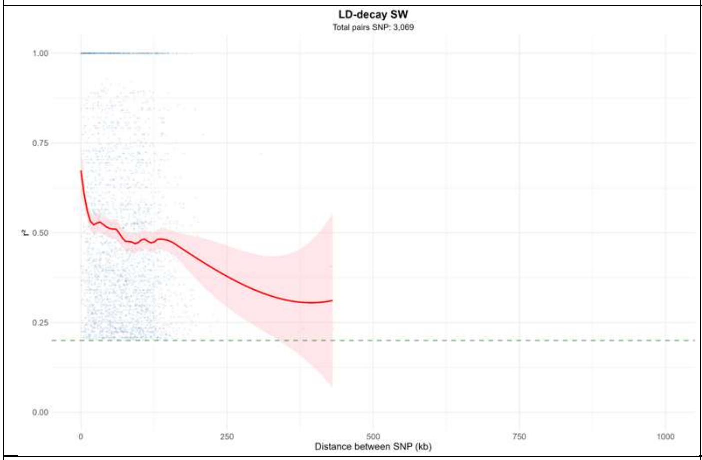

SW: GGA15

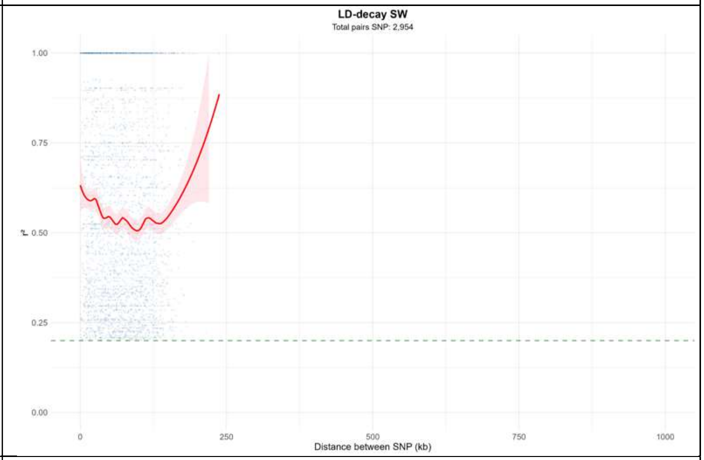

SW: GGA16

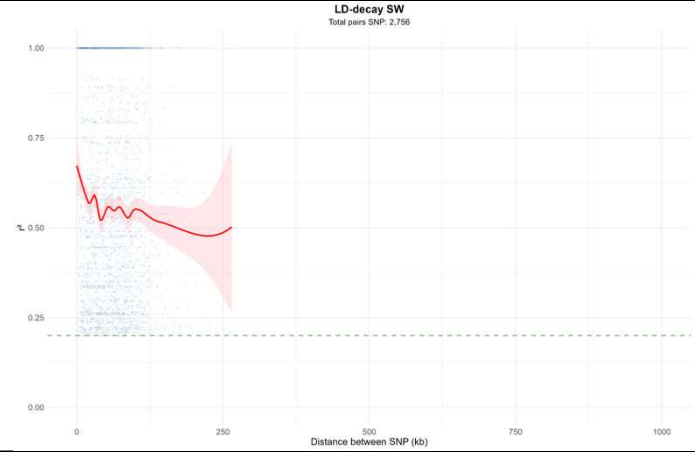

SW: GGA17

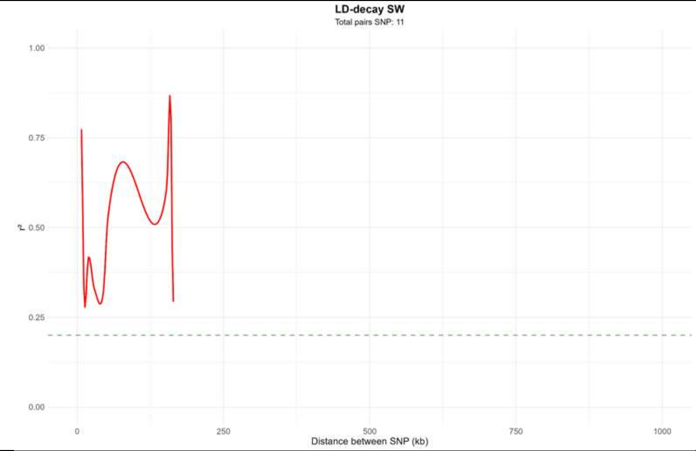

SW: GGA18

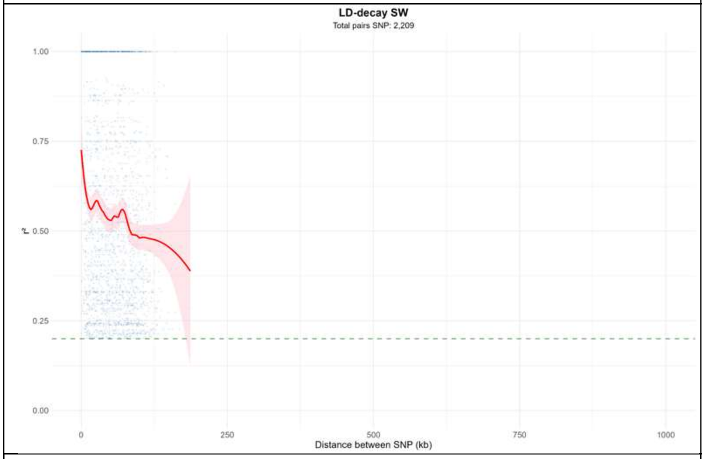

SW: GGA19

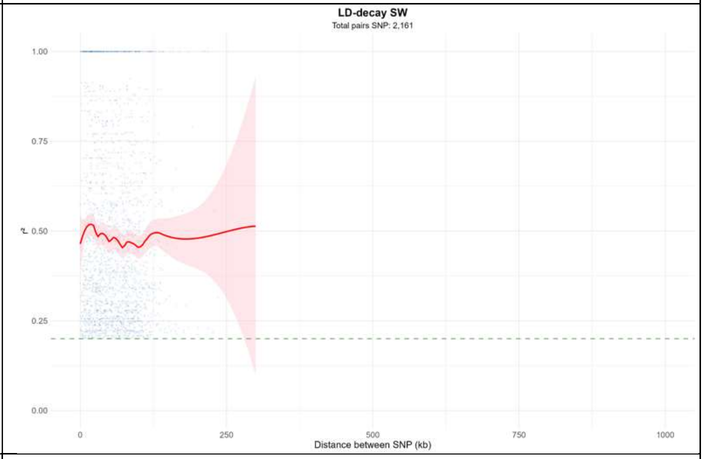

SW: GGA20

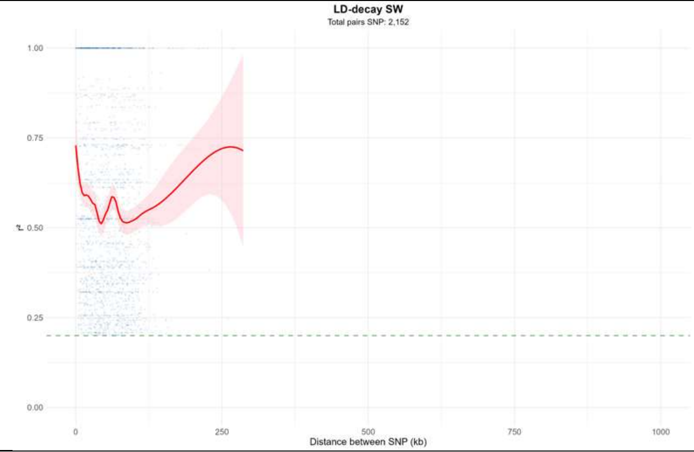

SW: GGA21

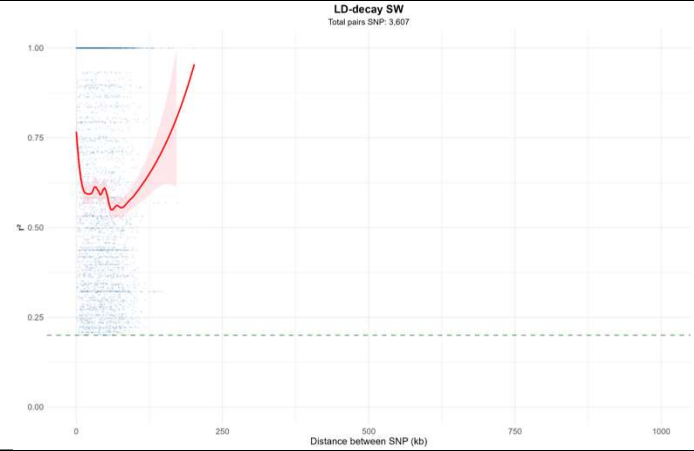

SW: GGA22

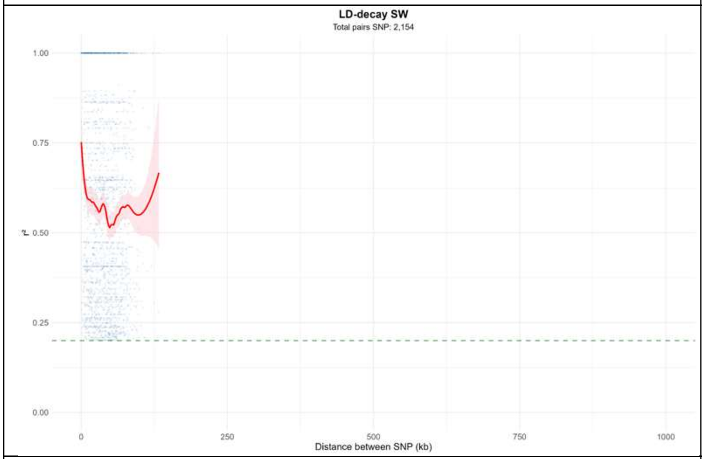

SW: GGA23

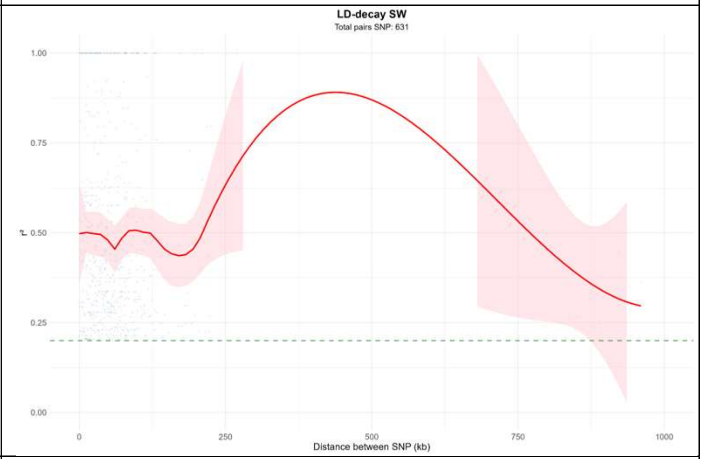

SW: GGA24

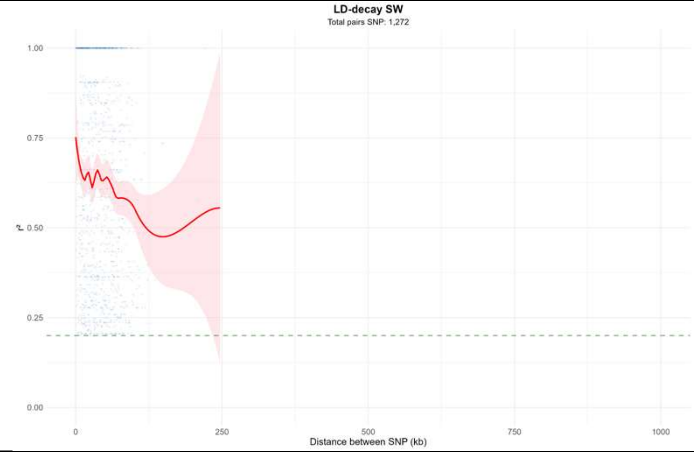

SW: GGA25

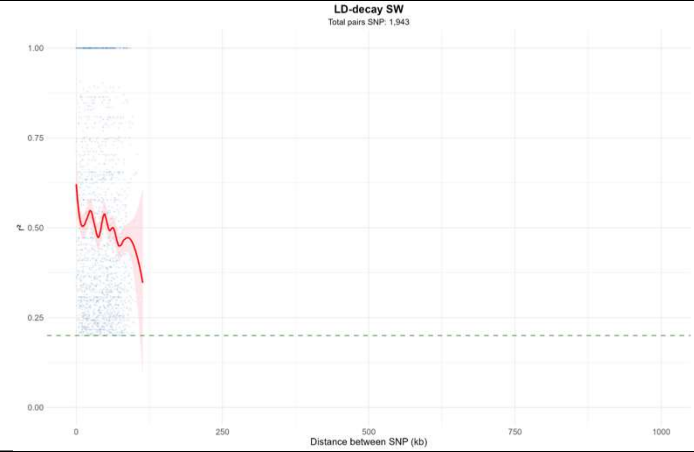

SW: GGA26

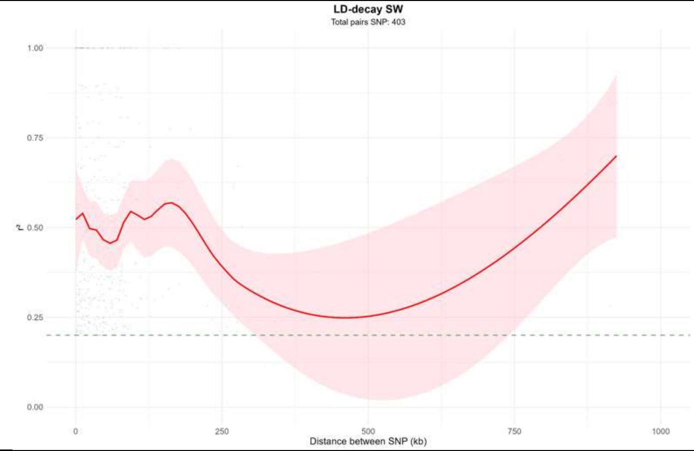

SW: GGA27

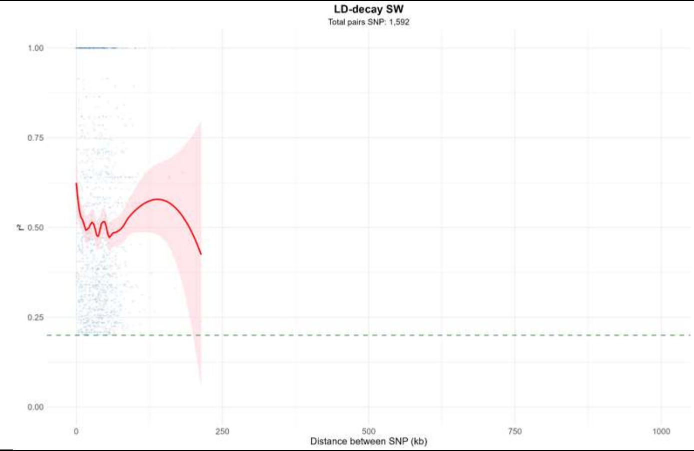

SW: GGA28

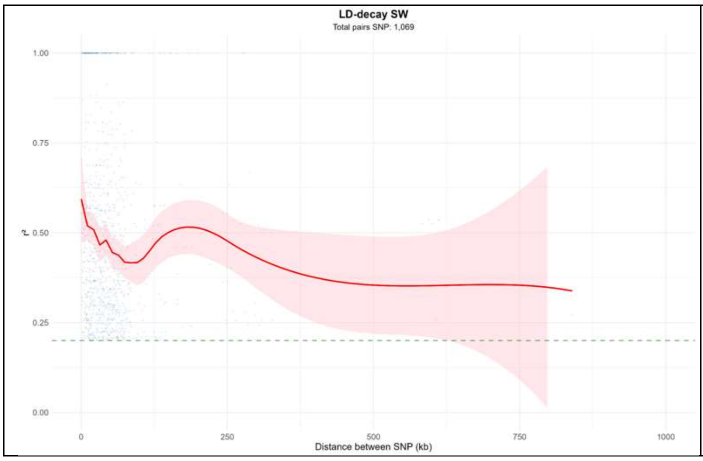

LD-decay SW
Total pairs SNP: 1,069
1.00
0.75
r² 0.50
0.25
0.00
0
250
500
750
1000
Distance between SNP (kb)

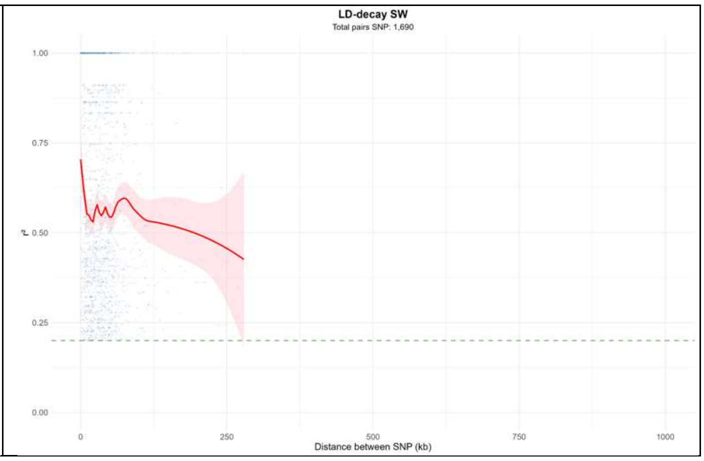

LD-decay SW
Total pairs SNP: 1,690
1.00
0.75
r² 0.50
0.25
0.00
0
250
500
750
1000
Distance between SNP (kb)
