# Supplementary material for: Kinglet in the Poultry Court of Russia: Whole-Genome Insights into Ancestry, Genetic Variability, Selection Footprints and Candidate Genes in a Unique Local Chicken Breed Relative to Other Bantam/Dwarf Breeds
Source: Animals (Basel). 2026 Feb 17;16(4):642. doi: 10.3390/ani16040642 (PMC12937304; doi:10.3390/ani16040642)
Supplement: Supplementary file 1 [file animals-16-00642-s001.zip › Supplementary Figures S1 and S2.pdf]

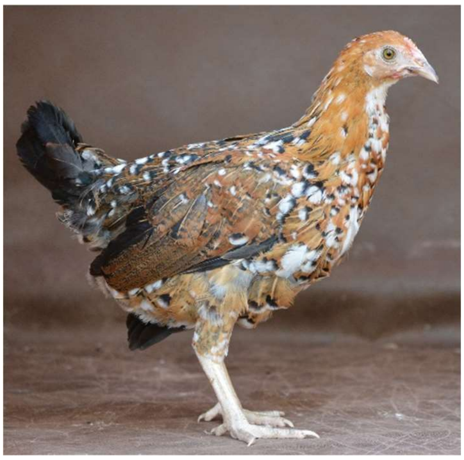

(**a**)

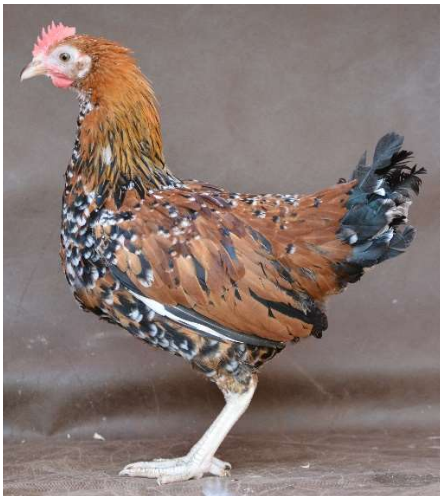

(**b**)

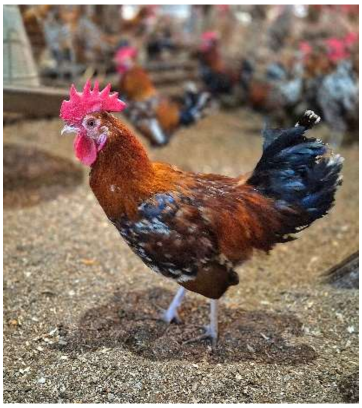

(**c**)

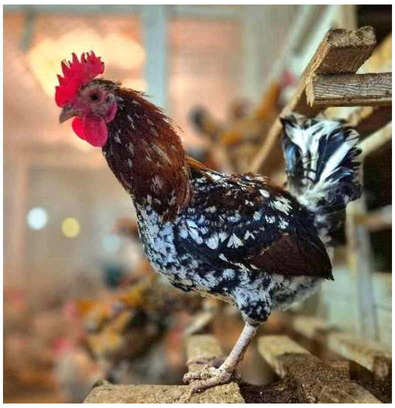

(**d**)

Supplementary Figure S1. Variations in plumage coloration patterns and other phenotypic characteristics of hens (**a**) and roosters (**b**, **c**, **d**) of the Russian Korolyok breed.

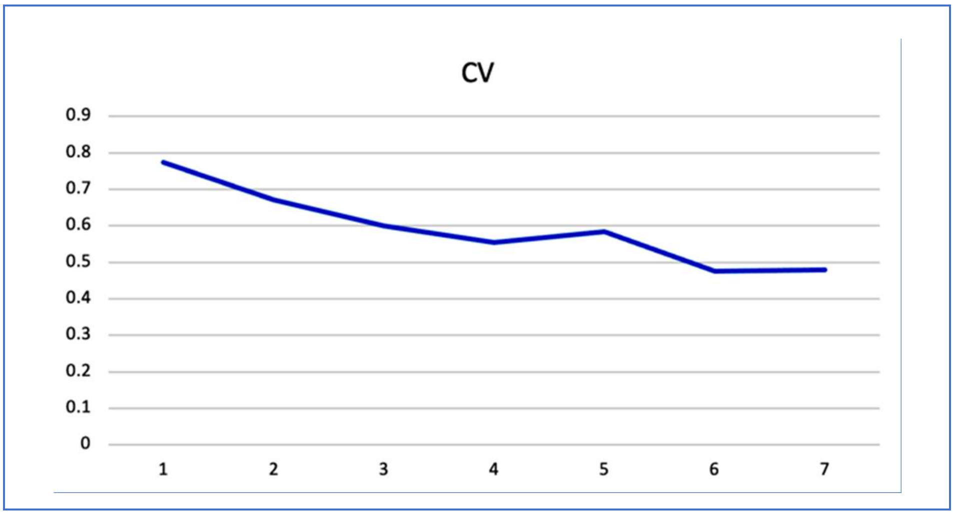

Supplementary Figure S2. Cross-validation (CV) error plot showing that the optimal number of ancestral populations was K = 6.
