# Supplementary material for: Kinglet in the Poultry Court of Russia: Whole-Genome Insights into Ancestry, Genetic Variability, Selection Footprints and Candidate Genes in a Unique Local Chicken Breed Relative to Other Bantam/Dwarf Breeds
Source: Animals (Basel). 2026 Feb 17;16(4):642. doi: 10.3390/ani16040642 (PMC12937304; doi:10.3390/ani16040642)
Supplement: Supplementary file 1 [file animals-16-00642-s001.zip › Supplementary Information Box S1.pdf]

Supplementary Information Box S1

Russian Dwarf (Korolyok) Chicken Breed [47]

This is a local ornamental and utility chicken breed that originated in Russia. It is characterized by high adaptability, hardiness, unpretentious management requirements, and a strongly expressed brooding instinct. The breed is mainly maintained in smallholder and hobby farms, where it is valued for its vitality, calm temperament, and attractive appearance.

Adult males weigh 0.8–0.9 kg and females 0.7–0.8 kg. Annual egg production averages 80–90 white-shelled eggs with a minimum egg weight of 30–35 g. The brooding instinct is well developed, making the breed suitable for natural reproduction.

Males have a compact, slightly elevated body, a broad breast, and a high, well-developed tail with long curved sickle feathers. The head is small and rounded, with a single or rose-type comb, a short dark beak, and red wattles and earlobes. Wings are broad and tightly fitting to the body; shanks are short, smooth, and unfeathered. Females share the same general conformation but are smaller, with a lower-set body and a compact tail; the comb may be slightly inclined to one side.

The plumage is dense and closely fitting. The most common color varieties are black-breasted red and brown-red. In the black-breasted red type, males exhibit golden-red plumage on the head and hackle with a glossy black breast and tail showing greenish sheen, while females display light brown plumage with salmon-red breast and darker tail. Brown-red birds have deeper reddish tones in males and warm brown shades in females.

Major disqualifying traits include an elongated body, weak or poorly set tail, atypical comb shape, feathered shanks, and dull or mottled plumage.

The Russian Dwarf represents a stable local genetic resource combining ornamental appearance with strong reproductive traits, making it valuable for conservation and small-scale breeding programs.
