# Supplementary material for: Kinglet in the Poultry Court of Russia: Whole-Genome Insights into Ancestry, Genetic Variability, Selection Footprints and Candidate Genes in a Unique Local Chicken Breed Relative to Other Bantam/Dwarf Breeds
Source: Animals (Basel). 2026 Feb 17;16(4):642. doi: 10.3390/ani16040642 (PMC12937304; doi:10.3390/ani16040642)
Supplement: Supplementary file 1 [file animals-16-00642-s001.zip › Supplementary Table S1.pdf]

Supplementary Table S1. Density of detected SNPs within runs of homozygosity (ROHs) per 1 Mb.

GGA [1]	ROHs	ROH length	Number SNPs within ROH	Density of detected SNPs
1	3,688,051–4,987,231	1,299,180	40	30.79
	52,677,319–55,590,991	2,913,672	100	34.32
	75,548,455–78,805,452	3,256,997	70	21.49
	156,375,568–163,630,641	7,255,073	254	35.01
	189,025,664–191,763,199	2,737,535	98	35.80
2	**80,850,520–83,898,189**	3,047,669	74	24.28
	141,620,636–148,345,259	6,724,623	263	39.11
3	68,715,224–71,511,986	2,796,762	99	35.40
	72,435,078–73,021,083	586,005	21	35.84
	73,910,974–75,134,412	1,223,438	44	35.96
	93,570,116–93,761,038	190,922	8	41.90
4	**8,274,461–8,965,874**	691,413	24	34.71
7	23,231,559–26,225,419	2,993,860	163	54.45
	**26,318,341–27,858,543**	1,540,202	84	54.54
	29,610,915–29,675,284	64369	3	46.61
8	26,048,184–26,572,079	523,895	23	43.90
9	6,380,344–8,089,323	1,708,979	87	50.91
	14,196,831–15,030,112	833,281	42	50.40
	15,955,700–17,602,925	1,647,225	86	52.21
10	*16,202,403–17,300,743*	1,098,340	71	64.64
	17,809,921–18,875,683	1,065,762	76	71.31
13	9,974,440–10,313,250	338,810	25	73.79

[1] GGA, chicken (*Gallus gallus*) chromosome. Regions highlighted in bold are found in over 90% of individuals, and the region highlighted in italics is common to both RK and HBSS.
